# Target Engagement Assays in Early Drug Discovery

**DOI:** 10.1021/acs.jmedchem.4c03115

**Published:** 2025-06-04

**Authors:** Sahra St John-Campbell, Gurdip Bhalay

**Affiliations:** Centre for Cancer Drug Discovery, 5053 The Institute of Cancer Research, 15 Cotswold Road, Sutton SM2 5NG, United Kingdom

## Abstract

In target-based drug discovery, quantification of target engagement is required to build structure–activity relationships and develop a potent clinical candidate. Target engagement data also provides evidence of a drug’s mechanism of action (MoA) which although is not required for approval, can increase the chance of a successful clinical outcome. Consequently, a plethora of assays has been developed to provide information about target engagement on isolated proteins and in cells. These techniques monitor changes in stability, structure, optical properties or mass difference between proteins and their complexes with ligands. They also provide characterization of the compound with thermodynamic, kinetic and structural binding parameters. The diversity of approaches reflects the challenges faced when drugging different protein classes, with each method having advantages, trade-offs and target specificity.

## Graphical abstract

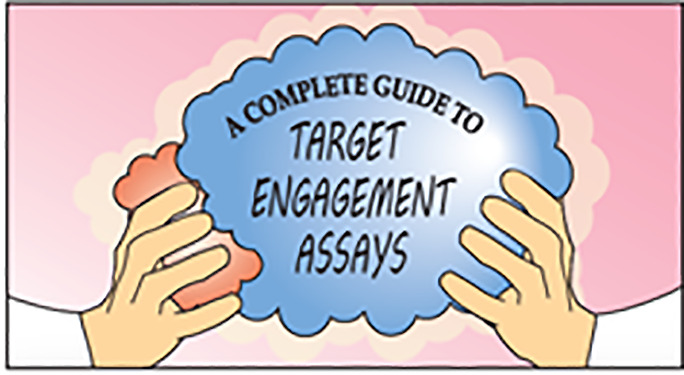

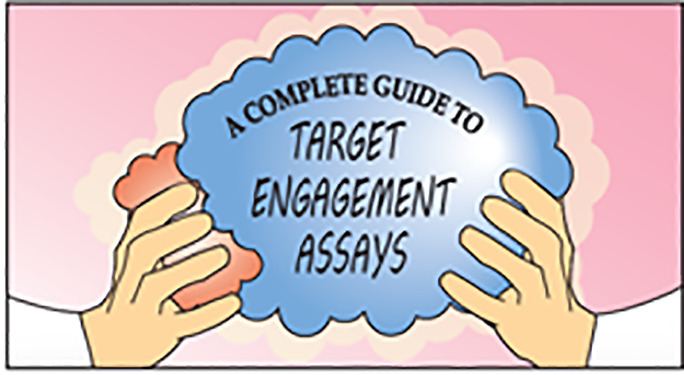

## Significance

This Perspective provides a framework for selecting target engagement assays, addressing a critical decision point in preclinical drug discovery. It will provide a key resource to enable rational, rapid and unbiased decision making in assay selection to help expedite pharmaceutical research. The key strength of this Perspective is that it encourages multidisciplinary thinking to address a key challenge in drug discovery.

## 1 Introduction

As a result of the improved understanding of the biology of various diseases, and the advances in technology improving the production, isolation and analysis of single proteins, target-based drug discovery (TBDD) has become a leading approach in the pharmaceutical industry in recent decades. After selection and validation of a biological target related to a disease,^1^ in TBDD, compounds are designed to specifically bind to the target protein to induce a therapeutic effect, ideally with minimal off-target interactions and no side effects. To facilitate this, assays need to be developed which monitor engagement (binding) between the protein and the ligand. Quantification of the strength of the interaction is also required to enable iterative improvement of a molecule’s binding to the target and to build structure activity relationships (SAR).^2^ Such assays provide evidence of a drug candidate’s mechanism of action (MoA) which has been shown to be linked to an improved clinical outcome.^3^ Accordingly, Pfizer reported target engagement as a key pillar in their 3 pillar paradigm to assess the quality of drug candidates.^4^ Similarly AstraZeneca found that confidence in target validation and biomarkers of target engagement contribute to an increased likelihood of a compound progressing through phase II trials.^5^

Target engagement can be measured by developing an assay where a ligand is added to the target protein and a quantifiable readout is generated, proportional to the degree of protein–ligand interaction. Since binding of a ligand generally results in a physical change, target engagement can be directly determined using a myriad of biophysical techniques, which are discussed in this Perspective. Target engagement can also be indirectly measured with biochemical activity assays, for example, monitoring the change in concentration of products from an enzymatic reaction,^6^ or a downstream effect of ligand binding to a receptor.^7^ Due to their high specificity for each protein, such functional assays are not discussed in this Perspective, though they remain an important inclusion in assay cascades to ensure not only that the drug is binding, but that it is also producing the desired pharmacological effect.^8^ Additionally, comparing results from functional assays with those that monitor binding directly can confirm that the effect observed is indeed due to the drug interacting with the target protein as well as indicate the efficiency of the interaction.^9^

A host of target engagement assays has been developed to scrutinize the interaction of compounds with isolated (usually recombinant) proteins. Although useful for iterative improvement of small molecules, isolated protein-based assays are highly reductive and so not very representative of how the protein may behave *in vivo*. Given the massive size and complexity of the human proteome,^10^ it is crucial to build confidence that the drug molecule specifically engages with the desired target in cells.^11^ For intracellular protein targets, in cellular assays the drug will need to enter the cell through the cell membrane (with passive or active transport) and the drug also has the potential to interact with other biomolecules within the cellular environment. Such assays therefore provide a more physiologically relevant system for measuring target engagement.^12^ Cell lysates offer an intermediary model where multiple proteins are present, but experiments in lysates do not give information about membrane permeability or downstream effects of ligand binding. This Perspective will cover methods to quantify target engagement in the early drug discovery stages of hit identification, hit confirmation and hit-to-lead stages using isolated proteins, cell lysates, and live cells. Chemoproteomics is also discussed (section 10), which is a rapidly developing field where binding of compounds is investigated not only against one protein, but the whole proteome, either in living cells or cell lysates. Although not covered in this Perspective, target engagement assays are also conducted in *in vivo* experiments, to build confidence in mechanism of action (MoA) before transferring to humans.^13^ Beyond engaging the target, assays will also be in place within the cascade to characterize compounds based on their physicochemical^14^ and absorption, distribution, metabolism and excretion (ADME) properties.^15^

In addition to building evidence of target engagement, the assays discussed in this Perspective enable the determination of the thermodynamic, kinetic and structural parameters of protein–ligand binding. Prior to discussing such assays and where they can be implemented in a preclinical drug discovery campaign, we will first provide context of these parameters, which all play a key role in guiding compound progression.

### 1.1 Drug-Target Thermodynamics

Protein–drug interactions can be described by a simple one-step binding equilibrium (Figure 1a). There are two possible equilibrium constants representing the forward and reverse of binding, the association constant (*K*_A_) and the dissociation constant (*K*_D_), which are reciprocals of each other (Figure 1b). *K*_D_ has units of molar (M) and is most frequently used to describe protein–ligand interactions. These metrics provide a description of the affinity that the ligand has to the protein; the smaller the dissociation constant (and therefore the greater the association constant), the more strongly the ligand binds to the protein, as the equilibrium is shifted further toward the protein–ligand complex. *K*_D_ values can be obtained by titration of the ligand against a protein, ensuring equilibrium is reached for each concentration (Figure 1c). The *y*-axis of the graph represents the fraction bound of the protein (θ) which is a maximum of one, where all the protein binding sites are occupied. The concentration of ligand at which half of all binding sites are bound is the *K*_D_ value, which can be estimated directly from the binding curve. *K*_D_ can therefore be determined by any binding assay where the binding is proportional to the response, as long as equilibrium is reached at each concentration. The half maximal inhibitory concentration, IC_50_, is commonly used to rank compounds in drug discovery projects, but as it is related to activity rather than binding it will not be further mentioned in this Perspective. Similarly, EC_50_ is a general term when half the possible maximum effect is induced by a ligand, whatever that effect may be.

**Figure 1:**
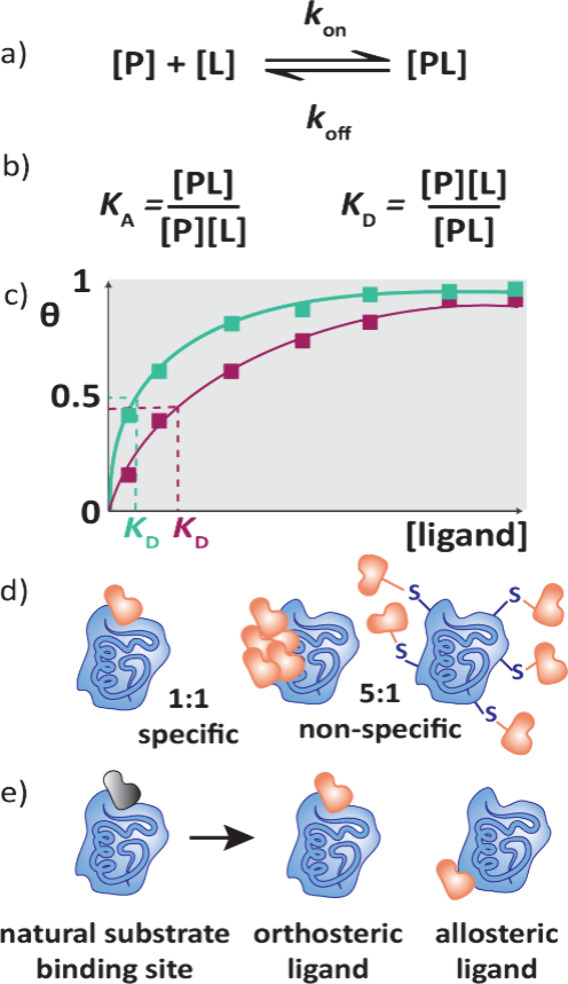
Thermodynamic, kinetic and structural parameters of target engagement. a) Protein–ligand binding can be represented by a simple equilibrium, [P] = protein, [L] = ligand, [PL] = protein–ligand complex. The rate of complex formation is *k*_on_ and dissociation is *k*_off_. b) Thermodynamic equilibrium constants *K*_A_ and *K*_D_ can be used to describe ligand binding. c) Titration of a ligand against a protein of interest enables identification of dissociation constants by finding the concentration where half of the binding sites are occupied. θ is the fraction bound of protein. The green line shows a stronger binding ligand with a lower *K*_D_ compared to the ligand shown in pink. d) Stoichiometry of the protein:ligand complex can give information about specificity of binding. e) Ligands can bind in orthosteric or allosteric sites.

The thermodynamic stabilization of a protein by a ligand can be determined by quantifying changes in enthalpy (ΔH). Enthalpy change on binding can be directly measured using Isothermal Titration Calorimetry (ITC, section 3.1) or by measuring the temperature dependence of *K*_D_ and applying the van’t Hoff method.^16^ Another indicator of the stability of a protein is the melting temperature (*T*_M_), which is the temperature at which the protein exists as a 50:50 mixture of its folded and unfolded state. Many methods (see section 3) observe changes in the thermal stability of proteins in the presence of ligands by measuring changes in *T*_M_. Notably, *T*_M_ is influenced by various complex enthalpic and entropic factors and so cannot be used to directly determine *K*_D_.^17^

### 1.2 Drug-Target Kinetics

Drug-target kinetics are governed by the microscopic rate constants for association (*k*_on_) and dissociation (*k*_off_) of the drug (Figure 1a).^18^ How long the drug spends interacting with the target, the residence time (τ), is calculated from the reciprocal of the dissociation rate constant (1/*k*_off_). It can be a predictor of *in vivo* efficacy, as drugs with larger residence times can have a longer duration of action.^18^ Covalent drugs can bind irreversibly to the target, and so their residence time is theoretically infinite, instead the effect of target inhibition is governed by the protein resynthesis rate, which can give covalent drugs a prolonged therapeutic effect.^19^ Binding kinetics can also influence selectivity; if a drug has the same *K*_D_ value for multiple receptors of the same family, but dissociates slower from one of them, it will be more selective for that protein.^18^ These factors highlight the need to not rely only on thermodynamic data to predict how the drug will behave *in vivo*, but make sure that kinetic parameters are determined early to maximize success.^18^ To determine *k*_on_ and *k*_off_ values, time-resolved data (where response vs time is observed) is needed, which is normally achieved with biosensors such as Surface Plasmon Resonance (SPR, section 4.1).

### 1.3 Structural Parameters

Stoichiometry (*N*) is the ratio of protein:ligand, i.e. how many molecules of drug bind to a molecule of protein. In most cases, a ratio of 1:1 is expected, so determination of stoichiometry in assays can identify compounds that show nonspecific binding, such as aggregators and promiscuous covalent compounds that bind to multiple protein residues (Figure 1d). Drug molecules can interact with various binding pockets of proteins (Figure 1e). The binding can be orthosteric, where it occupies the native substrate binding site, or allosteric, where it binds an alternative site on the protein. To determine the ligand binding site, X-ray crystallography (section 6.1) is the gold standard, although cryogenic electron microscopy (cryo-EM, section 8.3) is becoming competitive to X-ray crystallography in terms of throughput and resolution. Solution based techniques such as protein-observed NMR (section 6.2) and HDX-MS (section 5.3) also provide binding-site information. Competition experiments can be employed in many assays, where displacement of a ligand bound in a known binding pocket occurs on addition of the drug, providing evidence that they bind in the same site.

### 1.4 Selecting the Right Target Engagement Assay

Table 1 provides a summary of the currently available techniques that have been developed to determine direct drug-target engagement, split into the following categories: thermal techniques, biosensing, mass spectroscopy (MS), nuclear magnetic resonance (NMR), structural biology, resonance energy transfer (RET), other binding assays and chemoproteomics. A high-level overview of each technique is also provided which covers the theory behind each assay and provides references for the analysis and categorizations set out in Table 1.

**Table 1:**
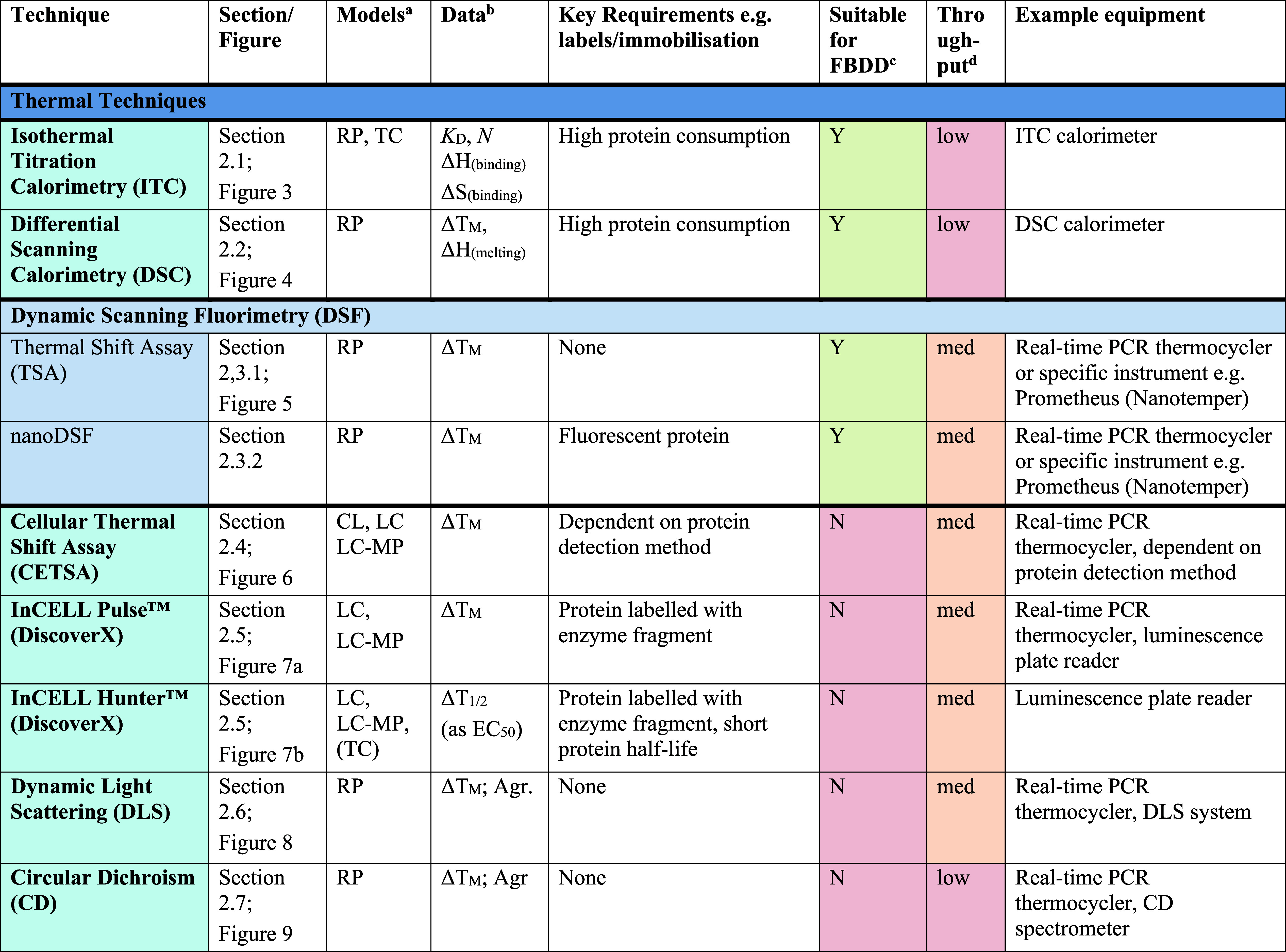

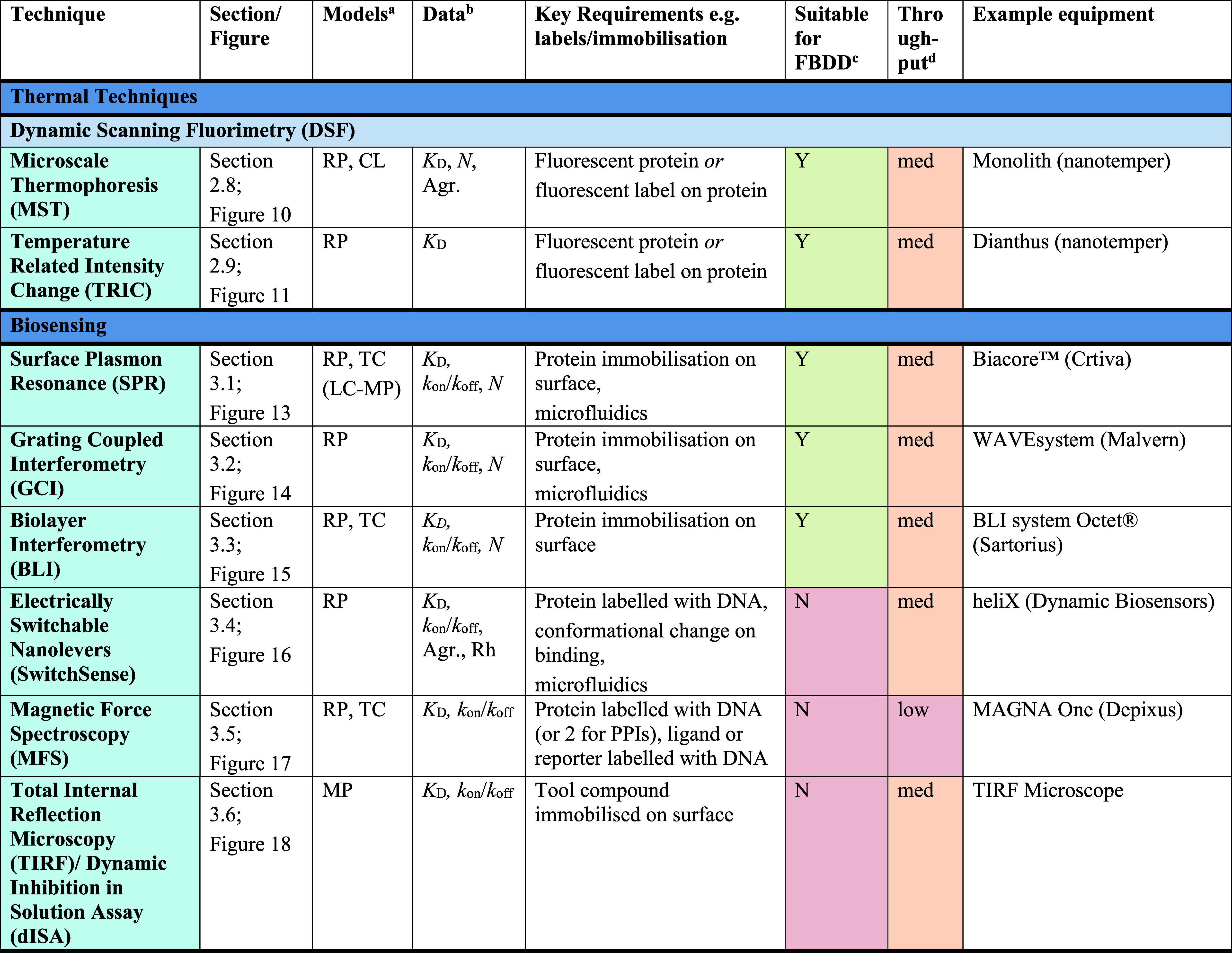

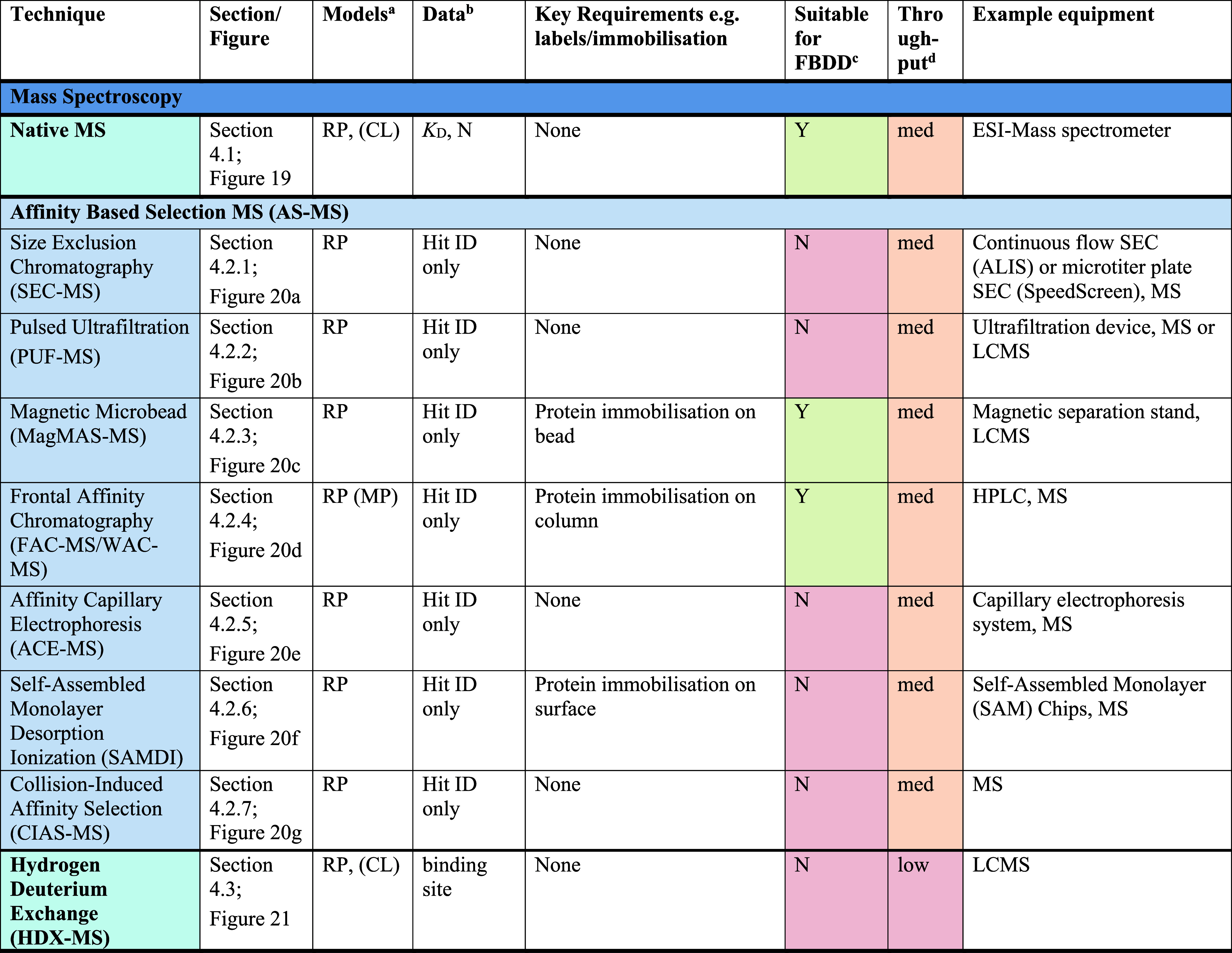

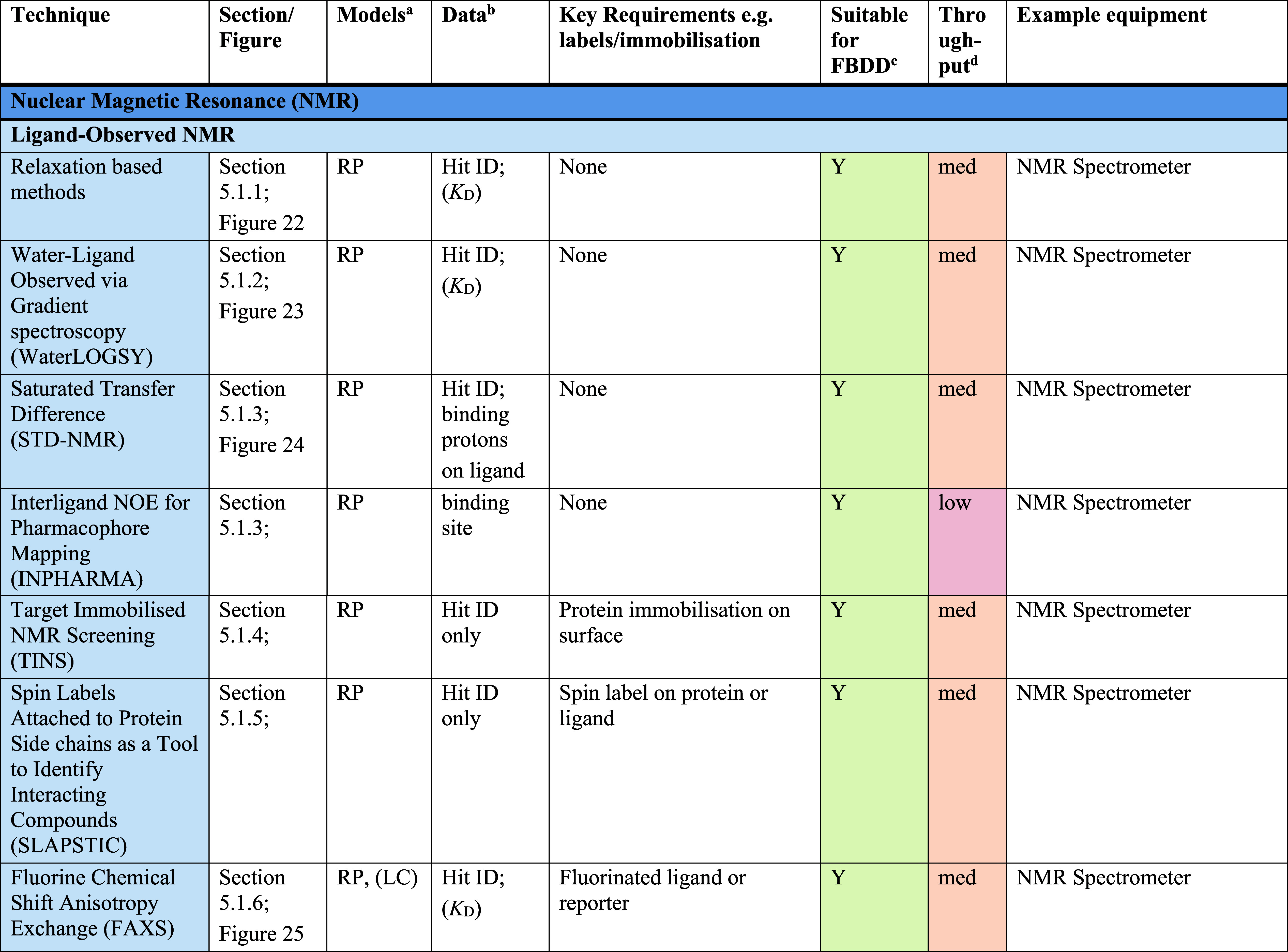

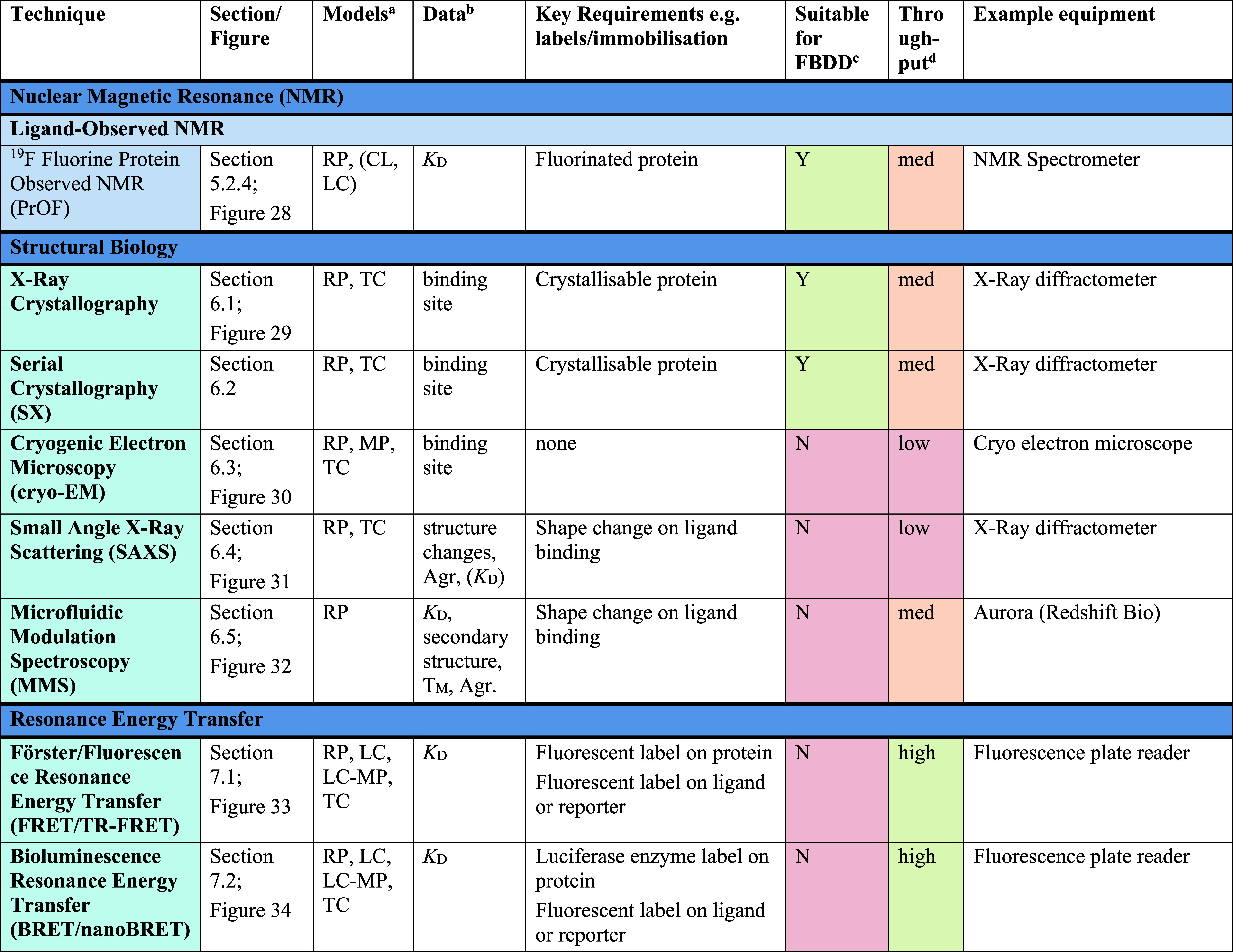

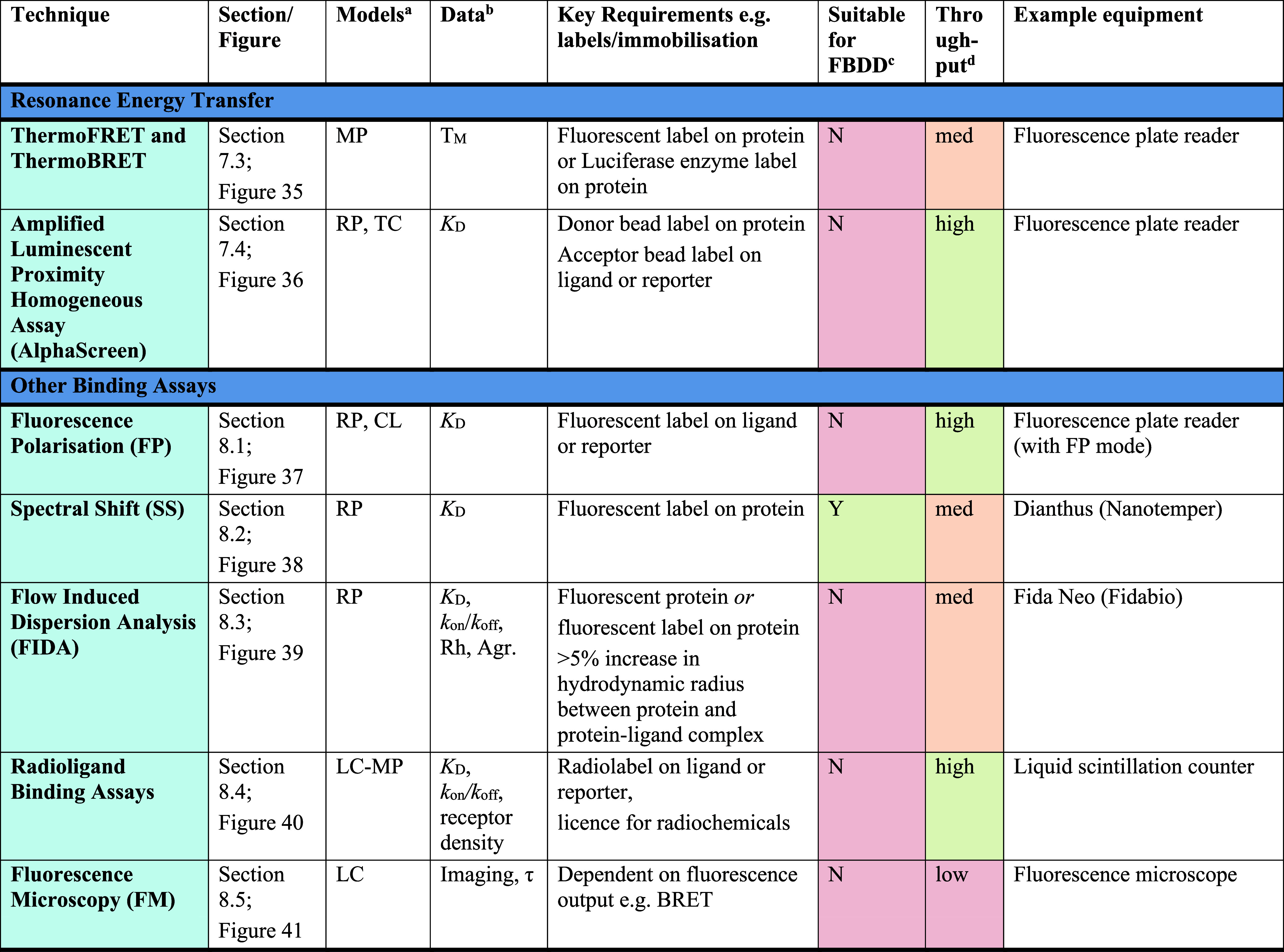

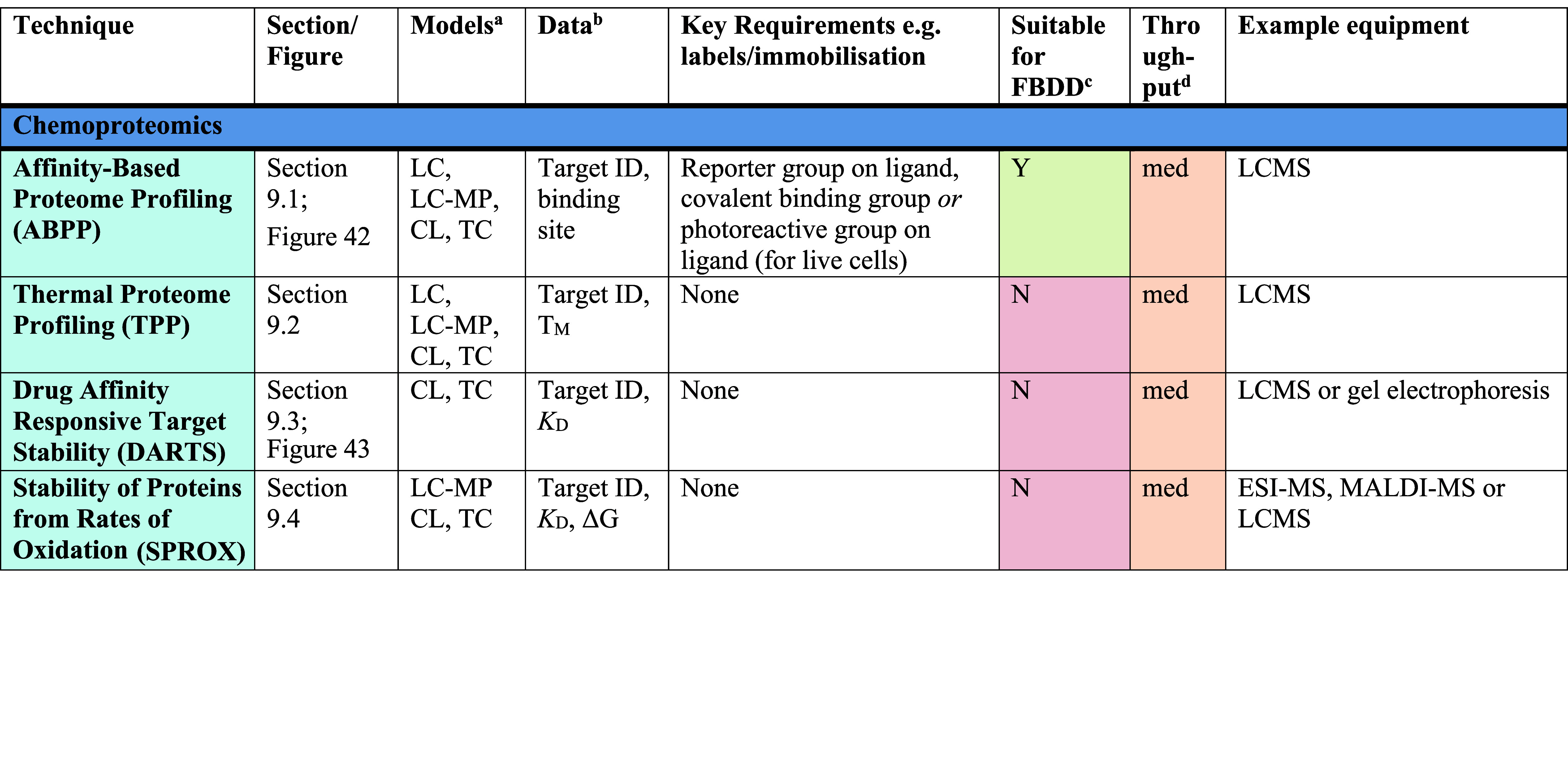
Summary of Methods to Observe Protein–Ligand Binding Covered in This Perspective

a RP = recombinant protein, MP = membrane protein (solubilized), CL = cell lysate, LC = live cells, LC-MP = live cells-membrane proteins, TC = ternary complex formation (these assays are useful for developing PPI inhibitors, molecular glues and PROTACs). Parentheses indicate some precedence but not standardly performed.

b Δ*T*_M_ = thermal shift, *N* = stoichiometry, Agr. = aggregation, Rh = hydrodynamic radius, τ = residence time. Parentheses indicate some precedence but not standardly measured.

c Suitability for FBDD is stated when the technique has been commonly used for FBDD, other methods may also be used with further optimization and using high ligand concentrations. Methods suitable for FBDD may also be useful to detect other weak protein–ligand interactions.

d Throughput descriptors are estimates and vary based on instrumentation and protocol but are broadly categorized as follows: high = regularly used for primary screening of large (>50,000) compound libraries, med = suitable for screening of small (1,000–50,000) compound libraries or hit confirmation studies and may be HTS suitable with development, low = currently useful for further compound characterization only.

For controls, assays often require tool compounds that ideally are stable, soluble (under assay conditions) and known to bind to a specific pocket of the target protein.^20^ Some assays require the use of labels/tags, which are reporter groups (such as a fluorescent dye or specific isotope) that are attached by covalent modification to the protein and/or ligand which facilitate the observation of the readout. The use of labels can result in assays with high sensitivity; however, modifications of proteins or ligands requires additional expertise and optimization. Addition of a label may also modify the binding ability and function of a protein. For these reasons, “label-free” techniques are a significant advantage. For methods using labeled ligands (FP, BRET, FAXS etc.), reporter molecules/probes are often used, which can be displaced with competitively binding ligands to observe a signal, thus enabling screening of unlabeled compounds. Protein immobilization, which is used in several assays, also requires additional optimization and can also influence protein shape and therefore ligand binding. Requirements for each assay, such as labeling, immobilization and any others specific to each assay are provided in Table 1.

Assay selection is highly project specific. It is largely dependent on both the target and the stage of the drug discovery campaign such as hit identification, hit confirmation, hit-to-lead and lead optimization. Access to specialist equipment and experience, reagents, target protein and financial cost are additional aspects to consider. Herein we discuss factors that would guide the selection of target engagement assays over the course of a standard preclinical drug discovery timeline.

#### Hit Identification

There are many ways to identify starting points, or “hits”, for drug discovery projects (Figure 2a). If hits are found experimentally, the screening approach and selection of target engagement assay can be largely guided by the target class (Figure 2b).^33^ One hit identification strategy is to screen a host of molecules against the protein of interest. In the late 1980s, screening could realistically consist of only a few hundred compounds each week. However, advances in technologies and the adoption of parallel synthesis methodology skyrocketed screening capacity, and by the mid-1990s high throughput screening (HTS) led to around half of all starting points within the pharmaceutical industry.^21,22^ In the modern day, HTS libraries can consist of millions of compounds, and to keep up with this demand, target engagement assays for HTS must be able to be suitably miniaturized and automated. In this Perspective, we define HTS applicable assays as those that can be used to screen very large (>50,000 member) compound libraries. Typically, fluorescence-based assays (FRET, BRET, AlphaScreen, FP etc.) are used because many measurements can be taken concurrently on a plate reader. Other target engagement assays can be used for compound screening of drug-like molecules, but only for focused small to medium sized libraries (see “med” throughput in Table 1).

**Figure 2:**
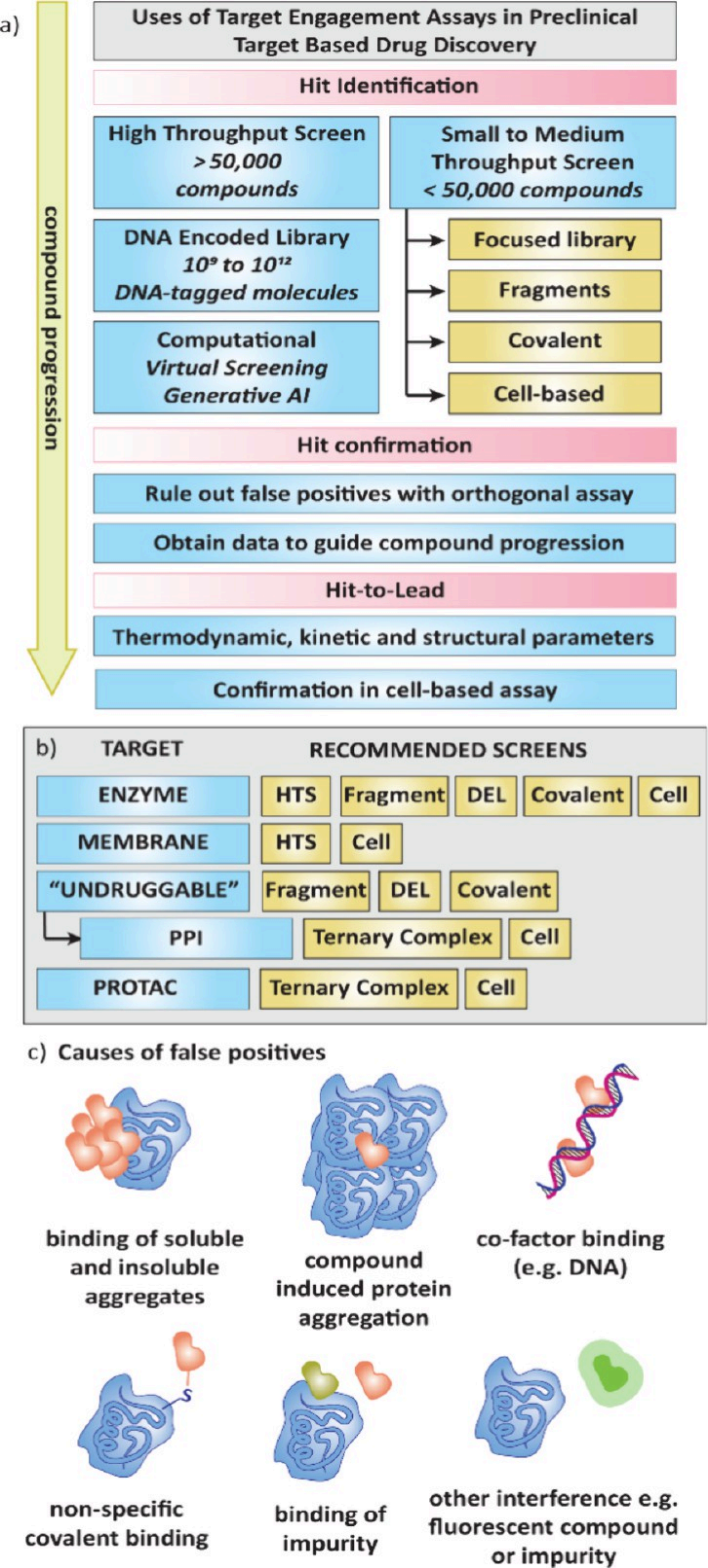
a) Target engagement assays can be used across different stages of the preclinical drug discovery timeline from hit identification with various screening strategies, to confirming hits from a primary screen as well as further characterization of compounds in the hit-to-lead stage. b) Selection of target engagement assay for screening can largely be guided by the class of target. Undruggable targets, which here we also include PPIs, often require different screening strategies than enzymes or membrane-bound proteins. c) False positives in target engagement assays can be caused by many factors.

Enzymes such as kinases are frequently targeted because they often have defined binding pockets and hits can be readily identified using biochemical or biophysical HTS.^23^ However, the majority of target engagement assays discussed in this Perspective can also be applied to enzymes. As such, if opting not to use a high throughput screen, selecting which approach and assay to use to identify hits for enzymes may be dependent on other factors.

Compounds that target GPCRs account for 30% of FDA approved drugs;^24^ membrane bound proteins such as GPCRs present an additional challenge for determining target engagement, as they are often unstable outside of the cell membrane. Consequently, hits for membrane proteins are typically found using cell-based assays; often functional assays that observe downstream effects of ligand binding.^24^ Direct cell-based target engagement assays can also be used to screen compounds for membrane proteins such as radioligand binding assays, FRET, BRET, CETSA and InCELL assays; see “LC-MP” in Table 1. Membrane bound proteins can also be solubilized into aqueous solutions with detergents,^25^ see “MP” for assays that are specifically for solubilized membrane proteins (ThermoFRET/BRET and dISA). However, with optimization of the solubilization conditions, some membrane proteins may also be used in assays designed for recombinant protein “RP”.

Drug discovery in the present day has begun to exhaust “druggable” targets which have experienced great success in finding hits from traditional HTS approaches. As a result, focus has shifted to targets previously deemed “undruggable”.^26^ These targets often lack defined binding pockets or offer the flat protein surfaces which govern protein–protein Interactions (PPIs), and so HTS using drug-like molecules has not been fruitful. Fragment based drug discovery (FBDD) presents an alternative screening strategy where smaller libraries of “fragments” (usually MW < 300 Da) are screened, which act as starting points to build toward more drug-like molecules.^27^ In FBDD, the smaller size of fragments means that superfluous functionality on larger molecules that may prevent binding is not present, so there is a greater chance of finding a hit for undruggable targets.^26^ Fragment screening is also beneficial for finding allosteric sites which may be smaller and shallower than known substrate binding pockets.^27^ Highly sensitive biophysical assays such as NMR, SPR, and DSF (TSA) are often used to detect weak binding interactions for fragment screening.^28,29^ GCI is a newer technique that may be even more sensitive than SPR to screen fragments and determine binding kinetics.^30^ X-ray crystallography is used extensively in FBDD, as atomic-level structure of the fragment binding to the protein is generally required to iteratively build fragments into potent, drug-like molecules.^29^ Table 1 provides a full list of additional target engagement assays that can be applied to fragment screening such as MST, TRIC, BLI, SS, native MS, and some AS-MS techniques.^31,32^ A successful fragment library should be structurally diverse, and each fragment must be soluble enough to enable screening at high concentrations.^27^ Six drugs derived from fragment-based drug discovery had been approved by 2021, and over 40 more are currently progressing through clinical trials.^33^

Another technique to find chemical starting points is the use of DNA Encoded Libraries (DEL).^34^ A DEL consists of typically billions of compounds that have unique DNA barcodes attached, meaning that many compounds can be screened in the same pot, and binders can be identified in complex mixtures by isolating and amplifying the conjugated DNA using a polymerase chain reaction (PCR). Due to the immense library size, DEL screening can increase the chances of finding a hit compound so can also be useful when faced with challenging targets.^34^ As well as FBDD and DEL, screening of covalent compounds is another viable strategy for targets previously thought “undruggable”.^19^ Covalent screening with libraries of electrophilic compounds is highly amenable to native MS where stoichiometry, therefore selectivity, can be determined at the outset.^19^ Challenges related to compound promiscuity can be evaluated for covalent ligands using chemoproteomics in ABPP.^35^

PPI inhibitors, molecular glues and Proteolysis Targeting Chimeras (PROTACs) involve the formation of ternary complexes, where two proteins and the ligand are brought together (or forced apart for inhibitors). Some target engagement assays can analyze ternary complex (TC) formation, which is useful as additional effects such as cooperativity can be observed, which would be missed if analyzing interactions with each protein individually.^36^ Assays that can be used to do this are indicated in Table 1 (3rd column, “TC”). Various resonance energy transfer techniques such as FRET, BRET, and AlphaScreen can be used for this purpose. These assays are high throughput however they require labels on each protein and may not be sensitive enough for fragment-based screening. SPR and BLI biosensors can be used to analyze the kinetics of ternary complex formation as well as screen compounds and fragments.^36^ DEL screening can also be adapted to be used for ternary complexes.^37^ MFS is an emerging technology which can be used to study ternary complexes at the single molecule level.^38^

Cell-based assays contain all possible cofactors which may be required for binding to complex targets, they can also detect phenotypic effects (such as cell viability) alongside target engagement.^39^ Cell-based target engagement screening offers a great advantage in that (for intracellular targets) chemical starting points are shown to be cell permeable at the outset. For these reasons, hits found through cell-based screens are more likely to succeed in patients.^11^ However, cell-based assays are more complex, requiring cell culture to be optimized, they often have higher variability and lower throughput. Most cell-based assays are also less sensitive which can be problematic if used for fragment screening or if weak interactions are expected. CETSA is a commonly used cell-based assay for screening, though TR-FRET, nanoBRET, InCELL assays as well as unbiased screening using chemoproteomics can also be used. For additional options, see assays which can be used with live cell “LC” or cell lysate “CL” models in Table 1.

Availability of instrumentation also plays a considerable role in choosing which target engagement assay to use, with some techniques being well established and requiring minimal equipment and thus receiving greater uptake. For example, assays that use fluorescence as the output (FP, BRET, FRET) only require a plate reader, and TSA requires a real-time PCR thermocycler. NMR and MS instruments are also available at many institutions. SPR does require specific instrumentation, though a selection of SPR systems from different suppliers are now available, increasing accessibility to the point where SPR is now routinely used.^40^ Emerging technologies can provide data-rich results on ligand binding with improved throughput and/or sensitivity. Examples include GCI (WAVEsystem by Malvern), SwitchSense (heliX by Dynamic Biosensors), SS (Dianthus by Nanotemper), MFS (MAGNAone by Depixus), MMS (Aurora by Redshift Bio) and FIDA (Fida Neo by Fidabio). If these techniques prove to be advantageous compared to established techniques in future applications, their use will grow in coming years. However, only one instrument is presently available for these techniques, which could limit uptake, particularly if they are also of high cost.

If protein availability and consumption is a concern, then assays that do not require purification of the protein may be beneficial, such as assays which work in live cells (CETSA, nanoBRET) or lysates. Assays that can be highly miniaturized could also be suitable (TR-FRET, MST, SS etc.).

Although not discussed in this Perspective, advances in computational power and the development of improved modeling techniques, has meant that accuracy in finding chemical starting points using virtual screening approaches with docking or pharmacophore models is becoming increasingly reliable.^41^ *De novo* design of molecules using generative AI is also playing a growing role in drug discovery for many pharmaceutical companies.^42^ Phenotypic screening against a disease model is also possible and may have some benefits in terms of later clinical success.^43^

Figure 2b provides a summary of protein target classes and suggested screening strategies. Whereas many approaches can be used to find hits for enzyme targets, undruggable proteins may need different tactics to find hits such as fragment, DEL or covalent screening. If the potential drug is involved in ternary complex formation such as PPI inhibitors, molecular glues or PROTACs, an assay that analyses this complex, or alternatively a cell-based assay, is recommended.

False positives in screening campaigns add to the rising costs of drug discovery and delays in providing much needed treatments for patients (Figure 2c).^44^ False positives can be caused by multiple reasons such as nonspecific binding of precipitated or soluble compound aggregates, particularly if the molecule has high lipophilicity or has a high level of aromaticity.^45^ Compounds and their aggregates can also lead to aggregation of the protein.^46^ Aggregation can scatter light signals and produce false results in assays with fluorescence readouts, though adding detergents can minimize this effect.^47^ Compounds may also bind to cofactors present in the assay such as DNA.^48^ Molecules with electrophilic groups can form adducts with nucleophilic amino acid residues on the protein.^49^ False positives can also be a result of the presence of impurities in the compound sample, or other interference such as background fluorescence, specific to the assay used. Pan-Assay Interference Compounds (PAINS)^50^ are now being identified and omitted earlier in drug discovery campaigns, though identification of false positives through all possible mechanisms is still paramount and often achieved through hit confirmation in multiple orthogonal assays. As drug discovery moves to undruggable targets with lower ligandability,^26^ it is becoming increasingly important to quickly rule out an increasing number of false positives from screening campaigns.

#### Hit Confirmation

Whatever strategy was used in hit identification, as best practice all hit compounds should be confirmed in an orthogonal assay to rule out any false positives. The orthogonal assay should be suitably distinct from that used for screening; for example, if hits were found with SPR, the confirmation assay *should not* also use protein immobilization (GCI, BLI etc.). If a biochemical assay was used in screening, for example one that measures the inhibition of an enzymatic reaction, a direct target engagement assay such as those listed in this Perspective would provide confidence that the drug is binding the target and not a cofactor in the biochemical assay preventing the enzymatic reaction. Similarly, if the compound was found using a cellular screen, evidence of specific binding should be demonstrated to rule out off-target mechanisms. Aggregation is a common cause of false positives and so ruling out this mechanism of assay interference by using a technique that can determine stoichiometry (ITC, native MS, SPR etc.) or observe aggregation directly (DLS, MST etc.) is worth considering.^45^ If hits were found using information poor techniques such as those that measure *T*_M_ (TSA, CETSA, TPP, InCELL Pulse), or only show binding in a qualitative manner (LO-NMR, AS-MS) then the orthogonal assay would add value by also measuring other parameters such as affinity (*K*_D_) and kinetics of binding (*k*_on_/*k*_off_). This will front-load information in early stages which may aid in deciding which compounds or series are most desirable to take forward into further development. Obtaining a crystal structure by screening or hit confirmation using X-ray crystallography or cryo-EM is a huge advantage by enabling rational structure based drug design (SBDD).

#### Hit-to-Lead and Lead Optimization

Assays developed in the earlier stages of the drug discovery program will continue to be used to guide the development of lead compounds. However, when molecules show promise, additional target engagement assays can be introduced to measure missing parameters, build further confidence in specific target engagement and show target engagement in cells.

The summary of assays in Table 1 is intended to support rational, unbiased decision making when selecting which target engagement assay may be suitable for a particular drug discovery program. Herin, further reading for each direct target engagement assay is provided, covering the science of each technique and further commentary on its applications, benefits and limitations.

## 2 Thermal Techniques

In this article, thermal techniques have been characterized by those that involve temperature changes. This includes methods which use calorimetry (ITC and DSC) as well as those that observe the melting temperature (*T*_M_) of proteins (DSC, DSF, CETSA, InCELL Pulse, DLS, and CD), or exploit changes in thermophoretic mobility (MST) or changes in fluorescence of a dye at different temperatures (TRIC). Thermal methods are solution based and do not require immobilization of the target or ligand on a surface.^20^ This makes them simple to set up and amenable to many different systems. The Thermal Shift Assay (TSA), a type of Differential Scanning Fluorometry (DSF), is the most used thermal technique for screening, and cellular TSA (CETSA) has also been developed. Isothermal Titration Calorimetry (ITC) and Differential Scanning Calorimetry (DSC) are useful for determining enthalpy values but are low throughput and so more amenable to hit validation and characterization.

### 2.1 Isothermal Titration Calorimetry (ITC)

Binding of a ligand to a target is accompanied by an enthalpy change which results in the release of heat to, or absorption of heat from, the surrounding solution. The resulting temperature change can be measured using isothermal titration calorimetry.^51^ ITC is a very simple technique requiring no immobilization or labels, just the protein and ligand in solution. It requires no assay development thus gives relatively rapid results on the thermodynamics of ligand binding.

In ITC, two cells are enclosed in an adiabatic shield to minimize heat transfer between them (Figure 3a). This consists of the sample cell, containing protein solution, and a reference cell of buffer. These two cells are initially heated to the same temperature. With stirring, aliquots of ligand are sequentially added to the protein solution in the sample cell, causing a temperature change. As the temperature of the sample cell changes, the power supplied to heat this cell is modified until once again it reaches the same temperature as the reference. Initially the temperature change will be large as all the analyte injected will bind, but as further aliquots are added this will reduce as protein saturation is approached. This is continued until there is no more temperature (and thus power) change, meaning the protein is fully saturated with ligand.^51^

**Figure 3:**
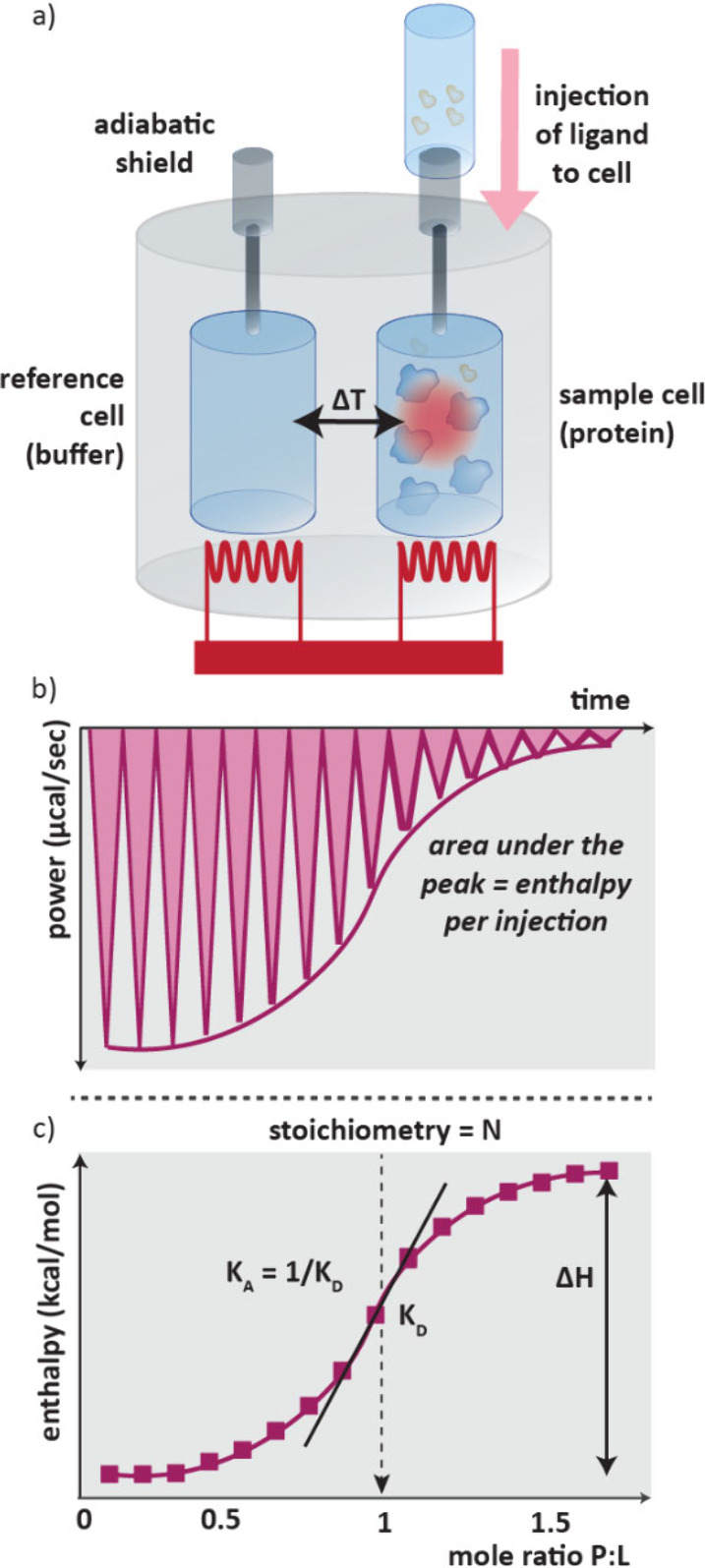
a) Setup of an ITC experiment. Aliquots of ligand are added to a solution of protein, this causes an exotherm which is measured. Further ligand aliquots are added until saturation of the protein is reached. b) Raw data from an ITC experiment showing spikes in power caused by temperature changes on addition of each aliquot of analyte. c) Plot of the integrated enthalpy values per injection allows stoichiometry and thermodynamic parameters to be determined.

The raw data from an ITC assay is a plot of the power required to maintain Δ*T* = 0 between the reference and sample cell over time (Figure 3b). With each aliquot, the temperature difference between the cells peaks then returns to the baseline. Notably, if the binding is endothermic, the power supplied will increase.^52^ The area under these peaks gives the enthalpy per injection which can be plotted against the P:L molar ratio and the thermodynamic constants, stoichiometry and total enthalpy change of binding can be determined (Figure 3c).

ITC is the gold standard in direct binding measurements, but despite advances in improved sensitivity of calorimeters, it still needs milligrams of protein and high compound solubility.^53^ It has not been used in cell-based analysis and is low throughput so cannot be used in screening efforts.^20^ Despite this, ITC is a useful technique for secondary hit confirmation and detailed hit characterization,^51^ including for ternary complexes.^36^

### 2.2 Differential Scanning Calorimetry (DSC)

As proteins are heated, they eventually reach a transition point at which unfolding (denaturing) occurs. The point at which there is a 50:50 mixture of folded and unfolded protein is referred to as the melting temperature, *T*_M_. This process breaks intramolecular bonds in the protein structure, resulting in an exotherm. In DSC, a sample of protein is subjected to a temperature gradient and the exotherm that occurs at the melting temperature is recorded.^54^

DSC requires two sealed cells, a sample and a reference, which are gradually heated in an oven (Figure 4). The difference in temperature of the sample and the reference cell is then measured. To normalize the data by mass, the enthalpy change is used to calculate the excess heat capacity (sample minus reference) which is plotted against temperature (Figure 4b). When a phase transition occurs, unfolding of a protein in this instance, there will be heat released (an exotherm) at the temperature of the transition. This enables determination of the onset temperature (*T*_onset_), *T*_M,_ as well as the enthalpy change of the transition, which is the area under the curve. By repeating this experiment in the presence of a ligand, the change in melting temperature and enthalpy can be determined, with more stabilizing ligands giving a greater increase in *T*_M_.^55^ Like ITC, sensitivity of DSC has increased over the years, but it is still low throughput and thus not useful for compound screening.

**Figure 4:**
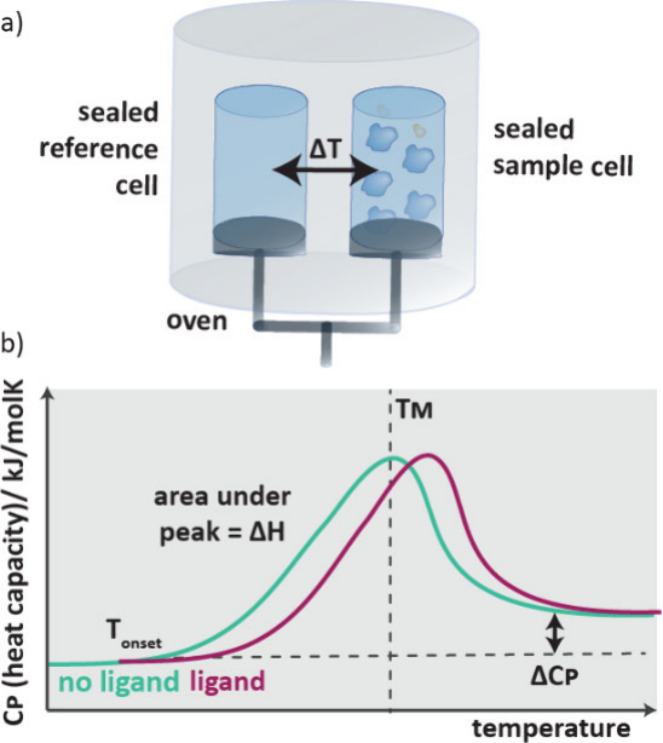
a) Setup for a differential scanning calorimetry experiment. A cell containing protein and ligand is gradually heated and exotherms are monitored compared to a buffer solution. b) Data from a DSC experiment is normalized heat capacity (sample minus reference) vs temperature. Exotherms with or without ligands can be compared.

### 2.3 Differential Scanning Fluorometry (DSF)

DSF is where fluorescence is monitored upon changes in temperature. DSF can be used to measure *T*_M_ by observing fluorescence changes that occur on protein unfolding, either of inherent protein fluorescence (nano-DSF) or of an added dye (Thermal Shift Assay). When a ligand binds to a protein, this often causes a stabilization effect due to favorable intermolecular interactions, resulting in an increase in *T*_M_. The stronger the ligand binds, the higher the degree of stabilization and the greater the change in *T*_M_. Ligands can also cause a destabilizing effect which can be observed as a decrease in *T*_M_.^20^ As with DSC, DSF cannot be used to determine *K*_D_ values due to the various factors which influence *T*_M_. Despite being an information poor technique, DSF is still very useful in determining the change in protein stabilization to screen compounds across a wide range of affinities and a throughput as high as 1536 ligands per hour on a single plate.^17^

#### 2.3.1 Nano-DSF

If the protein of interest contains tryptophan or tyrosine residues it has native fluorescence which changes upon denaturing. Upon heating, the change in inherent fluorescence can be measured using a fluorometer which enables determination of the melting temperature; this method is called nanoDSF.

#### 2.3.2 Thermal Shift Assay (TSA)

For proteins without natural fluorescence, or if the fluorescence change is small and thus difficult to observe, a fluorescent dye is added so that *T*_M_ can be determined. The fluorescent properties of the dye must change in the presence of folded or unfolded protein (Figure 5a). Thermal shift assays have the benefit of being relatively simple to set up, as they only need protein and dye, avoiding the high cost of expensive biochemical reagents. They also can be carried out using standard thermocyclers to monitor temperature with fluorescence, which are used for real time polymerase chain reactions (RT-PCR).^21^

**Figure 5:**
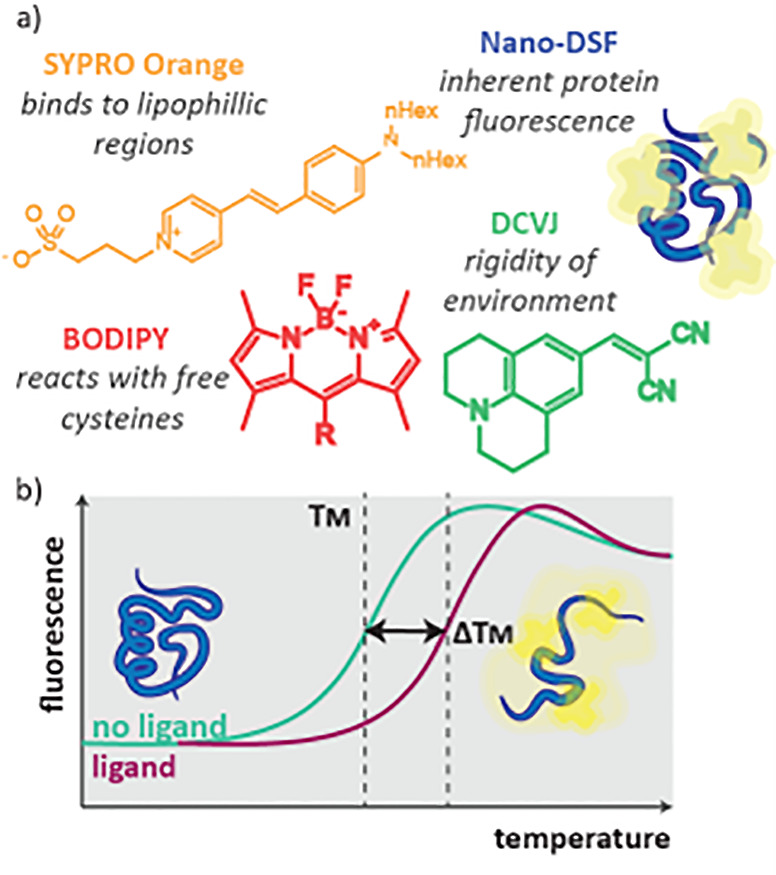
a) Structure and function of dyes used in TSA. b) Data from a TSA experiment. Fluorescence increases as the protein unfolds; a slight decrease is observed as proteins begin to aggregate at higher temperatures.

The direct readout of a TSA experiment is a plot of fluorescence versus temperature (Figure 5b). When using dyes which fluoresce more brightly in the presence of unfolded protein, such as Sypro Orange, the amount of fluorescence will increase with temperature as the protein unfolds. When all the protein is unfolded a maximum fluorescence is reached. Aggregation of the protein at higher temperatures causes a slight drop in fluorescence output due to dissociation of the dye. The point at which 50% of the protein is denatured is the *T*_M_. The experiment is repeated in the presence of ligands at various concentrations, and the change in melting temperature, or thermal shift (Δ*T*_M_) is recorded.

Thermal shift assays can be miniaturized to use in compound screening. This is particularly useful due to the generality of the assay, and that only minimal information is needed about the protein function as only binding properties are measured.^56,57^ Owing to its high sensitivity, TSA is highly amenable to use in FBDD.^31^

### 2.4 Cellular Thermal Shift Assay (CETSA)

Experiments to measure Δ*T*_M_ (the thermal shift) in cells were first developed in 2013 by Martinez Molina and colleagues.^58,59^ CETSA measures drug–target interactions by quantifying the amount of folded protein remaining in a cell following heat shock. This is related to the *T*_M_ of the target protein which can be influenced by the degree of affinity a ligand has to the protein.^56^ In seminal examples, intact cells or cell lysates were heated to a range of temperatures, aggregated proteins were then removed by centrifugation, and the remaining soluble folded protein was detected by a Western blot assay (Figure 6).^58^

**Figure 6:**
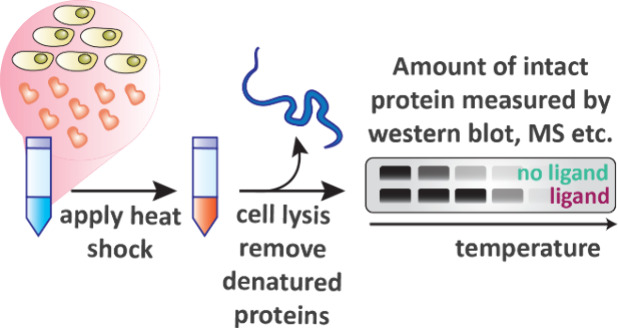
Workflow of CETSA. Cells incubated with ligand are heated to various temperatures and the amount of intact protein of interest remaining is analyzed.

Further development of CETSA has diversified readouts. Now, as well as Western blot, mass spectroscopy, Alpha-LISA and HiBit (BiTSA) tagging can also be used.^60^ CETSA has successfully been used in library screening by several groups using a variety of protein detection methods.^12,56^ It can also be used for membrane proteins in live cells.^61^ CETSA can also be used in *in vivo* or *ex vivo* formats.^62^

### 2.5 InCELL Pulse and InCELL Hunter

In 2017 Eurofins DiscoverX reported cellular target engagement assays InCELL Pulse and InCELL Hunter.^63^ Like CETSA, InCELL pulse determines the amount of intact protein present after heat shock, which is increased in the presence of stabilizing ligands (Figure 7a). InCELL Hunter, however, observes the steady-state turnover of normal protein synthesis, accumulation and degradation (Figure 7b). In principle, the ligand will stabilize proteins so that the concentration of intact protein accumulated will be higher than in the absence of ligand. InCELL Hunter requires the protein to have a short half-life so that its stabilization by ligands can be observed in a suitable time frame.

**Figure 7:**
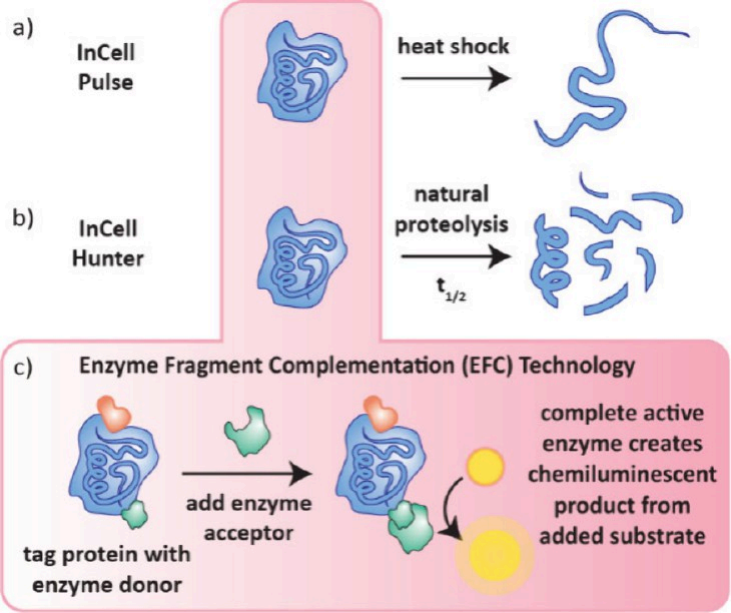
InCell Pulse and InCell Hunter assays. a) For InCell Pulse, the amount of intact protein present after heat shock is measured. b) For InCell Hunter, the steady-state concentration of intact protein in its natural cycle of synthesis, accumulation and proteolysis is measured. c) Both assays require the protein to be tagged with an enzyme donor which at the end of the assay can be complimented by an enzyme acceptor portion to form a catalytically active enzyme which turns over a chemiluminescent substrate.

In both assays, the amount of intact protein present is quantified by chemiluminescence using Enzyme Fragment Complementation (EFC) technology (Figure 7c). To facilitate this, InCELL assays require labeling of an enzyme fragment to the protein of interest prior to expression in cells. At the end of the assay, the amount of intact protein is measured by addition of a complementary enzyme portion which creates a catalytically active enzyme (β-galactosidase) that can turnover a prechemiluminescent substrate resulting in light emission. In the absence of intact folded protein, the enzyme fragment cannot bind the complementary enzyme portion and so the chemiluminescent compound is not formed and no light emission is observed. Both InCELL pulse^63^ and InCELL Hunter^64^ have been shown to be suitable for compound screening.

### 2.6 Dynamic Light Scattering (DLS)

Aggregated proteins scatter light more intensely than proteins in their natural folded state, which can be measured using light scattering.^65^ Light scattering can be measured using static (SLS, at one time point at a range of angles or concentrations) or dynamic (DLS, one or multiple angles over time) techniques. For a DLS experiment, laser light is scattered by a solution of protein (Figure 8a) and the intensity of scattered light over time is recorded (Figure 8b). This raw data is processed to give a distribution of particle size in the sample.^65^ To find *T*_M_ values, the samples are heated and changes in light scattering are detected.

**Figure 8:**
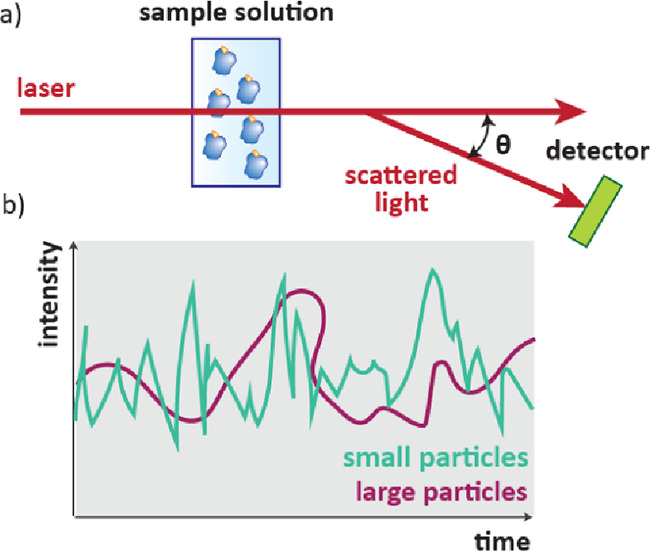
DLS of protein–ligand complexes in solution. a) A laser beam is scattered by freely rotating proteins in solution and the intensity is measured by a detector. b) Different sized particles will have distinctive scattering intensity vs time profiles.

As DLS can be used to monitor the aggregation (hence thermal denaturing) of proteins, it can also be used to compare their thermal stabilization by ligands. DLS has been used in compound screening for this purpose, however compared to TSA it is not very sensitive and is only able to detect changes in *T*_M_ greater than 0.5 °C.^66^ DLS is also a useful tool to identify small molecule or protein aggregation, a common cause of false positives in screening campaigns.^65^

### 2.7 Circular Dichroism (CD)

The unfolding of proteins with temperature can also be measured using circular dichroism and absorbance spectroscopy.^67^ CD observes the difference in absorption between left and right circularly polarized light. Proteins have well-defined chiral structures and so have a large CD signal. When they are denatured, however, random free rotation around the bonds results in a smaller CD signal.^68^ In CD experiments, left or right circularly polarized monochromatic light is created and passed through a sample of protein (Figure 9a), and the difference in absorbance of left and right polarized light is recorded (Figure 9b). The results are repeated at different temperatures to create a temperature vs fraction unfolded graph which can be compared with and without ligands.

**Figure 9:**
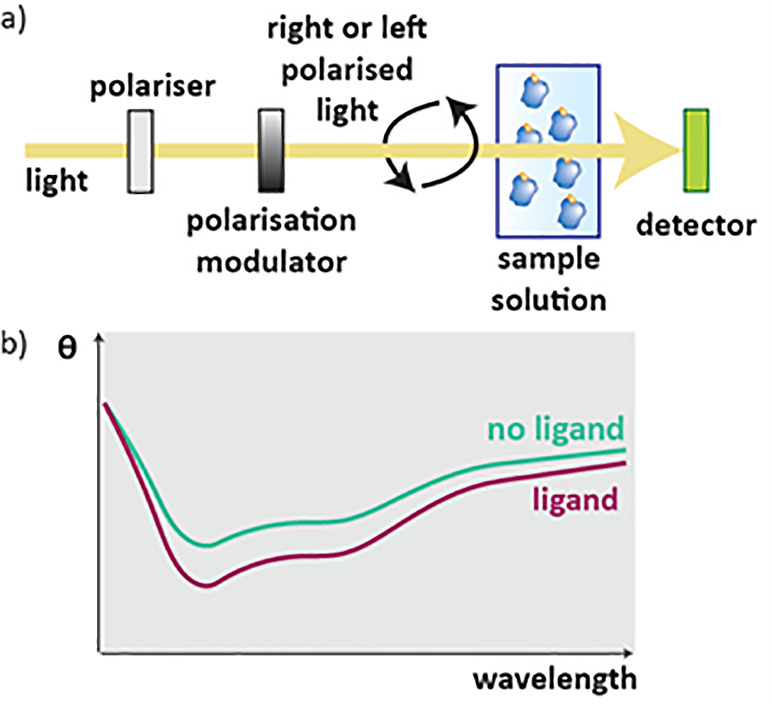
CD of protein–ligand complexes in solution. a) The difference in absorbance in left or right polarized light after passing through a sample is detected. b) The results from a CD experiment are CD (θ) versus wavelength, unfolded proteins will give a weaker signal.

Although CD systems with improved throughput are in development,^69^ its applications for screening ligands have not yet been fully realized.

### 2.8 Microscale Thermophoresis (MST)

MST uses the concept of thermophoretic mobility, the directed movement of molecules along a temperature gradient which is influenced by the size, charge and hydration shell of the molecule in solution.^20^ MST was first developed in 2011 and it monitors the local change in fluorescence of solutions of proteins or protein–ligand complexes as they diffuse away from an infrared laser-heated spot.^70^ Binding of the ligand affects the thermophoretic mobility of the protein, and so MST can be used to determine *K*_D_ values. However, the largest contribution in an MST trace may well be due to the reduction in the fluorescence of a molecule with increased temperature, as in a TRIC assay (see section 3.9).^71^

To run an MST experiment, protein solutions are prepared in capillaries with different ligand concentrations. A local temperature difference ΔT is induced on each capillary in sequence by an infrared laser, this leads to a change in local molecule concentration. The concentration of the protein in that spot is determined by fluorescence of either the native protein or a fluorescent label tethered to the protein (Figure 10a).

**Figure 10:**
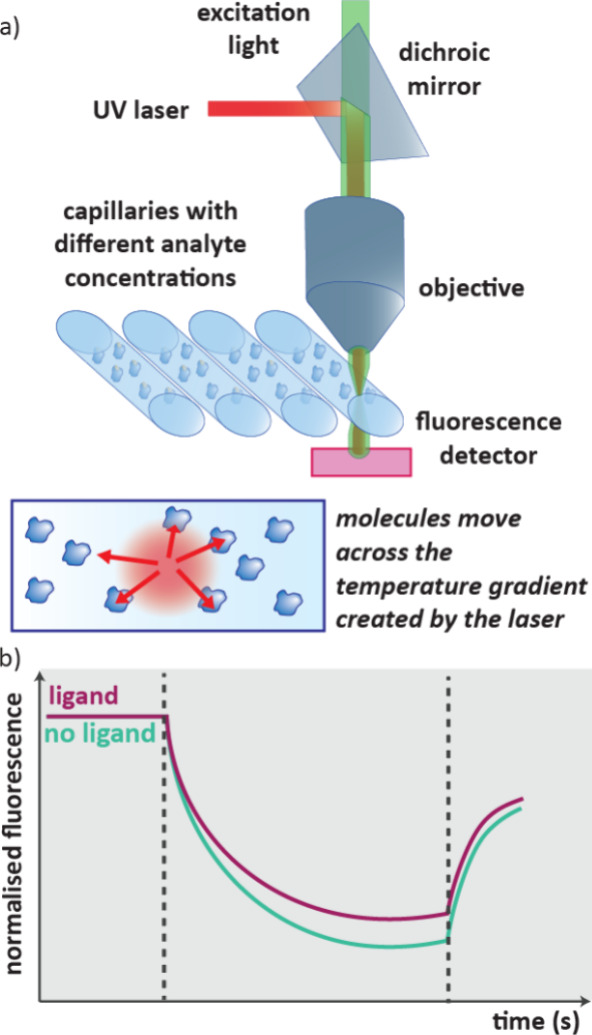
a) Setup of an MST experiment. Capillaries of varying ligand concentrations are scanned with an IR laser and fluorescence is monitored. Thermophoretic mobility is the movement of molecules across a temperature gradient, which is induced by an IR laser in MST. b) Data from an MST experiment. As the laser is switched on, fluorescence decreases as proteins diffuse away from the detector, when the laser is turned off, proteins gradually diffuse back.

The readout of the MST experiment is fluorescence against time, upon switching on the infrared laser, fluorescence decreases as the protein molecules diffuse away (Figure 10b). When the laser is turned off the fluorescence response increases as the molecules diffuse back across the concentration gradient. In the presence of a bound ligand, the movement of the protein will change. To determine binding affinity, the normalized fluorescence is plotted against the concentration of ligand.^72,73^ Information on protein aggregation and denaturation can be obtained from the shape of the MST trace.^74^

A significant downside of MST is that proteins are not often suitably fluorescent, meaning a fluorescent label is needed on the protein. In addition, because each molecule can give a different response in MST, in its native form it is not suitable for primary screening, where cutoff values are used to determine ligand binding. To overcome this drawback, competition experiments can be used.^72^ MST has been shown to be suitably sensitive to detect weak binding from fragments.^74^ Additionally, only low sample concentrations are needed (capillaries can be less than 4 μL)^75^ and it can be used in complex mixtures such as cell lysates and detergents.^20^

### 2.9 Temperature Related Intensity Change (TRIC)

The principal of TRIC assays is that the fluorescence of a dye molecule tethered to a target protein changes, usually decreasing, with increased temperature (Figure 11a). This change in fluorescence with respect to temperature is influenced by the microenvironment of the dye and therefore the binding of a ligand to the target protein can be monitored. This phenomenon is a large component of the signal in an MST trace, and in practice the workflow of the assay is very similar to MST where an IR laser heats a sample and monitors fluorescence over time (Figure 11b).^71^ Unlike MST that requires capillaries, TRIC measurements can be conducted in microtiter plates, increasing throughput, and the technique has been shown to have suitable sensitivity for fragment screening.^76^

**Figure 11:**
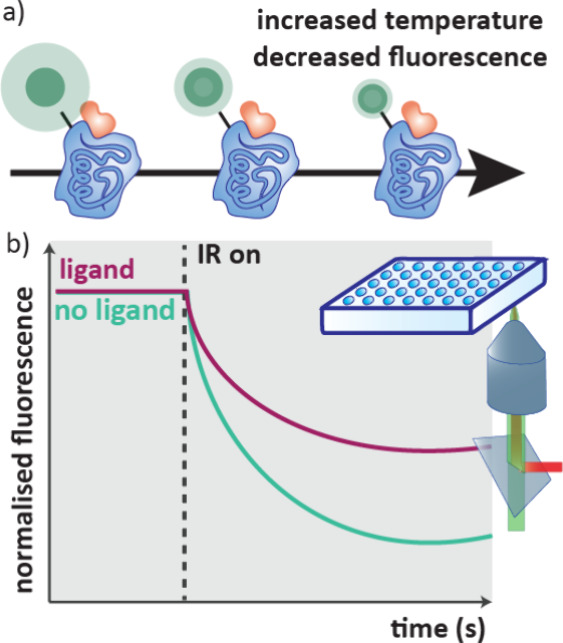
a) Principle of a TRIC assay, changing the temperature influences the fluorescence of a dye tethered to a protein. b) In a TRIC experiment, sample solutions are heated with an IR laser and the fluorescence in the presence of differing ligand concentrations is measured in real time.

## 3 Biosensing

Biosensors are devices that consist of a biological sensing element that can detect the presence of chemicals or other biomolecules.^77^ Biosensors are widely used in medical diagnostics and chemical detection and are used in drug discovery to identify and characterize target engagement. The terminology typically used for biosensors is different than other methods, with the immobilized protein often referred to as the ligand and the small molecule ligand as an analyte. As interactions between biosensors and their analytes can be detected in real time, they can be used to determine kinetic parameters *k*_on_ and *k*_off_. Additionally, biosensors typically require only low concentrations of protein and analyte and for the most part benefit from being label-free, eliminating the need for fluorescent, radiolabeled or chemical tags.^78^ Most biosensors require immobilization of the target protein on a surface. A common way to do this is by using a gold surface with a carboxymethyl (CM) dextran matrix (Figure 12a), where free carboxyl groups on the dextran surface can bind to protein residues, although many other surfaces are possible and new methods to immobilize proteins are in continual development.^79^

**Figure 12:**
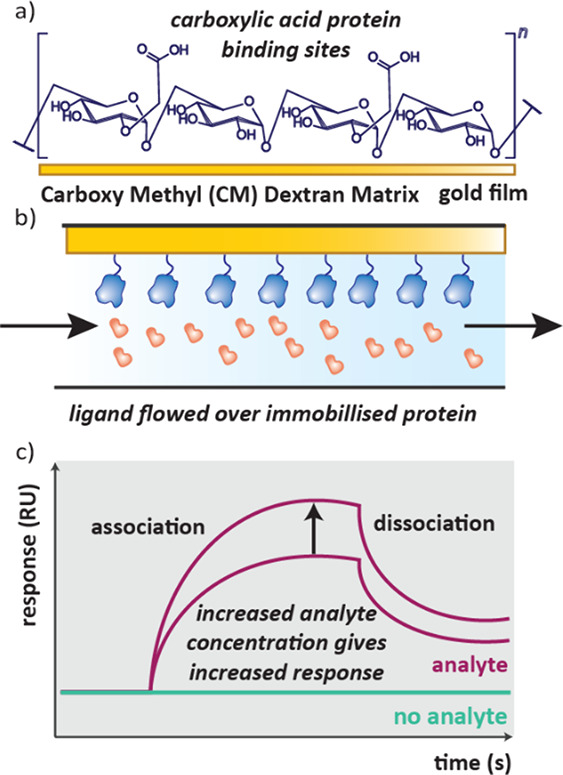
a) A CM dextran matrix on a gold surface can be used to capture proteins for biosensing applications. b) Biosensors use microfluidic systems to flow the analyte over the immobilized protein surface. c) A typical response readout from a biosensor, the response increases as the analyte is flowed over the surface until a maximum is reached. When buffer is added to wash away the analyte, the response decreases as the compounds dissociate.

Biosensors often rely on microfluidic systems.^80^ The protein is first immobilized on a surface from a solution, and excess unbound protein then washed away. To take the measurement, analyte can be flowed across the surface containing the protein (Figure 12b) and to conclude the experiment, the analyte is washed off with buffer. This is repeated with different analyte concentrations and analytes. In some cases, the surface can be recovered by cleaving off the protein.

The most ubiquitously used biosensor in drug discovery is SPR (section 4.1), however many different sensing technologies have been employed. In general, it is the change in refractive index of the biolayer which causes the changes in response of the biosensor.^78^ Differences in refractive index are related to mass changes, in this case when an analyte interacts with a surface bound protein. Readouts often appear similar among biosensors, with response from the sensor increasing on ligand binding up to a maximum and subsequently decreasing as the analyte is washed away from the surface (Figure 12c). The shape of the response vs time curve can provide information about mechanism of binding, for example if it is mass transport limited or if the analyte irreversibly binds to the surface.

Biosensors require tethering of the protein on a surface therefore establishing new assays can be labor intensive. Tethering may also result in a change in the binding properties of the protein. Due to the microfluidic systems, artifacts related to mass-transport limitations are also possible.^72^

As well as those covered here, other biosensors have also been developed. These include Mach–Zhender Interferometry (MZI),^81^ Young’s Interferometry (YI),^82^ Dual Polarization Interferometry (DPI),^83^ Surface Acoustic Waves (SAWs),^84,85^ Epifluorescence (EPF)^85^ and the Quartz Crystal Microbalance (QCM).^86^ However, the use of these technologies to observe protein–ligand interactions for drug discovery has been highly limited to date, so they are not further discussed. Similarly, the Resonant Mirror (RM) biosensor was first commercialized in 1993 however has largely been discontinued from use.^87^ This is also the case for the Second Harmonic Generation (SHG) biosensor which was commercialized as the Biodesy Delta system in 2016.^88,89^ Resonant Waveguide Grating (RWG) biosensors are used much less commonly than others, however they have been developed to monitor protein–analyte interactions for compound screening.^90^ Back Scattering Interferometry (BSI) was first developed to monitor interactions between proteins and analytes in 2007,^91^ and although it has the benefit of not requiring protein immobilization, the instrumentation is not commercially available and so take-up in drug discovery settings has been limited.^92^

### 3.1 Surface Plasmon Resonance (SPR)

SPR is the most commonly used biosensor to monitor protein–ligand interactions and determine kinetic parameters.^40^ In SPR, light passes through a prism and is reflected off a sensor chip surface (usually gold) into a detector under total internal reflection conditions. At a specific angle, known as the resonant angle, electrons in the gold layer become excited by absorbing energy from the incident photons, generating surface plasmons. This results in a loss of photons through absorbance therefore a reduction in the intensity of the reflected light at the resonant angle (Figure 13a).^78^ The resonant angle is altered by differences in the refractive index of the biolayer which is related to the mass bound at the surface.

**Figure 13:**
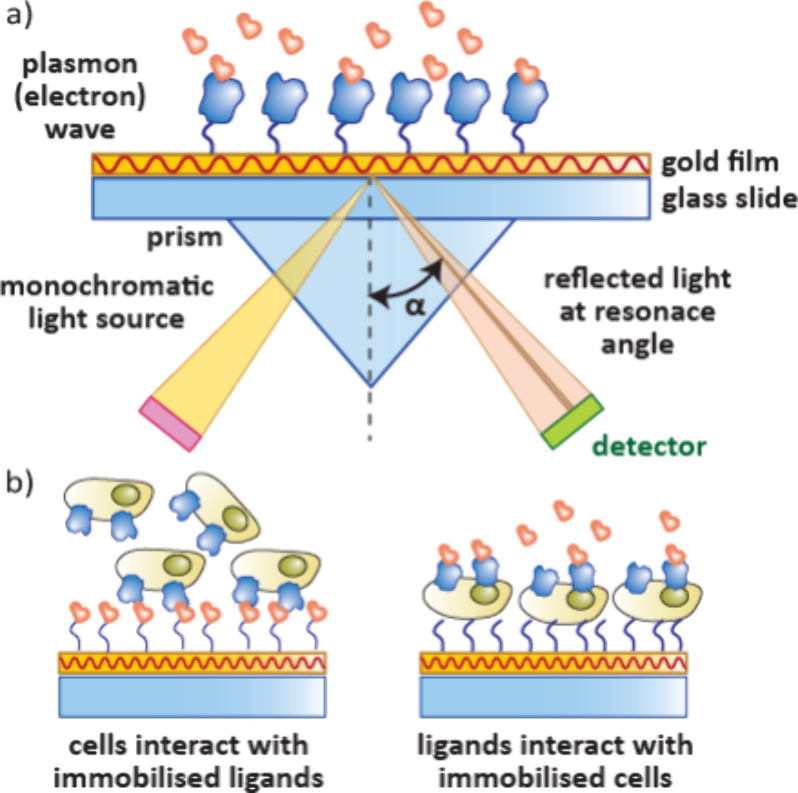
a) Setup of an SPR experiment. Monochromatic light passes through a prism onto a gold surface and is reflected off the surface into a detector. At the resonant angle there is a reduction in intensity of reflected light. The resonant angle is sensitive to refractive index changes, which is influenced by protein–analyte interactions on the gold surface. b) SPR with intact cells can be used to detect interactions with surface bound proteins and their analytes.

SPR has been used to screen and validate compounds.^93^ It is also sensitive enough to enable its use in FBDD.^94^ It can additionally be used to analyze the kinetics of ternary complex formation for PROTACs.^95^ SPR has been used to detect interactions with surfaced bound proteins and their analytes with either immobilized small molecules or immobilized cells (Figure 13b). To date these cellular techniques have largely been limited to medical diagnostics but could provide a useful tool for the evaluation of analytes of membrane-bound proteins.^96^

### 3.2 Grating Coupled Interferometry (GCI)

GCI is another optical biosensor that monitors changes in the refractive index of a biolayer. In GCI, a laser is coupled into a waveguide using a grating. The light then passes through the waveguide with the biolayer surface, which causes a phase shift. A reference beam is then also coupled into the waveguide which results in interference. The combined waves are then decoupled from the waveguide using a third grating and the interferogram is measured at a detector (Figure 14). Unlike SPR which monitors a localized area, GCI samples the entire surface so has enhanced sensitivity.^97^ Scientists at Crieoptix (now Malvern) have improved the throughput of GCI kinetic experiments using Repeated Analyte Pulses of Increasing Duration (waveRAPID). In this protocol, increasing concentrations of analyte are achieved by increasing the number of injections as opposed to creating samples of different concentrations.^98^

**Figure 14:**
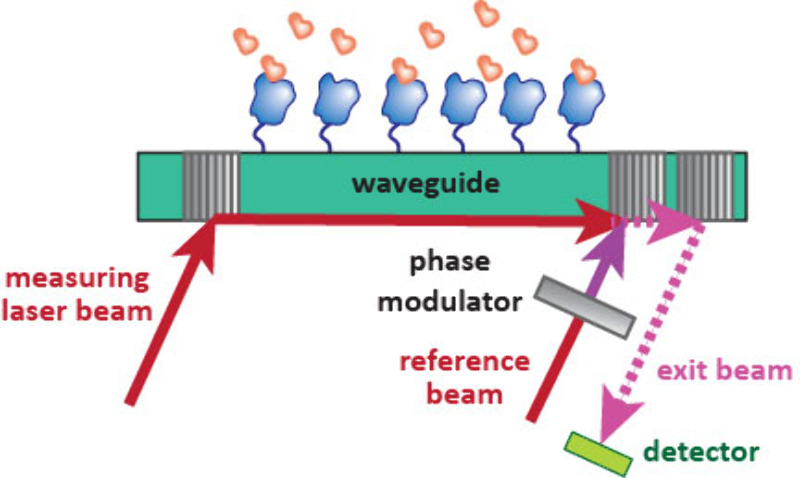
Setup of a GCI experiment. A grating couples a laser beam through a waveguide. The biolayer on the waveguide causes a variable phase shift due to changes in refractive index. A reference beam is added which interferes with the sample beam and the output is coupled out of the waveguide with another grating, giving a time-resolved phase shift.

Like SPR, GCI is suitable for library screening, hit validation as well as fragment screening,^98^ though has yet to be used in cell-based applications.

### 3.3 Biolayer Interferometry (BLI)

BLI is another label-free optical biosensor. A key difference from other sensors is that instead of using microfluidics systems, the biosensor surface for BLI is at the end of an optical fiber tip which is immersed into various analyte solutions.^99^ BLI works by shining white light to the end of an optical fiber tip which consists of an optical layer and the biolayer containing immobilized protein (Figure 15). Light is reflected and if analyte is bound, the wavelength of the reflected beam will change. As no microfluidics are used, BLI can be applied to complex or viscous solutions such as plant and microbial extracts and it is more tolerant to organic solvents such as DMSO than SPR.^99^ BLI is amenable to screening medium-sized compound libraries,^100^ and to analyze ternary complex formation in PROTACS.^36^

**Figure 15:**
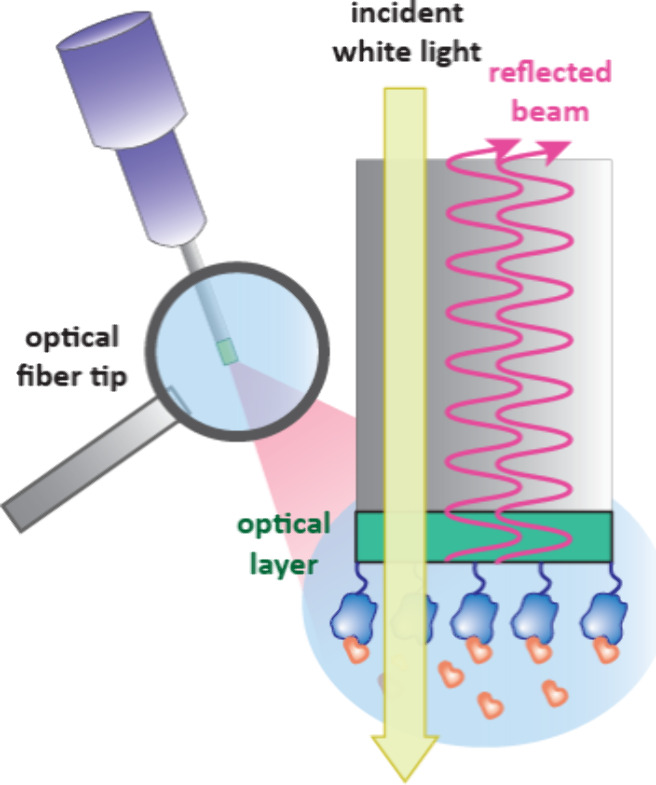
Setup of a BLI experiment. Light is passed through an optical fiber containing an optical layer and biolayer at the tip. The wavelength of the reflected beam is influenced by the thickness of the biolayer. The tip is dipped into various solutions of analyte.

### 3.4 Electrically Switchable Nanolevers (SwitchSense)^101^

SwitchSense was first developed in 2013 to determine the shape and size of proteins.^102^ In this technique, double stranded DNA is immobilized onto a gold chip which is attracted and repelled to the surface by means of applying an alternating potential (Figure 16). One strand of the ds-DNA is labeled with a fluorescent tag that is quenched when it is in proximity to the gold surface, a fluorescence detector can therefore determine the orientation of the DNA strands in real time. For drug-target engagement applications, the complementary strand has the protein target attached, which can interact with ligands. Ligand binding can be detected as a change in the switching dynamics of the DNA strand.

**Figure 16:**
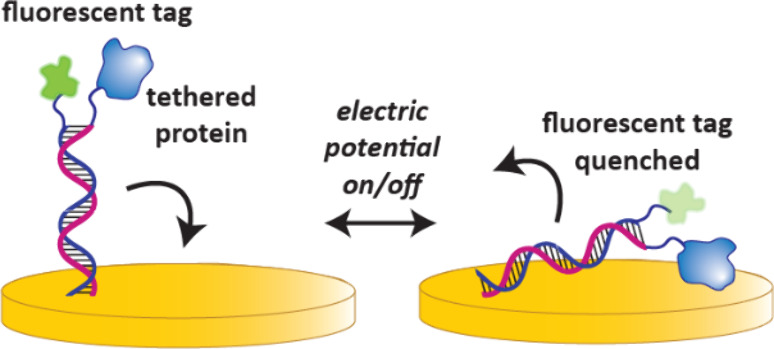
Theory of a SwitchSense experiment. An alternating electric potential attracts and repels ds-DNA to a gold surface, switching on and off fluorescence. One strand of the ds-DNA contains the switchable fluorophore, whereas the other can be tethered to a protein of interest. Binding of a ligand to this protein can be detected as changes in the switching dynamics of the DNA.

### 3.5 Magnetic Force Spectroscopy (MFS)

The life sciences tools company Depixus has developed the MAGNA One instrument which enables protein–ligand and protein–protein interactions to be analyzed using MFS (Figure 17).^38^ It achieves this by monitoring the change in vertical height of a magnetic microbead tethered via a DNA strand to a surface when applying a magnetic force. The protein and ligand are tethered to distal positions of the DNA strand using complementary DNA sequence tags. When a low magnetic force is applied, the protein and ligand will be bound together, and the bead will stay in a lower position. However, when a stronger force is applied the protein and ligand will separate and the bead will move vertically toward the magnet. The time it takes for the bead to reach full height is indicative of the binding strength between the protein and the ligand.

**Figure 17:**
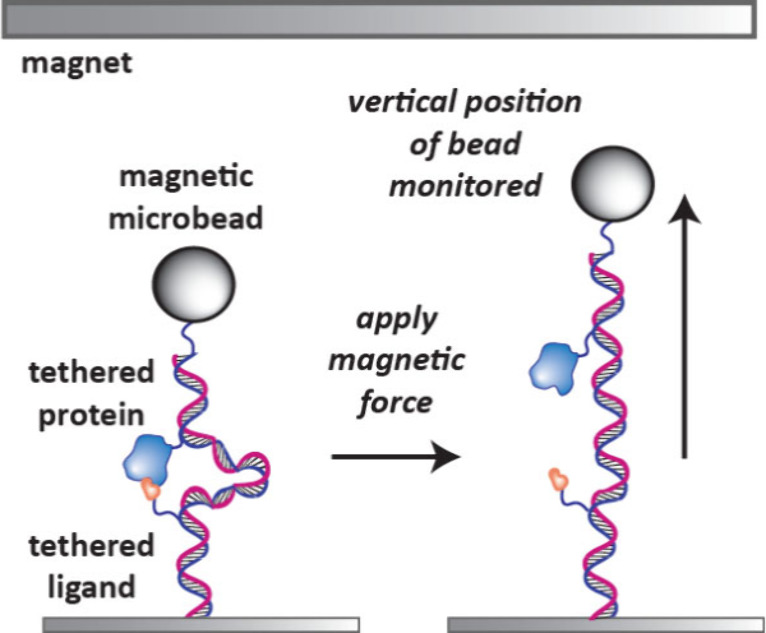
Magnetic force spectroscopy to analyze protein–ligand interactions. The protein and ligand are bound to a tethered DNA strand with a magnetic microbead. As a magnetic force is applied the bead will be attracted to the magnet, causing the protein and ligand to separate. Delays in the time for the bead to reach the full hight upon applying this increased force is related to the strength of the protein–ligand interaction.

If two proteins are bound to the DNA strand, this technique can be used to monitor protein–protein interactions and develop molecular glues and PPI inhibitors.^38^

### 3.6 Total Internal Reflection Fluorescence Microscopy (TIRF)

Total internal reflection occurs when light is reflected at a boundary into a medium of lower refractive index beyond a critical angle. This creates an electromagnetic evanescent field which decays a short distance from the surface. This electromagnetic field can be used to excite nearby fluorophores which are visualized in a microscope. TIRF microscopy has been used to monitor interactions of membrane bound proteins and ligands using a technique called dynamic Inhibition-in-Solution Assays (dISA).^103,104^ This is achieved by imbedding both a protein of interest and the fluorophore into a vesicle. When the protein binds to a tool compound on the biosensor surface, the fluorophore is excited by the evanescent wave (Figure 18). Presence of a competitively binding ligand results in less interactions between the protein and the surface and therefore less fluorescence. The number of binding events over time with and without ligand are monitored.

**Figure 18:**
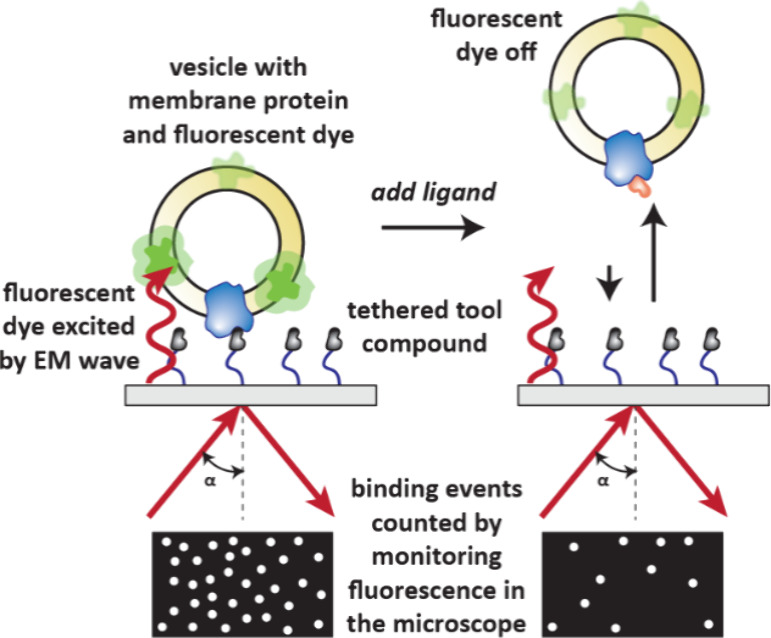
Dynamic Inhibition-in-Solution Assay (dISA) using TIRF Microscopy. A vesicle containing the membrane protein of interest and a fluorescent dye interacts with a tool compound on the surface of a TIRF biosensor. This brings the dye in proximity to the surface where it is excited by the effervescent wave and fluoresces. Addition of a competitive ligand reduces the interactions with the protein and tool compound and so fluorescence viewed in the microscope is reduced.

## 4 Mass Spectroscopy (MS)

Mass spectroscopy is a powerful tool to observe drug-target engagement. A MS instrument consists of an ionization source, mass analyzer and detector. The ionization source both ionises molecules and transfers them to the gas phase, the ions are then separated by mass to charge ratio (*m*/*z*) before being detected. This results in a mass spectrum of *m*/*z* versus the abundance or intensity of that ion.^105^ Many ionization techniques and mass analysers exist providing mass data with variable sensitivity and throughput. Importantly, mass spectroscopy is label free and so is largely unaffected by potential interferences present in other assays.^21^ The mass of whole proteins can be observed with Native MS. AS-MS analyses the mass spectra of ligands following separation based on their affinity to a protein of interest; it encompasses many techniques and is widely used to screen compounds and fragments. HDX-MS methods are low throughput but used for further characterization of protein–ligand complexes to provide information on the ligand binding site.

### 4.1 Native-MS

Native MS is used for studying intact, folded proteins, including their noncovalent complexes with small molecule ligands.^106^ It is usually carried out with electrospray ionization (ESI),^107^ and can determine if binding is present, stoichiometry of binding, *K*_D_ and estimate the enthalpic component of binding.^20^ Native MS has the advantage that there is no need for labeling or cross-linking and only picomoles of material are needed. However, there is a question as to whether ESI can preserve the solution structure of assemblies into the gas phase, as proteins can unfold rapidly.

In Native MS, a volatile solution of protein is made by buffer exchange using gel filtration, Size Exclusion Chromatography (SEC) or dialysis. A ligand can then be added before analyzing the sample by ESI-MS (Figure 19a). This will give a mass spectrum with all the different charges of both protein and protein–ligand complex (Figure 19b), and deconvolution gives a ratio of free protein and protein–ligand complex (Figure 19c).^106^

**Figure 19:**
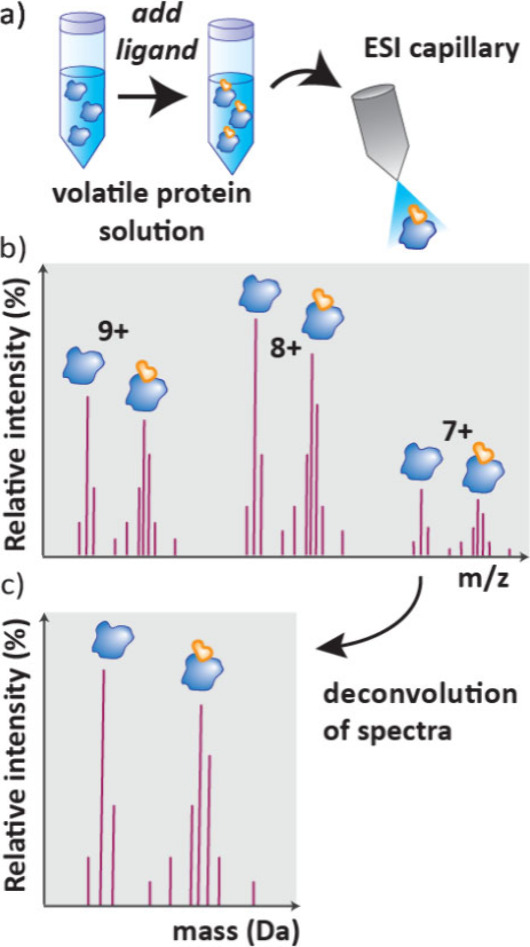
Workflow and results of a Native MS experiment. a) Volatile protein–ligand solutions are prepared and analyzed by ESI-MS. b) A mass spectrum of all the possible charges is generated which c) is deconvoluted to give the ratio of protein:protein–ligand complex.

Native MS has the advantage that many compounds can be screened per well, and it has been successfully applied in library screening^106^ including of fragments,^108^ enabled by improved automation.^109^ Intact protein MS is also used in covalent drug discovery to identify both covalent fragments and drug-like molecules.^19^

Analysis of protein–ligand complexes was enabled in *Escherichia coli* cell lysates in 2017.^110^ Success has also recently been achieved in running native MS experiments in human erythrocytes, meaning that cellular native MS experiments to observe protein–ligand interactions in cells may be possible in the future.^111^

### 4.2 Affinity-Based Selection (AS-MS)

AS-MS is the most common biophysical technique used in primary screening. It analyses the mass spectra of small molecule ligands following their separation based on their affinity to a protein of interest. There are numerous methods of discriminating ligands based on their affinity (Figure 20).^112–114^ In AS-MS, SEC, PUF and MagMAS are most widely used, though FAC, ED and SAMDI have also been employed to separate ligands based on their affinity to a protein of interest. AS-MS has the benefit that more than one compound can be tested at any one time. It also does not require radioisotopes or chromophores and is not subject to fluorescent interference.^115^ Following initial development in 1997,^116^ AS-MS has gone on to be used in automated screening cascades,^117^ enabled by more sensitive acoustic mist mass spectroscopy.^118^

**Figure 20:**
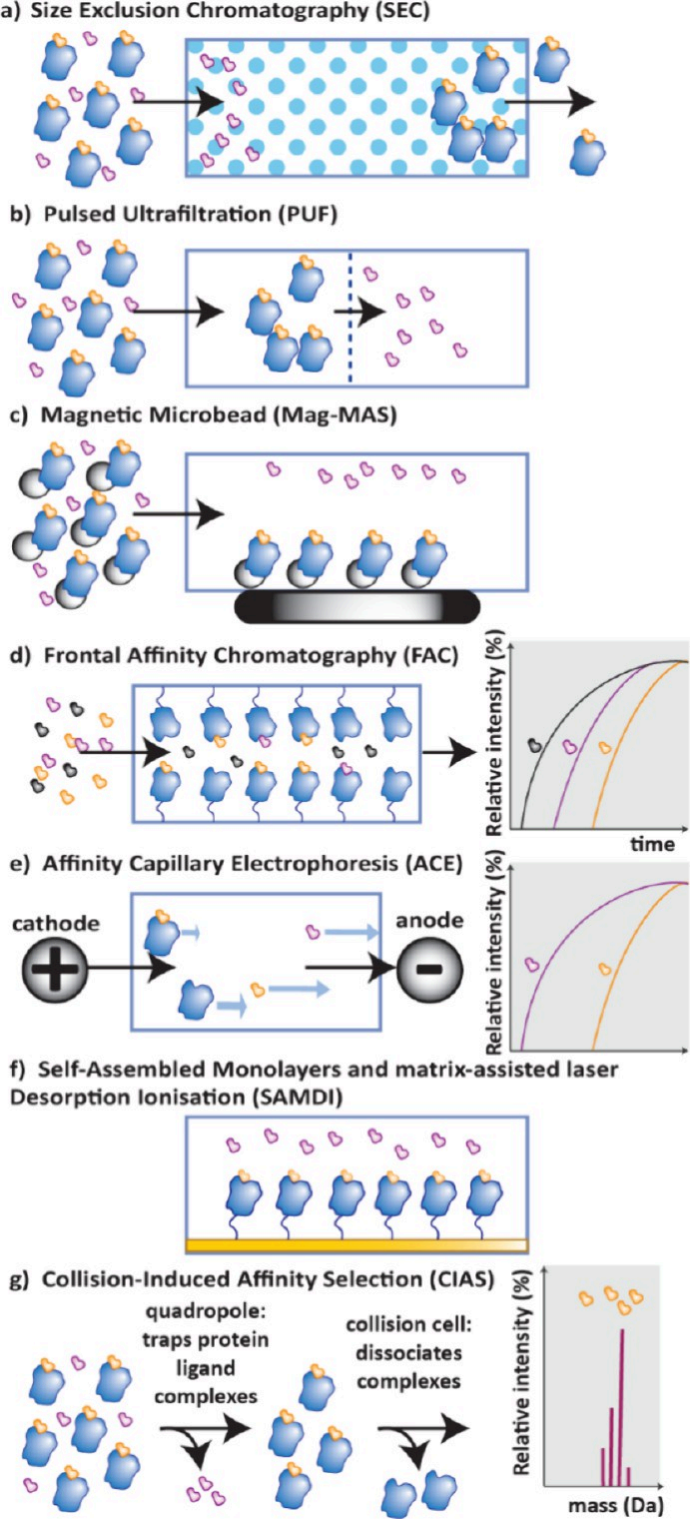
Separation techniques to discriminate ligands by binding affinity in AS-MS, strongly binding ligands are shown in orange and weak binders in purple. a) SEC: free nonbinding ligands and protein–ligand complexes are separated by size. b) PUF: unbound ligands are separated from complexes by pulsed filtration through a membrane. c) Mag-MS: The protein is bound to magnetic beads which enables washing away of unbound ligands following application of a magnet. d) FAC: ligands are passed through a column of immobilized protein; a void marker (shown in black) followed by weakest binders will elute first. e) ACE: free protein, protein–ligand complexes and free ligands move across an electrophoresis capillary at different velocities dependent on their size and charge. f) SAMDI: protein–ligand complexes are immobilized on a gold surface and analyzed by MALDI. g) CIAS: separation of bound and unbound ligands is achieved in a quadrupole after ionization by ESI. The protein and ligands are dissociated in a collision cell and then the masses of the small molecule ligands are detected.

#### 4.2.1 Size Exclusion Chromatography (SEC-MS)

SEC columns contain spherical beads that do not absorb compounds but allow the separation of molecules and complexes by size, by how easily they diffuse into pores in the column (Figure 20a). Large molecules such as proteins and their complexes do not fit in the pores, so they elute first. Miniaturization of SEC columns has enabled the coupling with MS for screening of compounds against biological targets.^119^ After passing through the SEC column, the protein–ligand complexes are denatured to release the bound ligands using liquid chromatography (LC) before being analyzed by MS. This technique was first developed to enable the rapid screening of ligands by NeoGenesis in 2004, which they named the Automated Ligand Identification System (ALIS).^120^ meanwhile Novartis developed the SpeedScreen platform which uses microtiter-plate SEC instead of continuous flow.^121^

#### 4.2.2 Pulsed Ultrafiltration (PUF-MS)

In PUF-MS, an ultrafiltration membrane enables unbound ligands to pass through, leaving those bound to a protein target behind (Figure 20b). The bound ligands are then released by denaturing the complex using an organic solvent or pH change before being analyzed by MS or LCMS.^122^

#### 4.2.3 Magnetic Microbead (MagMAS-MS)

By tethering a protein onto a magnetic microbead, protein–ligand complexes can be separated from unbound ligands by applying a magnet and washing away unbound species (Figure 20c).^123^ Similar to other methods, the bound ligands are then released by denaturing the protein before they are analyzed by LCMS. This technique has been shown to be suitable for FBDD with a GPCR target.^124^

#### 4.2.4 Frontal Affinity Chromatography (FAC-MS)

In contrast to “catch and release” methods, FAC enables the evaluation of the binding strengths according to the delay of analyte molecules passing through an affinity column of immobilized protein (Figure 20d).^125^ The companies SARomics and RG Discovery developed a FAC-MS-based fragment screening platform called Weak Affinity Chromatography (WAC).^126^ This technique has very recently been further miniaturized and applied to fragment screening for membrane proteins.^127^

#### 4.2.5 Affinity Capillary Electrophoresis (ACE-MS)

ACE-MS was first used in 1996 for the screening of combinatorial libraries.^128^ It relies on changes in electrophoretic mobility, how fast a molecule moves across an electrophoresis capillary, which is dependent on charge and size and is different for free ligands and their complexes (Figure 20e).^129^ Similar to FAC-MAS, the delay time of ligands passing through the electrophoresis capillary gives an indication of the binding strength, with weak binders eluting faster.

#### 4.2.6 Self-Assembled Monolayer Desorption Ionization (SAMDI)

SAMDI combines the use of self-assembled monolayers on gold with MALDI-MS.^130,131^ It has largely been used for enzyme activity assays, but in 2017 was first used to analyze noncovalent ligands.^132^ In this method, compounds are incubated with biotinylated protein to enable complex formation, this solution is then applied to neutravidin coated SAMDI plates to capture protein (Figure 20f). Plates are then washed to remove any nonbinding compounds and small-molecule MALDI finds the masses of the bound compounds.

#### 4.2.7 Collision-Induced Affinity Selection Mass Spectrometry (CIAS-MS)

CIAS-MS was first reported by Liu and Quinn in 2022.^133^ Other AS-MS techniques require separation of bound and unbound ligands prior to MS whereas CIAS-MS allows the discrimination and analysis of binding and nonbinding ligands all inside the MS instrument. In CIAS-MS, solutions of proteins with ligands are injected into an ESI source for ionization then transferred to a quadrupole which traps complexes while excluding any unbound small molecules. The protein–ligand complexes are then subjected to collision induced dissociation and the mass of the small molecule ligands are analyzed (Figure 20g). Notably CIAS-MS can be used to analyze interactions with ligands in complex mixtures such as plant extracts.^133^

### 4.3 Hydrogen–Deuterium Exchange (HDX-MS)

In HDX-MS, the amount of deuteration of accessible and exchangeable protons on the surface of a protein when subjected to a deuterated buffer solution is analyzed. This provides information about the binding site, as the amount of hydrogen–deuterium exchange on amino acid residues involved in ligand binding will be different than those elsewhere on the protein surface.^20^ HDX-MS is particularly useful to provide structural information about proteins that have proven hard to crystallize.^134^ There are three possible workflows of HDX-MS: bottom-up, top-down or middle-down. All methods involve incubating the protein in a D_2_O buffer, causing hydrogen–deuterium exchange of the labile surface NH, OH and SH groups, the reaction is then quenched by lowering the pH.^135^

The most used technique is bottom-up HDX-MS in which after hydrogen–deuterium exchange, the protein is denatured, cleaved into smaller peptides with a protease and the resultant peptide fragments analyzed by LCMS (Figure 21a). These spectra are compared to the experiment conducted in a nondeuterated buffer to determine the percentage deuteration for each peptide fragment (Figure 21b). The percentage deuteration of each fragment with and without the presence of ligand can help identify a possible binding site on the protein (Figure 21c). The optimal digestion of the enzyme would result in overlapping residues (normally 5–30 amino acids long) which enables the determination of which peptides (not only which fragments) have been deuterated. Notably, some reprotonation of the exchangeable sites may occur on the LC column, called back-exchange. This is why only short LC methods of less than 15 min are generally used despite giving poor separation of the peptide fragments.^134^

**Figure 21:**
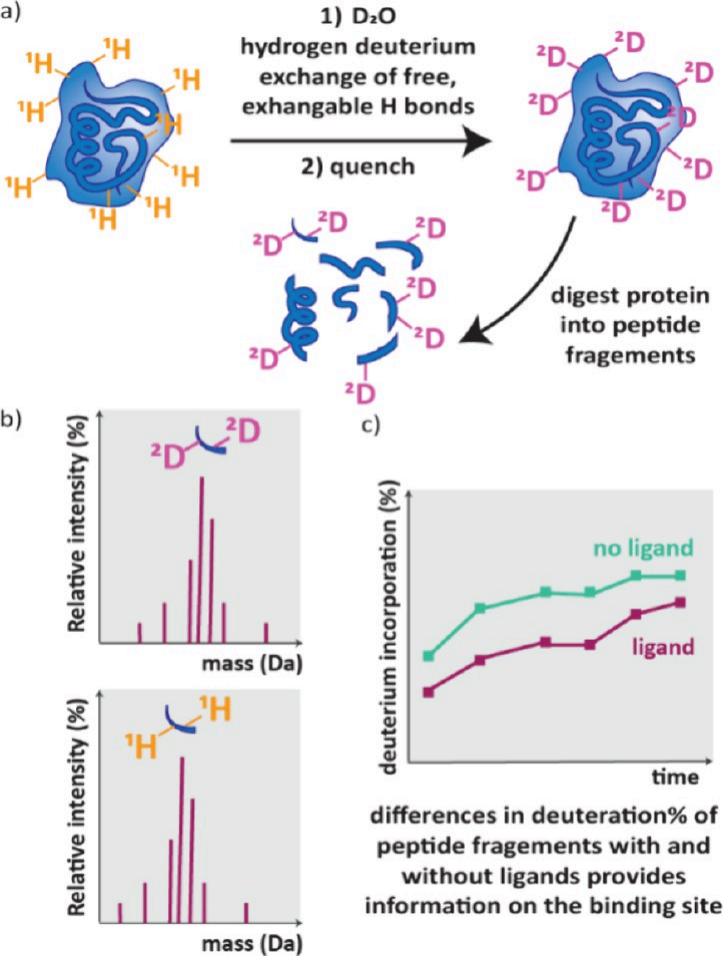
Bottom-up HDX-MS. a) Surface exchangeable protons are exposed to a deuterated buffer leading to exchange before the reaction is quenched and the protein digested into peptide fragments. b) The fragments are analyzed by LCMS and compared to their non deuterated counterparts to determine the percentage deuteration. c) Comparison of the deuteration of the peptide fragments of free protein with ligand bound protein can provide information about the binding site.

Recently, coupling HDX-MS with subzero temperature ultraperformance liquid chromatography separation (UPLC) has made improvements in throughput and enabled analysis in *E. coli* cell lysate.^136^ HDX-MS in live *E. coli* cells has also been recently reported to examine the structure of BtuB protein *in vivo*,^137^ which provides proof of concept for future development of this methodology to investigate protein–ligand interactions in live cells.

Lesser used forms of HDX-MS include top down and middle down approaches. Top-down HDX-MS is like native MS (see section 5.1) in that the intact protein is analyzed by ESI-MS. It is used to observe global changes on the protein. Middle down HDX-MS is a hybrid of bottom-up and top-down methods where enzymes are used to digest the protein, but to longer peptide fragments that are analyzed by ESI.^134^

## 5 Nuclear Magnetic Resonance (NMR)

There are two NMR spectroscopy techniques which can be used to observe drug-target interactions. In ligand-observed NMR (LO-NMR), NMR parameters such as integral values and relaxation rates of a ligand are observed in the presence of a protein. Protein-observed NMR (PO-NMR), examines the NMR spectra of the proteins themselves, usually using isotopic labels to simplify the spectra. Both methods require relatively large amounts of highly pure, stable protein, though improved electronics, higher magnetic field strengths and cryoprobes have increased the sensitivity of NMR more than 10-fold over the past 10 years, reducing the amount of sample required and acquisition time.^20^ NMR assays are well suited for fragment screening as they can work at high concentrations of ligand, which is required to detect weak inhibitors. Use of NMR based assays can help confirm results from spectrophotometric assays where overlapping UV–vis absorption profiles may complicate analysis.^138^

### 5.1 Ligand-Observed NMR (LO-NMR)

In LO-NMR, the ^1^H or ^19^F NMR spectra of small molecules are analyzed in the presence of the protein of interest. Within this category, several methods have been developed which observe changes in various NMR parameters of ligands as they bind with proteins. These are broadly split into relaxation-based methods and Nuclear Overhauser Effect (NOE) based methods. The throughput of LO-NMR experiments can be improved by using cocktails of ligands.^139^ As weak binders can be readily detected with LO-NMR, it is well suited to FBDD.^140^ LO-NMR has been to screen compounds for challenging targets such as PPIs.^141^ A limitation in using LO-NMR experiments is the difficulty in identifying strong binding ligands as well as the need for highly soluble compounds, though this can be combated by using competition experiments.^142^ The need for high concentrations of ligand can also lead to compound aggregation which should be considered when interpreting results, particularly with less sensitive NOE-based methods.

#### 5.1.1 Relaxation Based Methods

Binding to a protein affects the transverse relaxation rate (*R*_2_) of ligands. LO-NMR experiments that exploit this difference in transverse relaxation rate between bound and unbound ligands can be easily set up to qualitatively determine if binding is occurring. Due to the ubiquity of protons in organic compounds and their high sensitivity for NMR analysis, ^1^H NMR spectroscopy is most frequently used. In general, although various pulse sequences are used and various parameters are measured, addition of protein causes a decrease in the intensity of the signals of the ligand (Figure 22). If a competitor molecule is then added, the signals would increase as the ligand is displaced from the protein.

**Figure 22:**
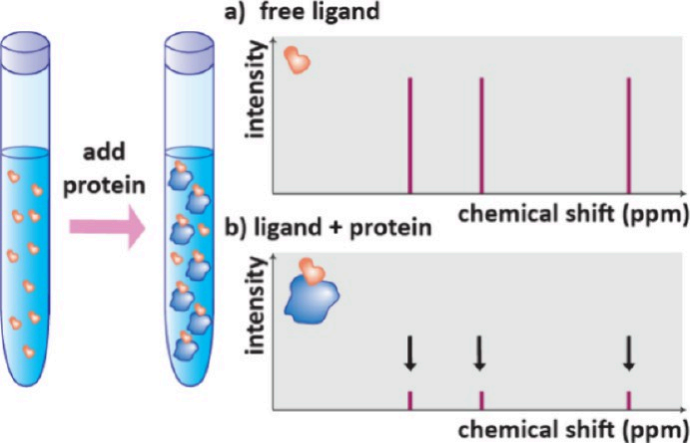
^1^H NMR spectra during LO-NMR spectroscopy using transverse relaxation-based methods. a) ^1^H NMR spectra of free ligand. b) When combined with protein, the signals for the ligand protons are reduced if binding occurs.

The line width of the ligand signal is proportional to the transverse relaxation rate (*R*_2_). Line width changes can be translated into changes in peak height (therefore easier to observe) by using a Carr–Purcell–Meiboom–Gill (CPMG) pulse program.^143^ For spectra acquired with this pulse program, the intensity (peak height) of NMR signals is proportional to the ligand’s transverse relaxation rates (*R*_2_) and delay time settings in the pulse program. With a longer delay time, NMR signals of a molecule with a fast relaxation rate, such as ligands that interact with the protein, could disappear completely, providing evidence of target engagement.^144^ Recently quantitative LO-NMR has been developed using transverse relaxation rate (*R*_2_) as the measurable parameter.^33^ This method is well suited to FBDD for a *K*_D_ range of 10 μM to 1 mM.

#### 5.1.2 Water-Ligand Observed via Gradient Spectroscopy (WaterLOGSY)

WaterLOGSY relies on the transfer of proton magnetization from excited water molecules to ligands from either (A) direct transfer (positive NOE) or (B) via initial transfer to protons at a protein surface which causes a negative NOE (Figure 23).^145^ The difference in transfer behavior of the bound and unbound ligands is related to the tumbling rates of the molecules in solution, which is fast for unbound molecules and slow for bound molecules.

**Figure 23:**
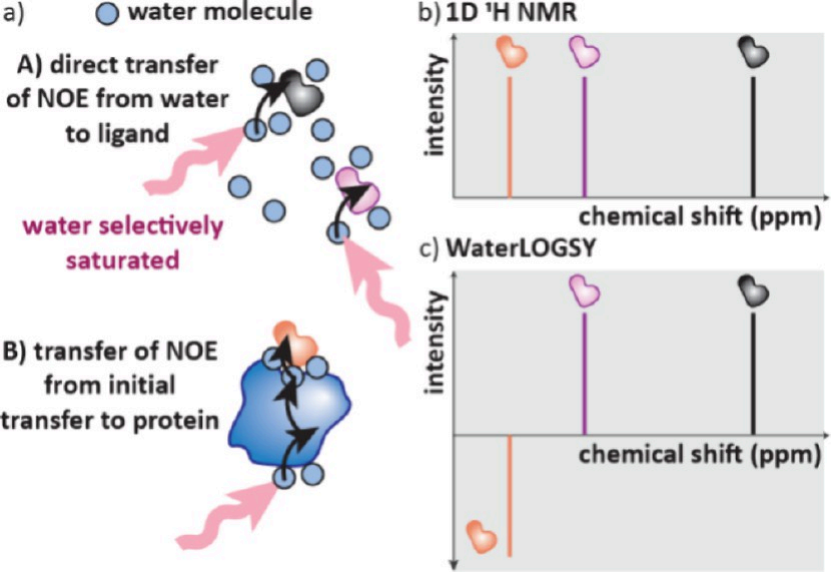
a) In a WaterLOGSY experiment, water molecules are selectively excited. NOE can occur A) directly from excited water molecules to a ligand or B) via transfer to protons at a protein surface. b) A 1D ^1^H NMR spectrum of a mixture of ligands is compared to the WaterLOGSY NMR spectra c) ligands that bind will have a negative NOE.

WaterLOGSY has been reported to determine the *K*_D_ of compounds and fragments.^146^ Solvent Accessibility, Ligand binding, and Mapping of ligand Orientation by NMR spectroscopy (SALMON) is a modified water-LOGSY experiment to further elaborate the binding modes of ligands.^147^

#### 5.1.3 Saturation Transfer Difference (STD-NMR)

STD-NMR spectroscopy is based on the transfer of saturation from a protein to the bound ligand. This is achieved by selectively saturating the entire protein’s resonances by Gaussian soft pulses (Figure 24). If the ligand is bound to the protein, the saturation will be transferred to the ligand by ^1^H–^1^H cross relaxation. Notably, only the ^1^H NMR signals of protons directly involved in binding will receive high degrees of saturation,^148^ so this technique can provide information about parts of the molecule that are involved in key binding interactions.

**Figure 24:**
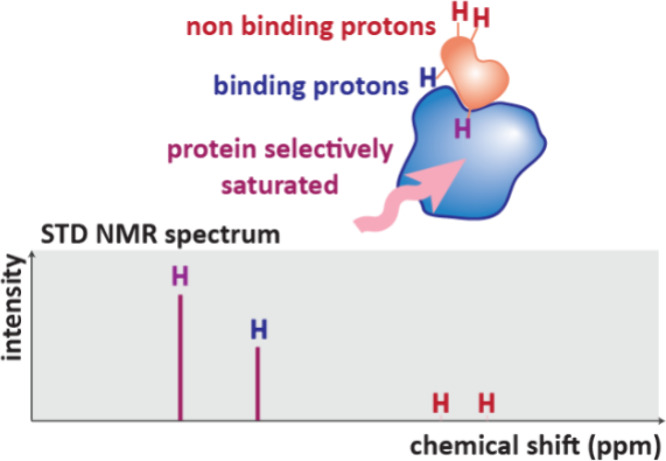
STD NMR spectrum shows the changes in intensities for ligand protons between the free ligand and after the protein has been saturated by Gaussian soft pulses. The largest signals show the protons most involved in protein–ligand binding.

#### 5.1.4 Interligand NOE for Pharmacophore Mapping (INPHARMA)

The INPHARMA technique is NOE based, but unlike water-LOGSY and STD, which observe NOEs between water and the protein respectively, NOEs between two ligands are investigated. In INPHARMA, a NOESY spectra of two ligands in the presence of the protein is recorded. If they bind in the same site, an NOE signal will be observed for the binding protons, originating from transfer of magnetization between one ligand to the protein, then from the protein to the second ligand, as long as the rate of exchange of the ligands is fast.^149^

#### 5.1.5 Target Immobilised NMR Screening (TINS)

TINS is the only LO-NMR method where an immobilized protein is used. This means ligands can be washed away and the protein can be used again for further experiments, reducing protein consumption.^139,150^ TINS has enabled the screening of 2000 compounds without diminishing the surface and is well suited for FBDD.

#### 5.1.6 Spin Labels Attached to Protein Side Chains as a Tool to Identify Interacting Compounds (SLAPSTIC)

SLAPSTIC was first developed in 2002 to improve sensitivity and decrease protein consumption in LO-NMR.^151^ It uses organic nitroxide radicals as spin labels which reduce signals of proximal ligands by paramagnetic relaxation enhancement. However, this method relies on selective labeling of target residues on the protein or ligand.^139^

#### 5.1.7 Fluorine Chemical Shift Anisotropy Exchange (FAXS)

The fluorine nucleus undergoes fast relaxation leading to increased sensitivity in NMR spectroscopy. It is also highly responsive to changes in environment and is generally absent in biological molecules, so the use of ^19^F NMR spectroscopy to analyze protein–ligand binding is a very powerful tool in drug discovery.^152^ For LO-NMR, fluorine chemical shift anisotropy and exchange for screening (FAXS)^153^ can be used. Like experiments using ^1^H NMR spectroscopy, FAXS observes the decrease in signal of a ligand upon binding to a protein (Figure 25a) or increase in signal of a displaced reporter molecule in competition experiments (Figure 25b). Chemical shift-anisotropy-based affinity ranking (CSAR) has recently emerged to rank fluorinated ligands without the need for titrations or isotopically labeled protein by providing relaxation data that is directly proportional to binding affinity.^154^

**Figure 25:**
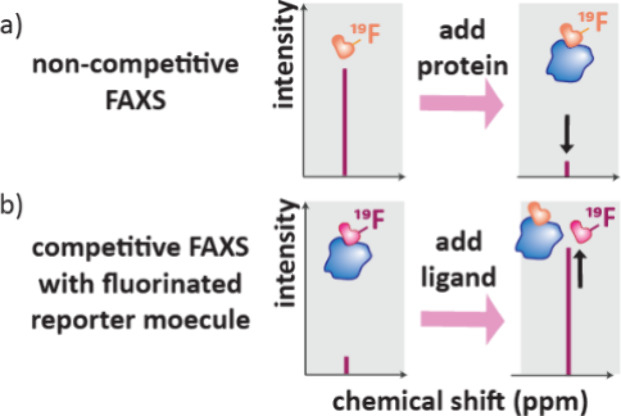
^19^F NMR spectra for FAXS NMR spectroscopy. a) Intensity of the signal for fluorinated ligands will be reduced on addition of protein. b) Competitive FAXS: addition of a ligand can displace a fluorinated reporter molecule.

The lack of native ^19^F means that ^19^F NMR experiments are particularly useful for in-cell assays. The displacement of fluorinated reporter molecules bound to target proteins in cells has recently been used to determine *K*_D_ values for various Hsp90α ligands in living cells.^155^

#### 5.1.8 Photochemically Induced Dynamic Nuclear Polarization (Photo-CIDNP)

Hyperpolarisation can increase the sensitivity of LO-NMR spectroscopy for use in screening small molecules against biological targets, so that lower sample concentrations can be used.^156^ in photo-CIDNP, protein–ligand mixtures are irradiated in the presence of a photosensitizer and a radical pair is created between the photosensitizer and the ligand. Polarization of protein-bound ligands through this radical pair mechanism is quenched and so this method can be used to quantify binding. Not all possible ligands can be hyperpolarised using this technique, however, with an estimate of 25–30% of biologically active molecules being amenable, though libraries of suitable fragments are in development.^156^ Displacement of suitable polarizable tool compounds can also be investigated to enable screening of more diverse compound libraries.

### 5.2 Protein-Observed NMR (PO-NMR)

PO-NMR can be used to determine protein dynamics, ligand affinities and provide information on the binding site.^4^ Due to the large size of proteins, their NMR spectra are complex. Because of this, many PO-NMR techniques use isotopically labeled proteins, though notably there are still considerable costs associated with their production. PO-NMR is in general more labor intensive to interpret the data than LO methods, but it provides additional information about protein structure alongside binding confirmation.

#### 5.2.1 ^1^H–^15^N NMR Heteronuclear Single Quantum Correlation (^1^H–^15^N HSQC)

The most widely used PO-NMR experiments are 2D ^1^H–^15^N NMR heteronuclear single quantum correlation (HSQC) with ^15^N labeled residues.^139^ Chemical shift perturbation (CSP) using ^15^N labeled protein is the gold standard in determination of *K*_D_ values with NMR.^33,157^ It follows the change in chemical shift(s) of a protein when a ligand is added (Figure 26). If the position of labeling is known and the structure of the protein solved, CSP experiments can be used to determine the location of a binding site, as the peaks that move the most are the most likely to map to this area of the protein.^158^

**Figure 26:**
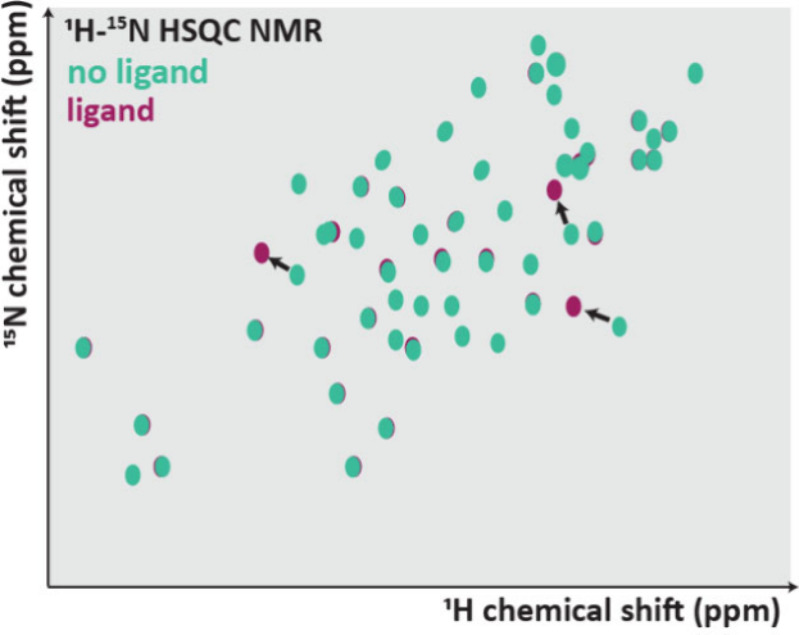
Most PO-NMR experiments are based on 2D ^1^H–^15^N NMR HSQC using proteins with ^15^N labeled amino acid residues. Analysis of changes in chemical shift with and without ligands can be used to identify binding affinities and locate the binding site. The signals for the residues involved in ligand binding will be shifted the most.

PO-NMR has mostly been limited to proteins of less than 40 kDa, however selective labeling of specific amino acid types means that this limit can now be exceeded.^20^ Improvements in methods have also increased the throughput of PO-NMR experiments for use in screening of small molecules and fragments.^20^

Band-Selective Optimized Flip Angle Short Transient Heteronuclear Multiple Quantum Coherence (SOFAST-HMQC) is a pulse sequence that was first developed in 2005 to reduce the acquisition time of ^1^H–^15^N NMR correlation spectra.^159^ It enables the increased throughput of PO-NMR experiments and is commonly used in compound screening.

Transverse Relaxation-Optimized Spectroscopy (TROSY) was first developed in 1997, it is a pulse program that exploits the cancellation of dipolar coupling and chemical shift anisotropy (CSA) between dipolar couplings. It enables the recording of sharp peaks in the ^1^H–^15^N NMR spectra of proteins and is particularly useful for large proteins (>30 kDa).^160^ Methyl-TROSY has also been developed which uses ^13^C labeling of methyl groups on proteins.^161^

#### 5.2.2 1D ^1^H-Aliph NMR Spectroscopy

Perhaps the simplest PO-NMR spectroscopy method is to observe the signals in the aliphatic region (below 0.7 ppm) of the ^1^H NMR spectra of a protein in the presence and absence of ligand (Figure 27). This region contains signals belonging to methyl groups on the protein that are proximal to aromatic side chains. CSP observations in this region can be used to estimate dissociation constants of titrated ligands.^162^ In order to use this method, a signal in this region must undergo meaningful change upon ligand binding, so it can only be used in highly specific cases.

**Figure 27:**
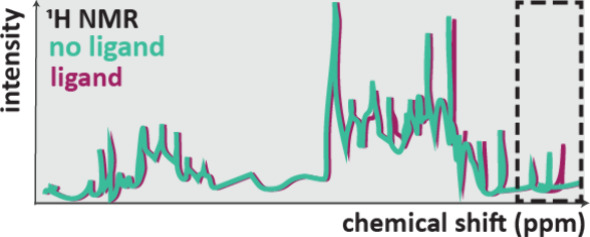
In 1D ^1^H NMR Spectroscopy of a protein, changes in chemical shift in the simpler low aliphatic region can be used to provide information about ligand binding.

#### 5.2.3 Cellular PO-NMR Assays

Although signal-to-noise ratio remains a great challenge, innovations over the last 20 years has shown that standard PO-NMR spectroscopy techniques such as ^1^H–^15^N HSQC can be utilized within living cellular environments.^163^ Although the field is still in development, a preliminary study has shown that ligand binding curves can be obtained in cells against a human carbonic anhydrase (CA2) with known inhibitors.^164^

STructural INTeractions by in-cell NMR (STINT-NMR) is a ^1^H–^15^N HSQC method which was first developed in 2006 for in cell analysis of protein–protein interactions.^165^ It has since been applied by various groups for screening of small molecules that mediate PPIs.^166,167^

#### 5.2.4 ^19^F Fluorine Protein Observed NMR (PrOF)

Proteins can be labeled with ^19^F atoms which is particularly useful as fluorine can be used as an isosteric replacement for a hydrogen atom.^168^ In PrOF, chemical shift perturbations of the ^19^F signal on a labeled protein enables the determination of the degree of ligand binding (Figure 28). These experiments also enable observations of nonspecific effects such as protein aggregation or denaturation so that false positives can be ruled out.^169^

**Figure 28:**
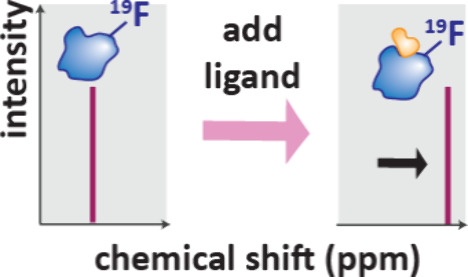
PrOF, binding of ligands causes changes in the chemical shift of ^19^F enriched proteins.

In 2023, Banci, Luchinat and colleagues demonstrated that ^19^F enriched proteins could be expressed in human cells. Shifting or disappearance in the ^19^F NMR signals of fluorinated carbonic anhydrase enzyme (CA2) in human cells or lysate could be detected when adding known ligands.^170^

## 6 Structural Biology

This section will describe the various techniques that can be used to provide structural information about protein–ligand complexes. X-ray crystallography has been the gold standard to determine whole protein structures at atomic resolution with bound compounds, though cryo-EM is rapidly emerging as a new powerhouse in structure determination. SAXS is less frequently used to observe protein structure but can be useful for proteins that are hard to crystallize, as well as to observe global changes in the structure of proteins. The AlphaFold platform has begun to transform computational structural biology by predicting protein folding using the amino acid sequence. This can help with model building when solving structures from electron density maps.^171^

### 6.1 X-ray Crystallography

X-rays diffract when they interact with electrons in crystalline samples of proteins. This creates a distinctive diffraction pattern which can be converted to an electron density map. A model of the protein is built into this map and gradually refined to solve the structure (Figure 29).^172^ X-ray crystallography is an invaluable tool for medicinal chemists in rational structure-based drug design. Though lacking any quantitative information, the atomic resolution of proteins and protein–ligand complexes in crystal structures enables the identification of key binding interactions to rationalize SAR.

**Figure 29:**
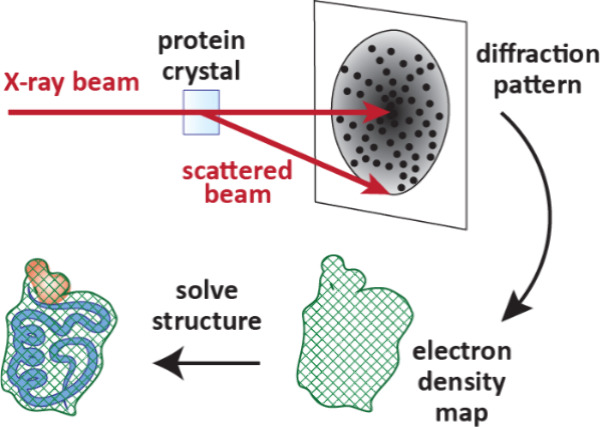
X-ray crystallography of protein–ligand complexes. Crystals of protein–ligand complexes are grown and optimized. They are then subjected to an X-ray beam to create a diffraction pattern which is used to generate an electron density map. Models of the protein are fitted to the map and refined to resolve the structure.

X-ray crystallography requires diffraction quality crystals which can be challenging to obtain and can have high protein consumption. However, new techniques are emerging to reduce the amount of protein required including cryocooling, microfocus beamlines and new detector technologies.^20^

As well as growing crystals in the presence of bound small molecules to obtain a single data set (cocrystallization), ligands or cocktails of ligands can be soaked into protein crystals to enable hit discovery with X-ray crystallography. Using this method, difference electron density maps are generated by comparing the electron density maps with and without the ligand. This process can be automated, enabling ranking of small molecules and fragments with immediate determination of the binding site.^173^ It is important to note that crystallography only gives a snapshot of a crystalline structural form of a protein–ligand complex, and although a useful model, the structure may be different in solution.^174^

### 6.2 Serial Crystallography (SX)

X-ray crystallography usually acquires a complete data set from a single crystal that has been cryocooled. Serial crystallography, however, generates a complete diffraction data set from combining X-ray data from many single crystals that are not cryoprotected. For SX, protein crystals can be smaller than standard methods and it avoids the need for optimization of cryoprotectant conditions.^175^ SX is still an emerging technique, and the technology is still improving, for example X-ray free electron lasers (XFELs) now enable room temperature analysis of micro- or nanometer sized crystals.^176^

### 6.3 Cryogenic Electron Microscopy (Cryo-EM)

Cryo-EM is an imaging technique that is beginning to compete with X-ray crystallography in terms of resolution and throughput in structural biology.^177^ It is particularly useful for large proteins (>150 Da) and those that are difficult to crystallize, such as membrane bound proteins. In cryo-EM, proteins are added in a thin layer on a grid and flash frozen in cryogenic fluid which preserves their solution structure. Still at low temperature, electrons are fired at the sample and their resulting scattering patterns are detected. 2D images from various angles are generated that are processed to construct a 3D electron density map (Figure 30). Like X-ray crystallography, models of the protein are compared with the electron density map to solve the structure.^178^ Improvements in both resolution and model building in cryo-EM are now enabling the direct observation of protein–ligand interactions.^179^

**Figure 30:**
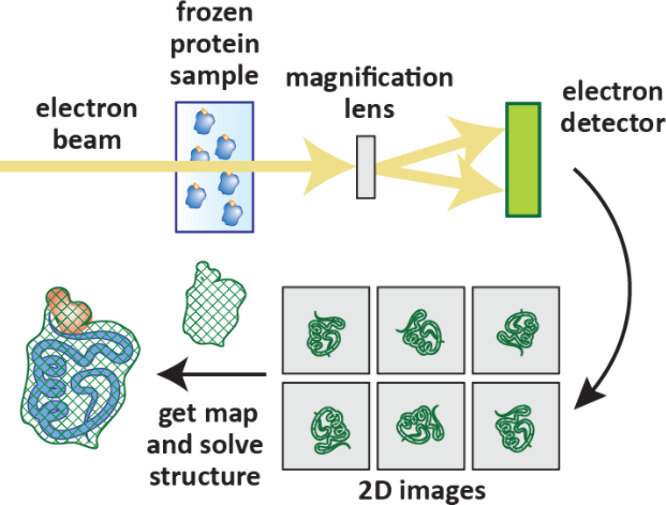
Cryo-EM uses electron scattering of frozen protein solutions to create 2D images of proteins which are aligned and averaged to make a 3D electron density map.

Cryo-electron tomography (cryo-ET) is a related and emerging technique which enables, for the first time, the observation of macromolecules in a cellular environment.^180^ Improvements in this methodology including sample preparation and data processing will enable research with cryo-ET to grow in coming years,^180^ potentially enabling imaging of small molecule protein interactions.

### 6.4 Small Angle X-ray Scattering (SAXS)

SAXS analyses the Rayleigh scattering (where there is no energy change on scattering) of X-rays as they interact with freely rotating molecules in solution, generating a scattering profile that provides information about the average distance distribution between all the atoms (Figure 31).^181^ In the context of determining drug–protein target engagement, X-rays are fired through solutions of protein–ligand complexes and the contributions from a blank buffer solution are subtracted to give a difference pattern for the macromolecule. This can provide structural information such as protein folding/unfolding, oligomerization and flexibility.^182^

**Figure 31:**
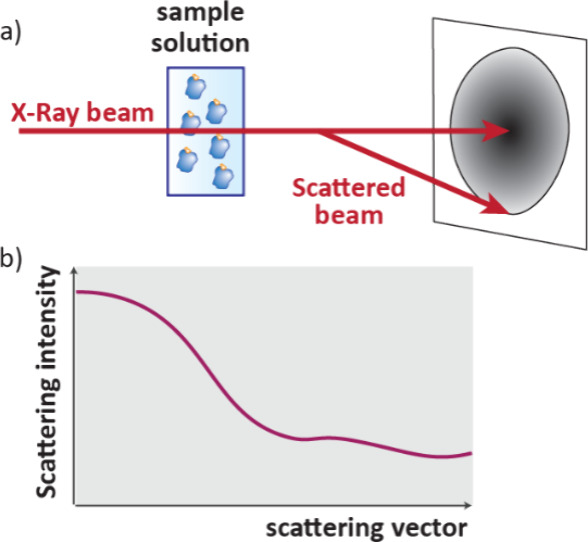
SAXS of protein ligand complexes in solution. a) An X-ray beam is diffracted by freely rotating proteins in solution. b) The shape of the intensity vs vector plot can provide information directly or derivatizations of the data can provide further insight.

SAXS is particularly useful to gather structural information on proteins where attempts at crystallization have failed. It can be used on any size protein varying from kilodaltons to gigadaltons,^183^ although solutions must be dilute to minimize intermolecular effects^181^ and buffer solutions must very accurately match that of the sample. To determine protein–ligand interactions, a mixture of protein, ligand and protein ligand complex exists at equilibrium in the buffer, and the scattering profile represents a linear contribution of these different species. When the scattering intensities of each species are known by experiment or calculation, software can calculate the volume fractions of each component in the mixture.^183^ Titration curves with ligands can therefore be generated to determine *K*_D_ values.^181^ Solubility of the ligands is very important as insoluble compounds can clog fluidics systems and provide unusable SAXS data.^182^

SAXS can be coupled with size exclusion chromatography (SEC-SAXS) where the sample eluted from the SEC column is rapidly exposed to the X-rays continuously through a capillary.^184^ Time resolved SAXS has also recently emerged to monitor the structural kinetics of protein ligand binding.^185^ SAXS is not widely used in screening of ligands against protein targets due to the difficulty in developing robust methods. Nevertheless, titrations of compounds have been carried out against histidine binding protein (HisBP) to monitor structural changes on ligand binding at a throughput of 20 to 100 compounds per day using synchrotron beamlines.^186^

A similar technique, small angle neutron scattering (SANS), uses a beam of neutrons which scatter at the nuclei of the atoms, instead of X-rays which scatter at the electrons. It is less widely used however due to the lower intensity of neutron sources which greatly limits the throughput.^181^

### 6.5 Microfluidic Modulation Spectroscopy (MMS)

MMS is an infrared (IR) spectroscopy tool which can be used to determine the secondary structure of proteins.^187^ It observes the amide C=O region between 1600 and 1700 cm^–1^ of the IR spectra and compares this in real time to a reference buffer solution (Figure 32a) to give a difference spectrum (Figure 32b). The stretching frequency of different amides along the amino acid backbone of a protein, e.g., beta sheets, alpha helices etc. are different. Therefore, if binding of a ligand changes the secondary structure of the protein, MMS can be used to monitor protein–ligand binding (Figure 32c). MMS can identify if a protein has become unfolded or aggregated and so can also be used to observe differences in thermal stability of proteins (changes in *T*_M_) upon addition of a ligand.

**Figure 32:**
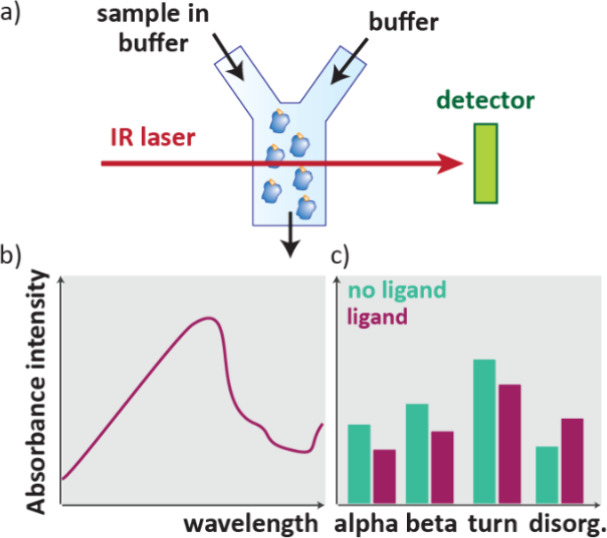
Microfluidic modulation spectroscopy. a) MMS takes an IR spectra of a protein solution and compares it in real time to a buffer solution by modulating between the sample and buffer solution and producing a difference spectrum. b) The difference spectrum is the IR absorbance in the amide C=O region of the sample minus the buffer solution. c) Difference spectra can be compared with and without ligand to show different levels of protein secondary structures.

## 7 Resonance Energy Transfer (RET)

RET is a nonradiative energy transfer from a donor to an acceptor. It requires the donor and acceptor molecules to be in close proximity (10–100 Å apart) and has been widely used to determine ligand binding to proteins. RET can occur through fluorescence (FRET) or bioluminescence (BRET).^188^ AlphaScreen is a similar technique, but energy is transferred from a transient singlet oxygen rather than directly from a donor molecule. These assays can be miniaturized and readouts measured using a fluorescence plate reader, so they are commonly used in HTS. Notably, however, aggregation can scatter signals and produce false results in assays with fluorescent readouts, though adding detergents can minimize this effect.^47^

### 7.1 Förster/Fluorescence Resonance Energy Transfer (FRET)

FRET occurs when a donor fluorescent dyes emission spectrum overlaps with an acceptor dyes excitation spectrum.^189^ A donor fluorophore label on the protein is electronically excited by light, and then the energy is transferred to a proximal acceptor fluorophore on a ligand (or *vice versa*) and the light emitted from fluorescence of the acceptor is monitored (Figure 33). FRET assays have been historically used to determine molecular distances as the FRET signal is proportional to the distance between the donor and acceptor fluorophore, though it is also used to determine *K*_D_ values.^190^

**Figure 33:**
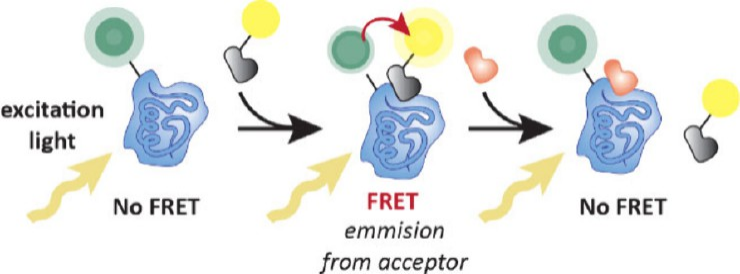
Principle of FRET to determine ligand binding. When in proximity, a fluorescent donor dye on the protein can transfer energy to an acceptor fluorescent dye bound to a ligand or reporter probe which will then fluoresce. Displacement of a FRET reporter by a competitive ligand will cause a loss in FRET signal and allows unlabeled ligands to be tested.

Although FRET requires both the protein and ligand to be labeled, competition experiments enable libraries of ligands to be tested by monitoring a loss of FRET signal from displacement of a reporter probe (Figure 33). High throughput assays to identify ligands that disrupt the PPI of two fluorescently labeled proteins have also been developed.^191^

As well as the requirement of labels, a major drawback of FRET is background fluorescence. This can be overcome with Time-Resolved Fluorescence Resonance Energy Transfer (TR-FRET).^192^ In TR-FRET, a lanthanide such as europium with a long half-life is used as the fluorescence donor. Displacement of a TR-FRET acceptor molecular probe has enabled the high throughput screening of compound libraries.^193^ Homogeneous Time-Resolved Fluorescence (HTRF) is a commonly used TR-FRET based assay developed by Cisbio.^192^ FRET is most accurately measured using fluorescence lifetime imaging microscopy (FLIM), which enables the monitoring of target engagement of labeled ligands in cells.^194^ It has been shown that it is possible to use native tryptophan residues on certain proteins as intrinsic FRET donors or acceptors (iFRET) to monitor protein–ligand interactions.^195^

### 7.2 Bioluminescence Resonance Energy Transfer (BRET)

The issues of background fluorescence and photobleaching in FRET can be overcome by using bioluminescence energy transfer in BRET assays.^188^ In BRET, the bioluminescent donor is luciferase, which undergoes bioluminescence as it oxidizes luciferin in the presence of O_2_ and a cofactor such as ATP. The excitation in BRET occurs by addition of a substrate (luciferin) as opposed to using light, which means that it avoids photobleaching. BRET is commonly used in live cell imaging, in particular to monitor protein–protein interactions.^196^

nanoBRET is an advanced version of BRET which uses a bright luciferase (Nanoluc) with a fluorophore of a long emission wavelength, which has led to improved light intensity and spectral resolution.^197^ nanoBRET in a competitive assay format (Figure 34) has enabled the quantification of drug-target engagement and residence time in live cells.^198^ This competitive assay has been developed for high throughput screening applications for small molecules^199^ and similarly for inhibitors of PPIs.^200^ Though not commonly performed for kinetic analysis, nanoBRET has been used to measure binding kinetics for GPCRs.^201^ Although usually used for protein quantification as an alternative to immunoassays, HiBit is a small section of nanoluciferase which can be used as a label on a protein. On addition of the larger luciferase segment, “Lgbit”, bioluminescence is achieved which can act as a donor for BRET assays. This protocol has been successfully used for measuring target engagement for membrane bound melatonin receptors in living cells.^202^

**Figure 34:**
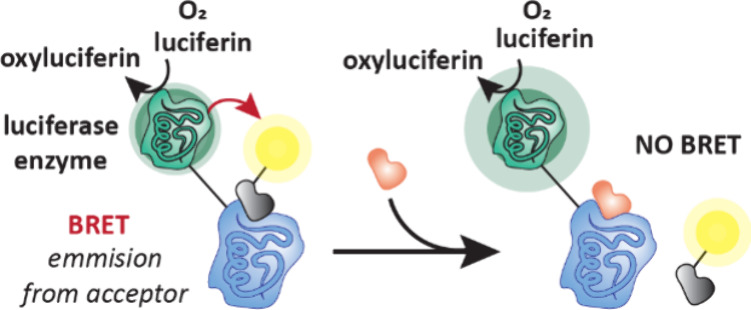
Principle of competitive BRET. When a reporter molecule with an acceptor fluorophore label is bound to the protein with a pendant luciferase, BRET occurs. When the ligand binds it competes off the acceptor fluorophore probe resulting in a loss of the BRET signal.

### 7.3 ThermoFRET and ThermoBRET

ThermoFRET^203^ and ThermoBRET^204^ assays are used to study the influence of ligands on the thermal stability of membrane bound proteins such as GPCRs. The proteins are solubilized using detergents before being subjected to a temperature gradient. The proteins will unfold at their melting temperature, revealing cysteine residues which react with a thiol reactive acceptor dye which then enables BRET or FRET from the donor to acceptor to occur (Figure 35). The fluorescence is monitored and changes in *T*_M_ at different ligand concentrations can be determined.

**Figure 35:**
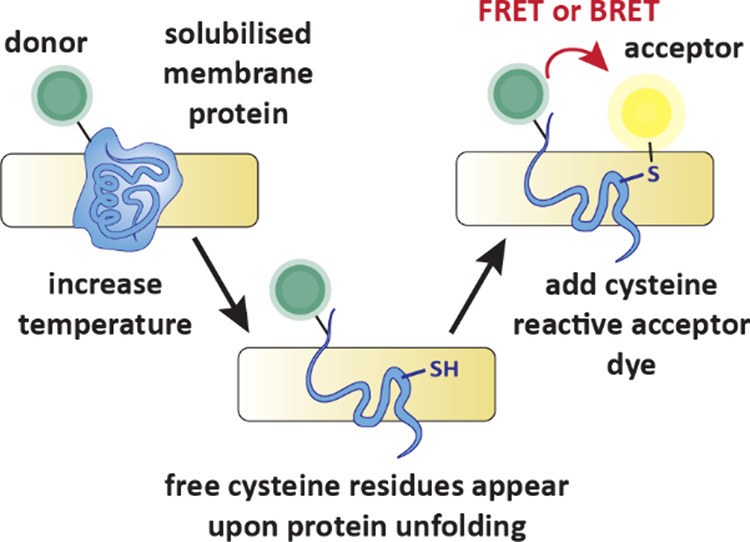
Principle of ThermoFRET and ThermoBRET to determine ligand binding. A membrane protein which has been labeled with a FRET or BRET donor is solubilized by detergent. The temperature is increased which unfolds the protein and reveals free cysteine residues. A cysteine reactive dye is added which binds to the unfolded protein and acts as a FRET or BRET acceptor to produce a fluorescent signal. Ligand binding can thermally stabilize the protein resulting in a lower signal.

### 7.4 Amplified Luminescent Proximity Homogeneous Assay (AlphaScreen)

Like FRET and BRET, AlphaScreen assays also involve energy transfer from a donor to an acceptor.^205^ In AlphaScreen, two molecules of interest are bound to separate donor and acceptor beads, energy transfer between the beads takes place when they are in proximity, resulting in a chemiluminescent signal (Figure 36).^206^ The donor bead contains a photosensitizer (compounds that absorb light and change the course of a chemical reaction) which is excited by 680 nm light, the excited photosensitizer then converts oxygen to singlet oxygen. The singlet oxygen diffuses away from the donor and transfers energy to the acceptor bead that contains a series of organic dyes that emit light. Singlet oxygen has a limited half-life in solution and so only diffuses a short distance before being quenched, which enables the proximity-based function of AlphaScreen.

**Figure 36:**
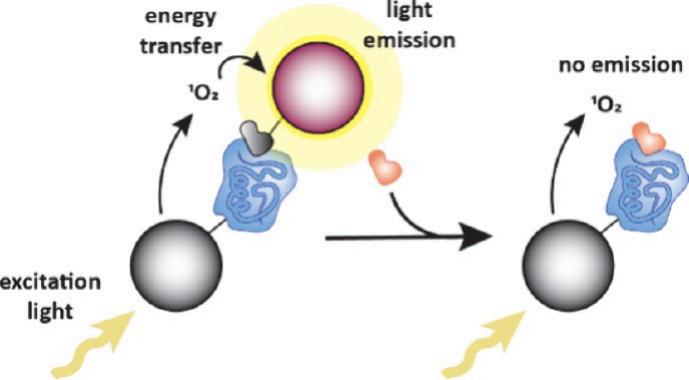
Principle of competitive Alpha assays. Proximity of a photosensitizer bead and acceptor dye bead results in chemiluminescence by energy transfer from singlet oxygen which is formed at the photosensitizer donor bead.

AlphaLISA and AlphaPlex variants have been developed with modified acceptor beads which emit light at different wavelengths. Since their development, Alpha technologies have been shown to be useful for HTS, including for challenging PPI inhibitors.^205^

## 8 Other Binding Assays

### 8.1 Fluorescence Polarization (FP)

In FP binding assays, polarized light is used to excite a fluorescent probe tethered to a ligand; the polarization of the light emitted from the probe is dependent on how freely the dye molecule can rotate (Figure 37).^207^

**Figure 37:**
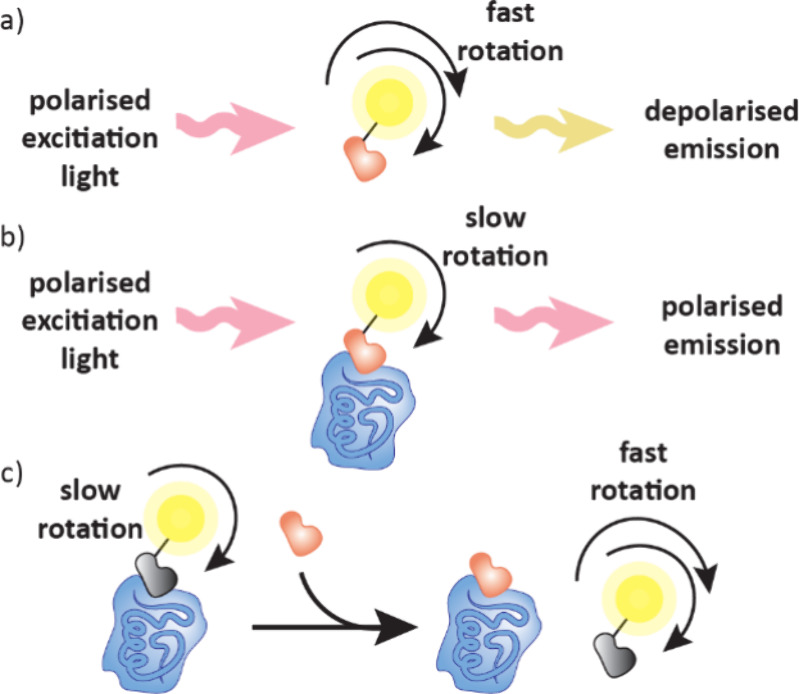
Principle of an FP assay where fluorescent dyes attached to a ligand are excited by polarized light. a) Freely rotating dyes in solution emit depolarized light while b) dyes on ligands bound to proteins do not lose the polarization of emitted light. c) Competition experiments in FP assays enable ligands without a fluorescent label to be screened.

A major drawback of FP assays in this format is the requirement of having a label tethered to the ligand, which can affect the binding properties. For screening, competition experiments are used where a reporter ligand with a pendant dye is displaced by another, more strongly binding ligand (Figure 37c). This enables FP binding assays to be used in HTS.^207^ FP assays can also have suitable sensitivity to detect protein binding in cell lysates.^208^ Like other assays with fluorescence readouts, FP assays can encounter problems if aggregation occurs.

### 8.2 Spectral Shift (SS)

Spectral shift was first developed in 2022 and measures small changes in fluorescence upon ligand binding with a protein labeled with a near-infrared fluorophore.^209^ Subtle changes in the microenvironment of the dye molecule occur upon ligand binding which results in hypsochromic (blue) or bathochromic (red) shift of the fluorescence spectra (Figure 38a). The ratio of fluorescence at two preselected wavelengths is measured using photon-multiplier tubes which leads to increased sensitivity, and ligand titrations are used to measure *K*_D_ values (Figure 38b).

**Figure 38:**
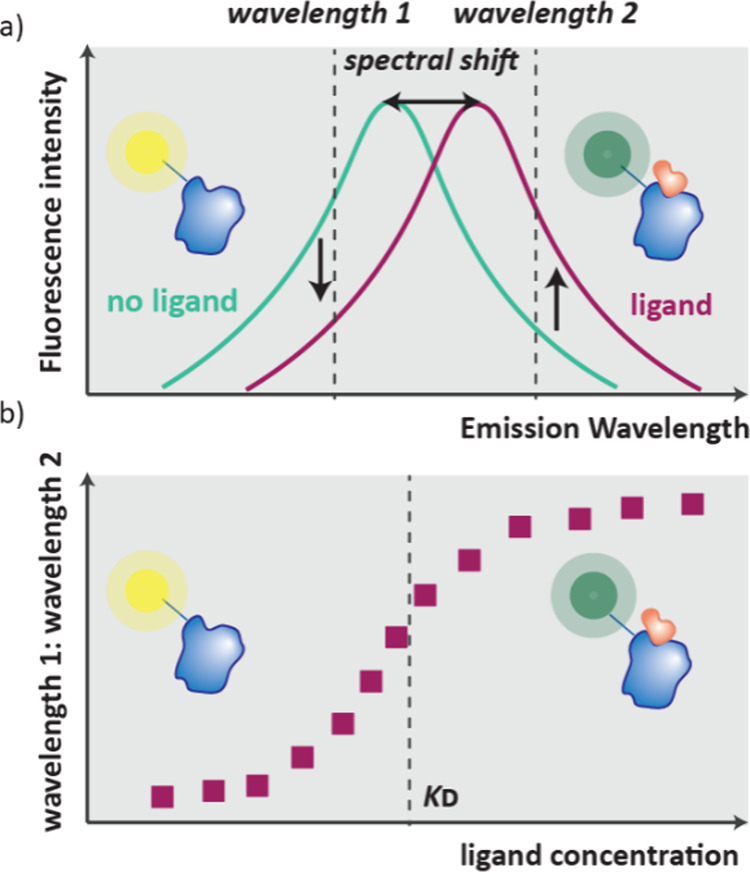
a) In spectral shift assays, subtle changes in fluorescence emission spectra of a dye labeled to the protein of interest that occur on ligand binding are measured. b) The ratio of two separate wavelengths at different ligand concentrations is plotted to determine *K*_D_.

### 8.3 Flow Induced Dispersion Analysis (FIDA)

The size of a molecule in solution alters its radial diffusivity which changes how much it is dispersed. In a FIDA measurement, solutions of protein–ligand complex are passed through a thin capillary, and the fluorescence is measured over time (Figure 39). Larger molecules will diffuse to a greater extent and FIDA uses this observation to directly measure the hydrodynamic radius of a particle.^210^ Fidabio have commercialized this technology in the FIDA Neo instrument which, as well as direct *K*_D_ measurements, can be used to measure kinetic parameters *k*_on_ and *k*_off_ without the need for immobilization.

**Figure 39:**
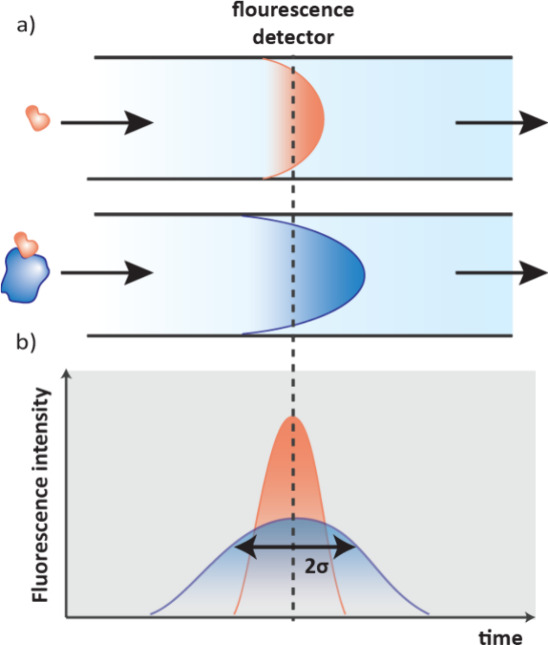
a) Large and small molecules diffuse differently when undergoing laminar flow through a capillary. b) Fluorescence over time is monitored, larger molecules diffuse at a faster rate so have a different shaped signal.

### 8.4 Radioligand Binding Assays

First miniaturized in 2005,^211^ radioligand binding assays are commonly used to monitor drug target engagement of cell surface receptors in cells. They measure the affinity of a ligand containing a radiolabel and can be used to determine *K*_D_ values as well as the density of receptors in cells or tissues. These assays can also be used to determine binding mechanisms and rate constants.^20^ Radioligands typically contain either ^135^I or ^3^H, but ^35^S, ^32^P or ^33^P can also be used.^212^

Displacement of reporter molecules (Figure 40) enables screening of unlabeled small molecule ligands in a competitive assay format.^213^ Pfizer has used a radioligand binding assay to screen their library of >500,000 compounds in the discovery of Maraviroc.^24,214^ As well as receptors, radioligand binding assays are useful to measure the binding of ion channels.^215^ Operationally, the use of radiolabeled ligands requires the appropriate safety precautions.

**Figure 40:**
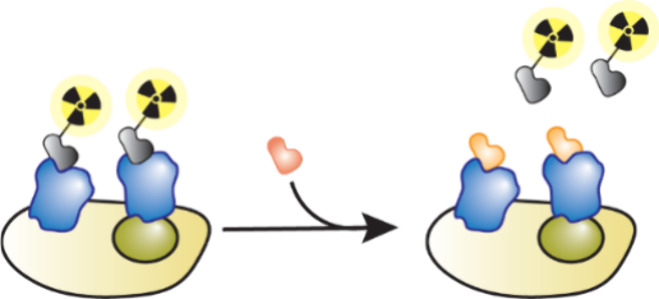
A competitive radioligand binding assay. Reporter radioligands are bound to receptors on a cell surface. Addition of a ligand displaces the radiolabeled ligand. Bound and free ligands are separated and the radioactivity of the unbound radioligands is measured.

### 8.5 Fluorescence Microscopy (FM)

Various techniques that produce a fluorescent signal on protein–ligand binding can be used to image target engagement in live cells using fluorescence microscopy (Figure 41).^216^ For example, a BRET signal between Nanoluciferase labeled HDAC1 and a reporter ligand with a BRET acceptor can be switched off when adding known inhibitors, and fluorescence over time can also be monitored enabling determination of residence time.^217^

**Figure 41:**
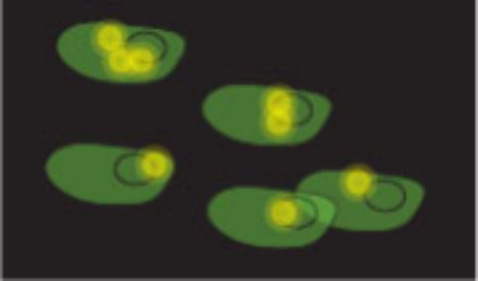
Imaging with fluorescence microscopy can be used to monitor target engagement in cells in real time.

## 9 Chemoproteomics

All the methods discussed in this Perspective thus far, analyze the interactions of ligands to a single protein of interest. Chemoproteomics, however, monitors drug-target interactions across all proteins in a living system (the whole proteome) and is beginning to unlock a new paradigm in drug discovery.^218^ Chemoproteomics is an incredibly useful tool for target deconvolution. It can be used to pinpoint which proteins a molecule is interacting with after identifying a desirable compound with an unknown mechanism of action using phenotypic drug discovery against a disease model.^219^ In target-based drug discovery, chemoproteomics can be used to identify new chemical matter for potential drug targets,^220^ including those previously considered “undruggable”.^221^

The analysis of protein–ligand interactions across the proteome of a living system can be performed with traditional proteomic analysis such as gel electrophoresis,^222^ though advances in workflows of MS-based proteomics now enables proteomes to be screened in as little as a few hours.^223^ Affinity based protein profiling (ABPP) requires reporter groups on the ligand to enable separation of bound and unbound proteins. This labeling can have implications on how the ligand binds and its physicochemical properties. Label-free methods have also been developed, such as Thermal Proteome Profiling (TPP), Drug Affinity Responsive Target Stability (DARTS) and Stability of Proteins from Rates of Oxidation (SPROX) which avoid the drawbacks of labeling but in some cases can only be used on cell lysates as opposed to live cells.

### 9.1 Affinity-Based Proteome Profiling (ABPP)

In ABPP, the identification of which proteins a ligand is binding after incubation in cells is based on “affinity enrichment” of the protein compared to a control. Notably, the extraction of protein mixtures from live cells in cell lysis results in the release of noncovalent ligands. Due to this, ABPP with live cells is largely used with covalent drugs,^35^ or reversible ligands that are functionalized with photoaffinity tags such as a diazirine which on illumination loses N_2_ to form a carbene that rapidly reacts with proteins associated with the ligand (Figure 42a).^223^ However, reversible binding ligands can be used for experiments in cell lysates, where there is no lysing step, or in competition with tool compounds in live cells. As well as a reactive covalent binding group, ligands used for ABPP are functionalized with an affinity tag such as biotin or an alkyne for further derivatization which allows discrimination and separation of ligand bound and ligand unbound proteins (Figure 42b). As in bottom-up MS, the proteins are subsequently digested with a protease enzyme into peptide fragments which are separated and analyzed by LCMS to give quantitative data on which proteins are present in the sample (Figure 42c). This data is compared with a control to determine how much enrichment of each protein was observed on addition of the ligand. Multiple proteomic experiments can be analyzed by MS contemporaneously using isobaric labels such as tandem mass tag (TMT), increasing throughput.^224^ Notably, ABPP with bottom up-MS proteomics can reveal binding site information, as the specific peptide fragment which has been covalently functionalized by the ligand is visible in the mass spectrum.^35^

**Figure 42:**
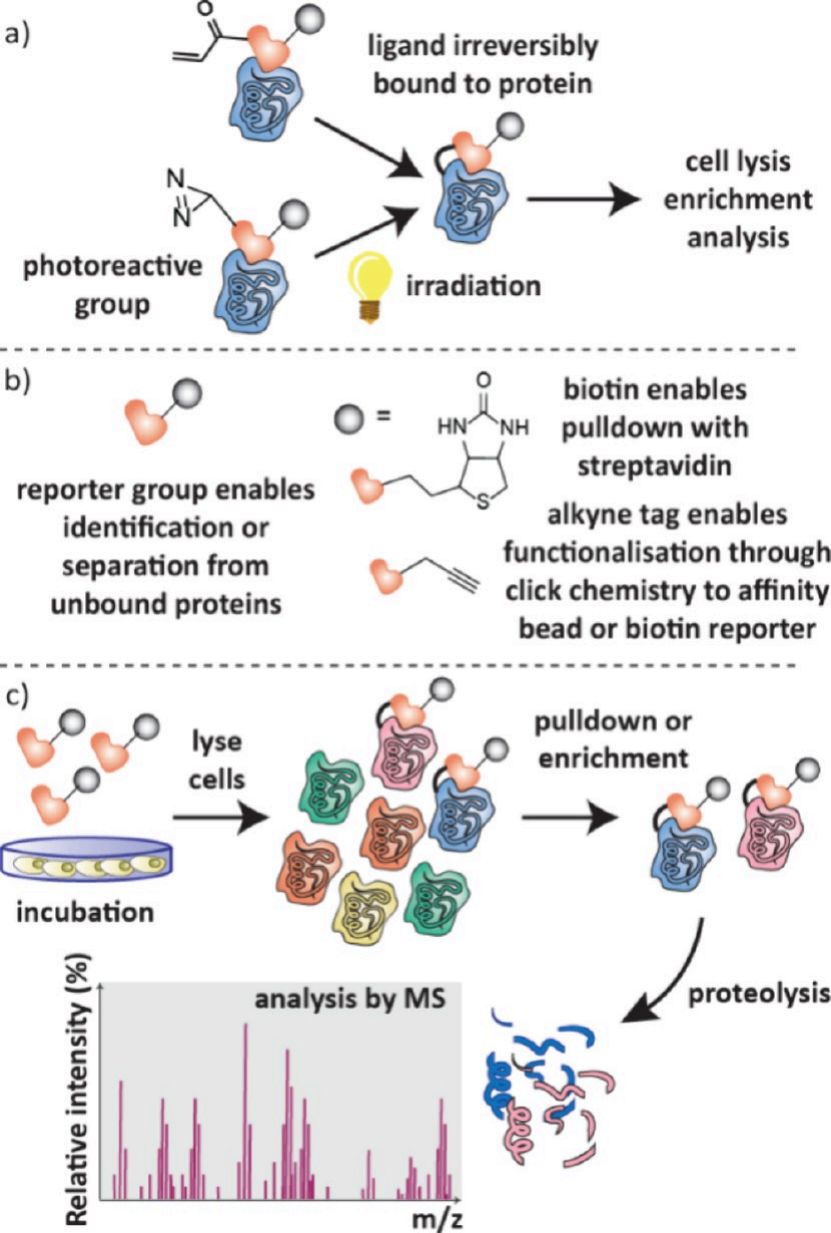
a) In ABPP with live cells, ligands must be covalently bound to the protein, this can be achieved with covalent ligands or reversibly binding ligands with a photoaffinity group which on irradiation form covalent bonds to associated proteins. b) Reporter groups are required on the ligands in ABPP to enable the separation of proteins with ligands bound from nonbinding proteins. c) In ABPP, ligands containing a reporter group are incubated with cells where they form covalent interactions with their protein targets. The cells are then lysed, and the ligand binding proteins are separated from the resultant protein mixture by a mechanism specific to the reporter tag. The proteins are then digested with a protease and the fragments analyzed by LCMS which quantifies how much of each protein was in the digested protein mixture.

ABPP can be suitably sensitive for fragment-based drug discovery. Initially this was limited to covalent fragments,^225^ however very recently photoaffinity tagged fragments have been used, which with TMT reagents enabled proteome-wide screening of 6,000 reversibly binding fragments. Nearly 50k fragment-protein interactions were identified using this method and this large data set facilitated the training of AI modeling tools for predictions of the promiscuity and potential protein target classes of novel fragments.^220^

### 9.2 Thermal Proteome Profiling (TPP)

TPP is a label-free chemoproteomics method which combines the concepts of the cellular thermal shift assay (CETSA, see section 3.4) and MS-based proteomics. It enables analysis of the effect of ligand binding on melting temperature across the whole proteome of living cells.^226^ Like CETSA, TPP relies on the fact that when proteins denature, they become insoluble and thus can be separated from soluble, intact, protein. The workflow for TPP is the same as CETSA, in that ligands are incubated with live cells which are subsequently subjected to temperature changes to induce denaturation (see Figure 6). In TPP however, mass spectroscopy enables quantification of the intact amount of each protein in the proteome, not just a single target. Changes in the melting temperature caused by ligands can be small, however, which can lead to false negatives.^219^ By cross-examining the profiles of cell extracts and intact cells, TPP can discriminate whether changes on protein stability caused by a ligand are because of direct binding or downstream effects.^227^

### 9.3 Drug Affinity Responsive Target Stability (DARTS)

The binding of ligands can stabilize proteins toward proteolysis. In DARTS, ligands are incubated with cell lysates and proteins that are not stabilized by the ligand are digested and separated so that the intact proteins can be observed (Figure 43). DARTS was originally analyzed with gel electrophoresis and staining,^228^ though MS has also now been used.^229^ Like TPP, DARTS has the advantage that no labeling of the ligand or protein is required and so the setup is relatively simple. Conducting DARTS in living cells would enable observation of cell-membrane permeation and any downstream effects, though due to loss of reversible ligands in cell lysis, and the low abundance of some proteins, this has so far been difficult to achieve.^230^

**Figure 43:**
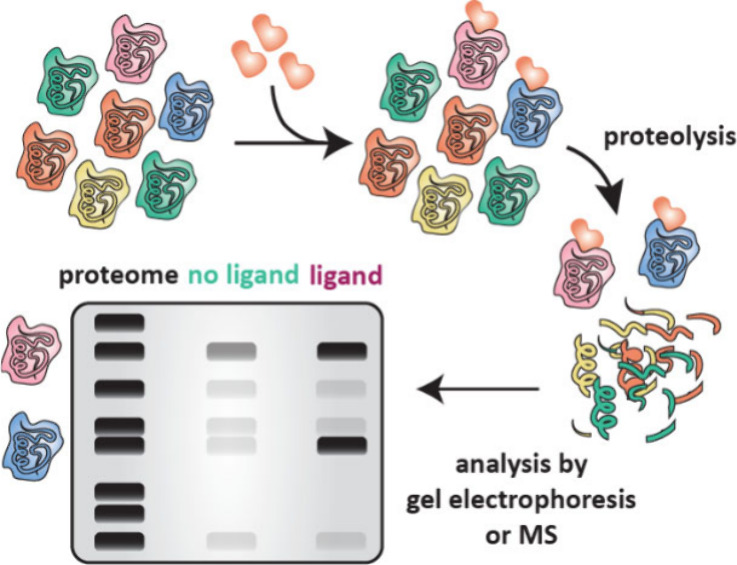
In DARTS, ligands are incubated with cell lysate and the resultant mixture is subjected to proteolysis conditions. Proteins with bound ligands are generally more stable to proteolysis, and the amount of whole protein remaining can be monitored by gel electrophoresis or MS.

Addition of urea can induce protein unfolding and thus increased susceptibility to proteolysis. Addition of ligands can stabilize proteins to this urea-induced proteolysis in a technique related to DARTs coined Pulse Proteolysis (PP).^231^ Similarly, other methods to probe protein–ligand binding by stability to proteolysis under specific conditions have been described.^229^

### 9.4 Stability of Proteins from Rates of Oxidation (SPROX)

Like DARTS, SPROX analyses the reactive stability of proteins on ligand binding. Whereas DARTS observes proteolysis stability, SPROX observes how easily methionine residues of proteins are oxidized by hydrogen peroxide in the presence of increasing concentrations of a chemical denaturant, with and without the presence of a ligand.^232^ It can be used to determine Gibbs energy changes as well as *K*_D_ values for ligands. The results from SPROX were originally determined by electrospray or MALDI-MS, though bottom-up proteomics MS coupled with TMT labeling can now be used to increase sensitivity and throughput.^233^

## 10 Conclusions and Outlook

Since new technologies have allowed drug discovery scientists to isolate and test single proteins, as well as an improved understanding of the MoA of various diseases, target-based drug discovery has arguably become the most important strategy in pharmaceutical research. TBDD enables the rational design of new drugs, in theory expediting the discovery of innovative new medicines. As a result, a plethora of techniques has emerged to quantify the degree of drug-target engagement on isolated proteins of interest. However, isolated proteins are a highly simplified model of living systems, and the importance of confirming drug-target engagement in more complex cellular systems cannot be understated and so many assays have also been developed for use in living cells. The advance of chemoproteomics now enables target engagement to be monitored across whole cellular proteomes. As throughput and sensitivity as well as data analysis using AI improves, chemoproteomics is likely to play an increasingly important role in drug discovery. Its use in screening compounds will grow, particularly if noncovalent binders can be more easily and accurately identified.

The methods discussed in this Perspective provide a vast toolkit for use in drug discovery to determine thermodynamic, kinetic and structural parameters of protein–ligand binding. Each method described has distinct advantages, trade-offs and levels of suitability that should be assessed for each specific drug discovery project, dependent on the protein of interest. The assays described are a testament to the efforts, imagination and innovative thinking by the scientific community to overcome challenges faced when developing much needed medicines for the treatment of disease.

## Abbreviations Used

ABPP: affinity-based proteome profiling
ACE: affinity capillary electrophoresis
Agr.: aggregation
AI: artificial intelligence
ALIS: automated ligand identification system
Aliph: aliphatic
Alpha: amplified luminescent proximity homogeneous assay
AS: affinity based selection
BLI: biolayer interferometry
BRET: bioluminescence resonance energy transfer
BSI: back scattering interferometry
CM: carboxymethyl
CIAS: collision-induced affinity selection
CD: circular dichroism
CETSA: cellular thermal shift assay
CPMB: Carr–Purcell–Meiboom–Gill
Cryo-EM: cryogenic electron microscopy
Cryo-ET: cryo-electron tomography
CSA: chemical shift anisotropy
DARTS: drug affinity responsive target stability
DEL: DNA encoded library
dISA: Dynamic Inhibition-in-Solution Assay
DLS: dynamic light scattering
DPI: dual polarization interferometry
DSC: dynamic scanning calorimetry
DSF: dynamic scanning fluorimetry
EFC: enzyme fragment complementation
EPF: epifluorescence
FAC: frontal affinity chromatography
FAXS: fluorine chemical shift anisotropy exchange
FIDA: flow induced dispersion analysis
FILM: fluorescence lifetime imaging microscopy
FM: fluorescence microscopy
FP: fluorescence polarization
GCI: grating coupled interferometry
HDX: hydrogen–deuterium exchange
INPHARMA: Interligand NOE for Pharmacophore Mapping
ITC: isothermal titration calorimetry
*K*_A_: association constant
*K*_D_: dissociation constant
*k*_on_: association rate constant
*k*_off_: dissociation rate constant
LO: ligand observed
MFS: magnetic force spectroscopy
MMS: microfluidic modulation spectroscopy
MST: microscale thermophoresis
MZI: Mach–Zhender interferometry
N: stoichiometry
Nanoluc: nanoluciferase
PAINS: pan-assay interference compounds
Photo-CIDNP: photochemically induced dynamic nuclear polarization
PO: protein observed
PP: pulse proteolysis
PrOF: ^19^F fluorine protein-observed NMR
PPI: protein–protein interaction
PUF: pulsed ultrafiltration
QCM: quartz crystal microbalance
*R*_2_: transverse relaxation rate
RM: resonant mirror
RWG: resonant waveguide grating
SALMON: solvent accessibility, ligand binding, and mapping of ligand orientation by NMR spectroscopy
SAMDI: self-assembled monolayer desorption ionization
SAW: surface acoustic wave
SAXS: small angle X-ray scattering
SEC: size exclusion chromatography
SHG: Second Harmonic Generation
SLAPSTIC: spin labels attached to protein side chains as a tool to identify interacting compounds
SLS: static light scattering
SOFAST-HMQC: band-selective optimized flip angle short transient heteronuclear multiple quantum coherence
SPROX: stability of proteins from rates of oxidation
SS: spectral shift
STD: saturation transfer difference
STINT-NMR: structural interactions by in-cell NMR
SX: serial crystallography
τ: residence time
*T*_M_: melting temperature
TBDD: target-based drug discovery
TC: ternary complex
TINS: target immobilised NMR Screening
TMT: tandem mass tag
TPP: thermal proteome profiling
TIRF: total internal reflection microscopy
TRIC: temperature related intensity change
TROSY: transverse relaxation-optimized spectroscopy
TSA: thermal shift assay
WaterLOGSY: water-ligand observed via gradient spectroscopy
WaveRAPID: repeated analyte pulses of increasing duration
XFEL: X-ray free electron laser
YI: Young’s interferometry
