# Correction to “Target Engagement Assays in Early Drug Discovery”

**DOI:** 10.1021/acs.jmedchem.5c02336

**Published:** 2025-09-03

**Authors:** Sahra St John-Campbell, Gurdip Bhalay

At the final stage of the proofing, the uploaded Table 1 contained no errors. In the editorial process, the table was modified so that headings and subheadings were repeated on each page, but some are incorrect and three lines from the table were deleted in this process.

**Table 1:**
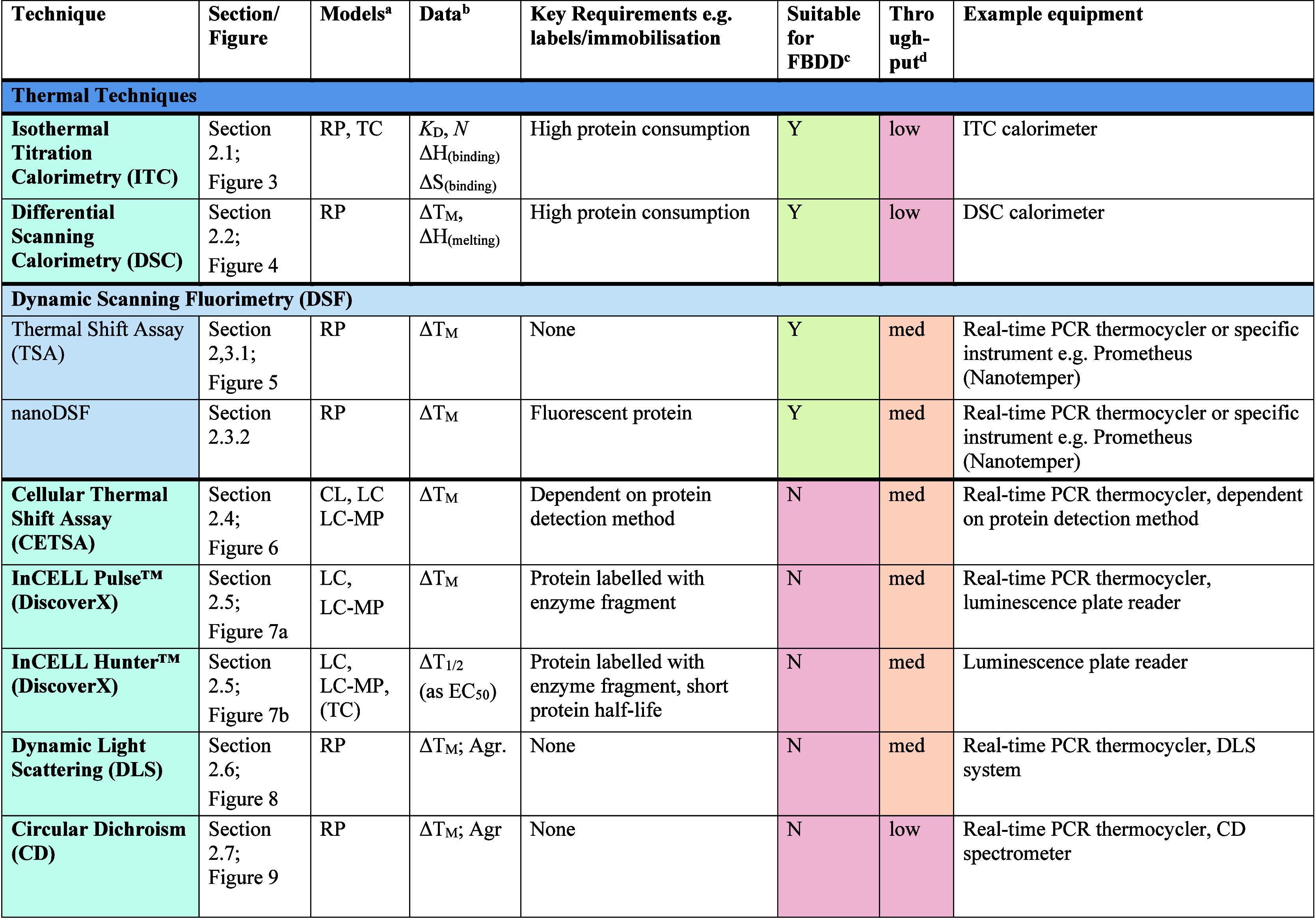

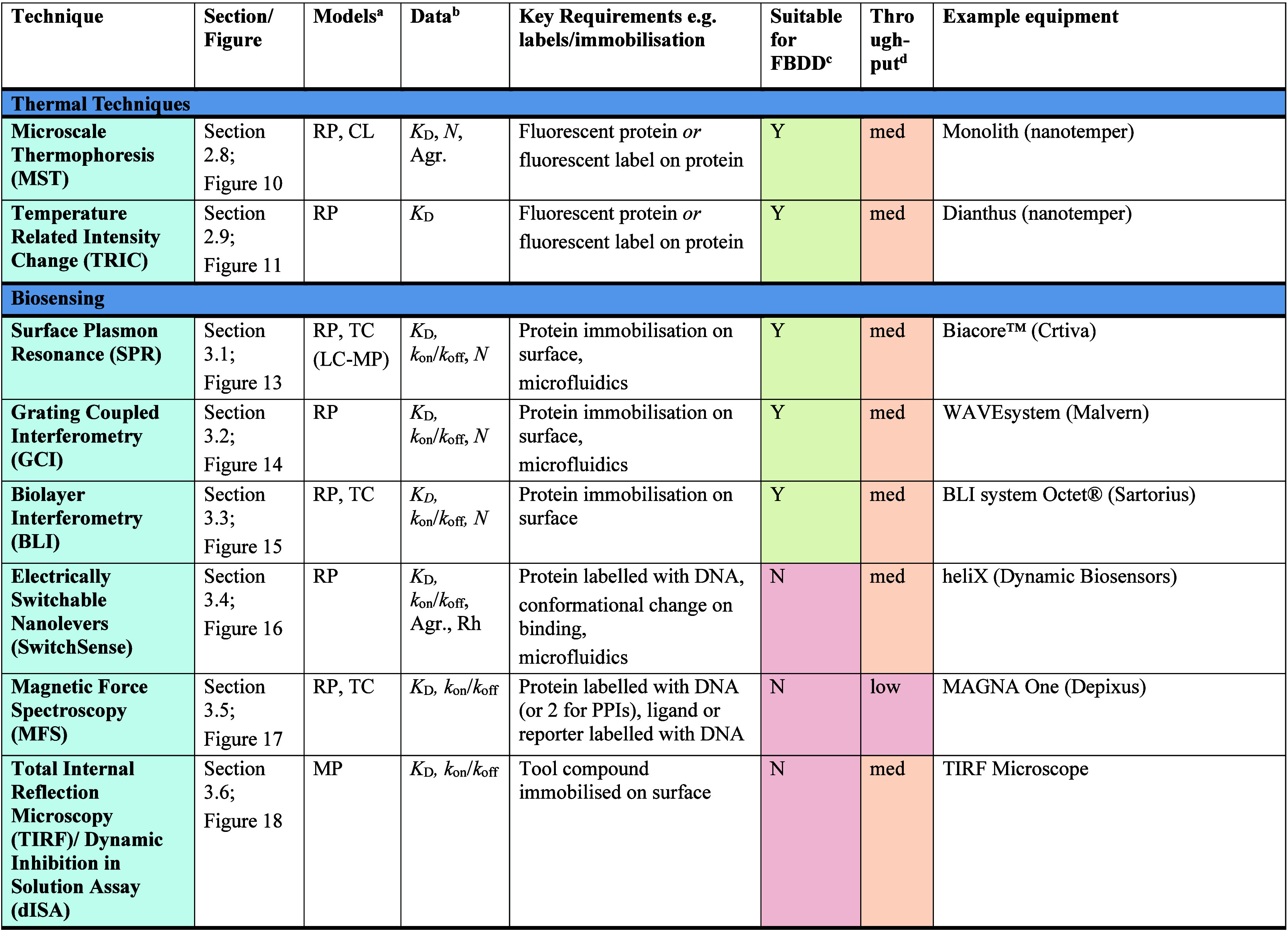

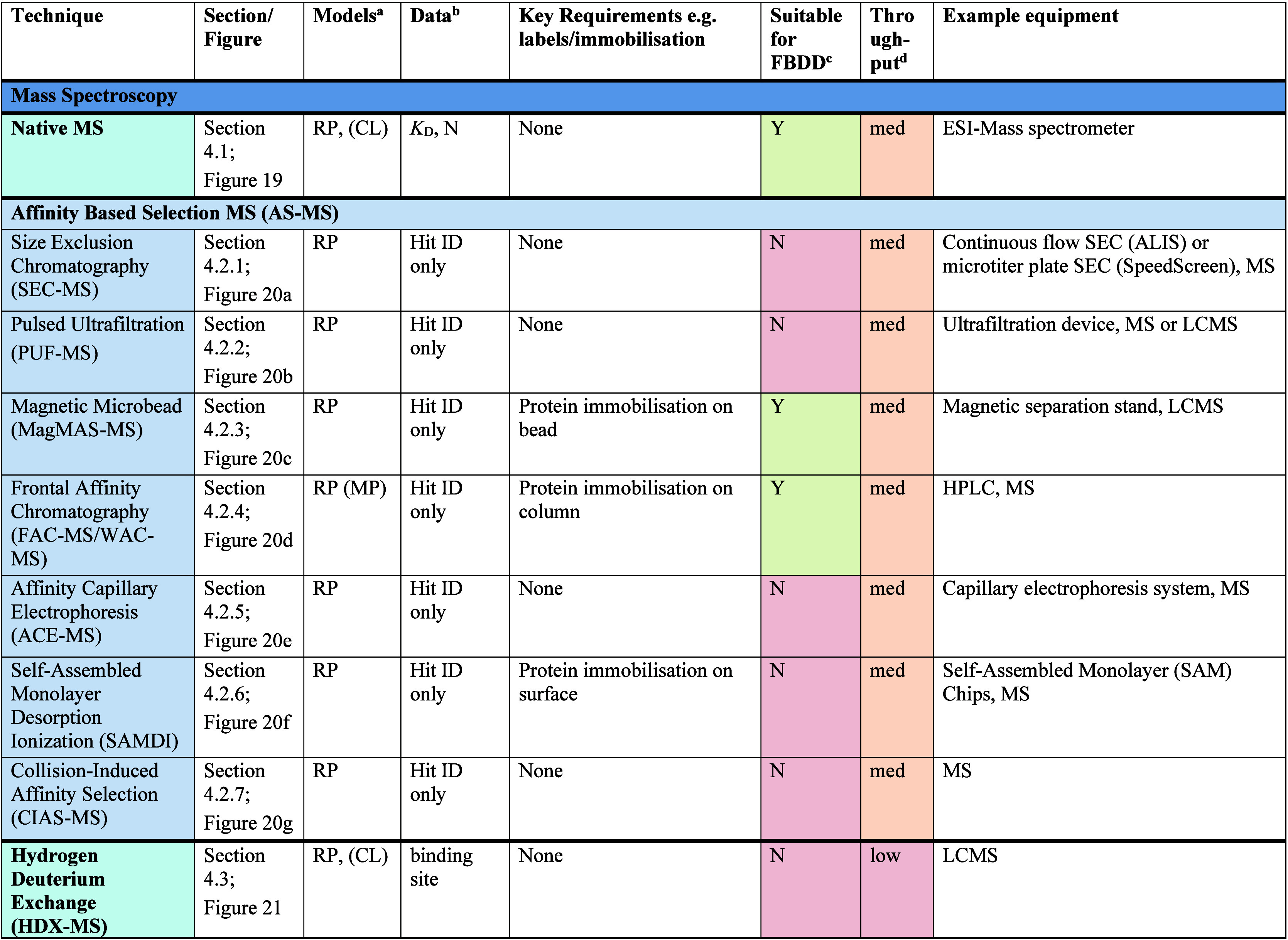

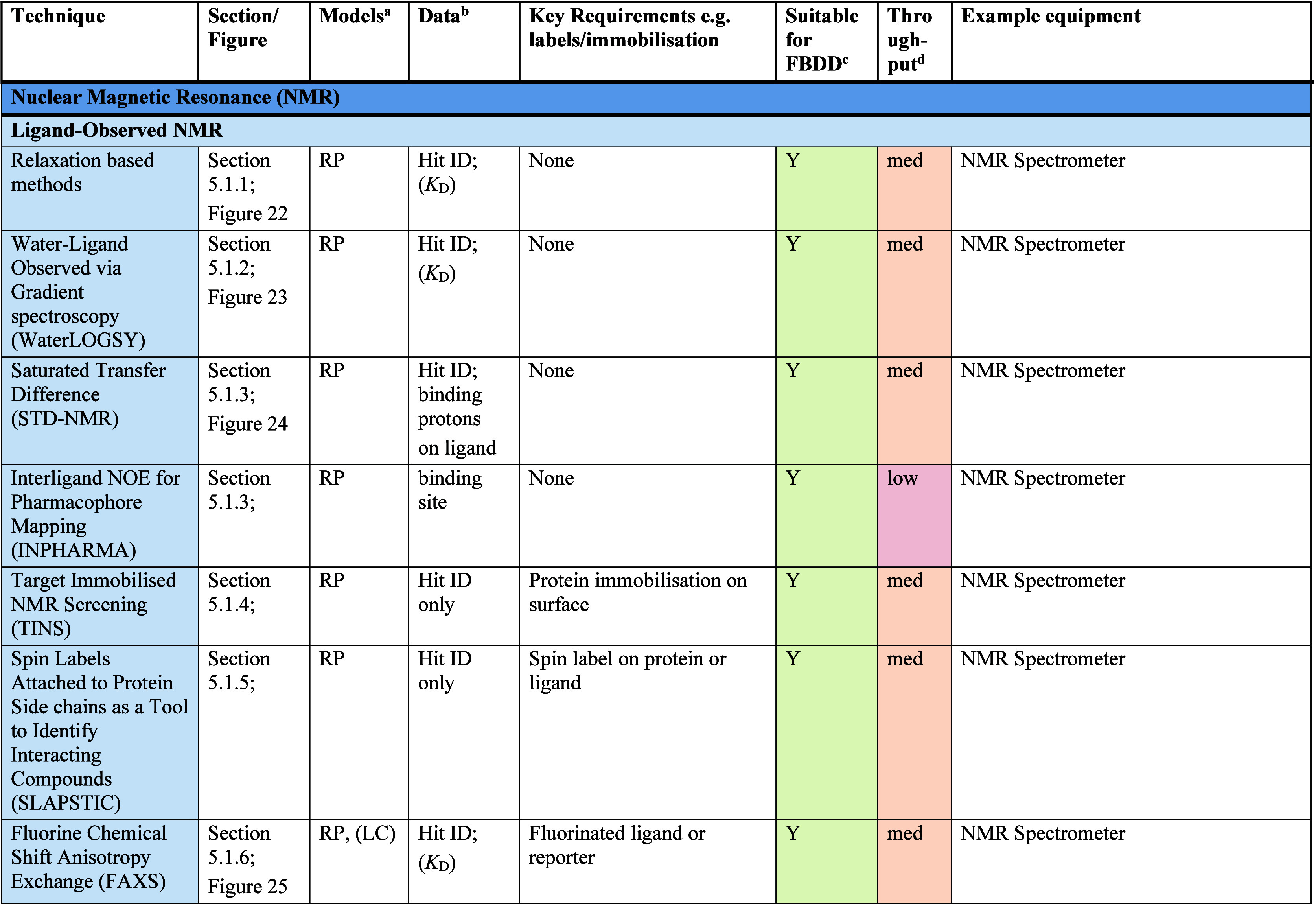

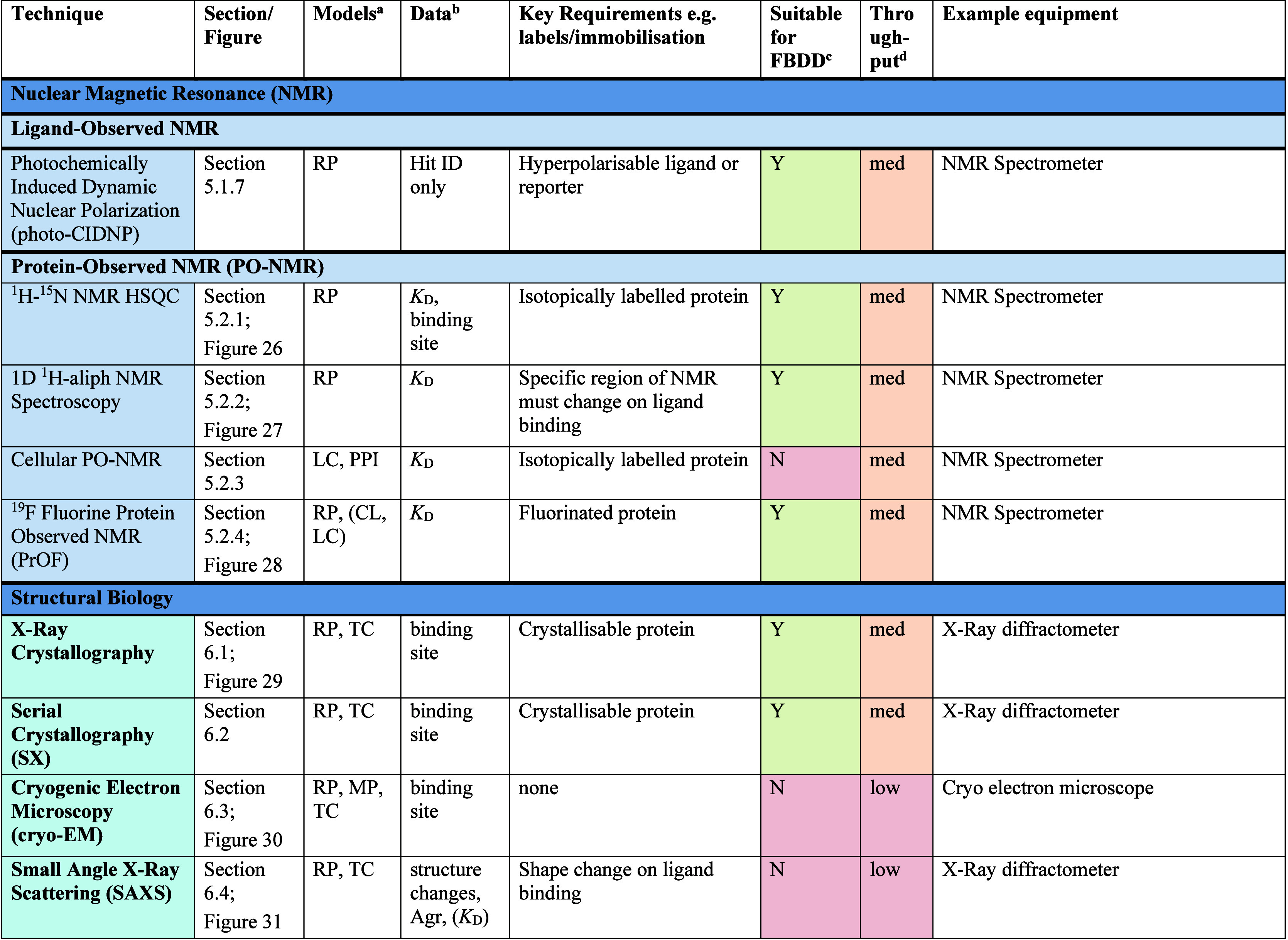

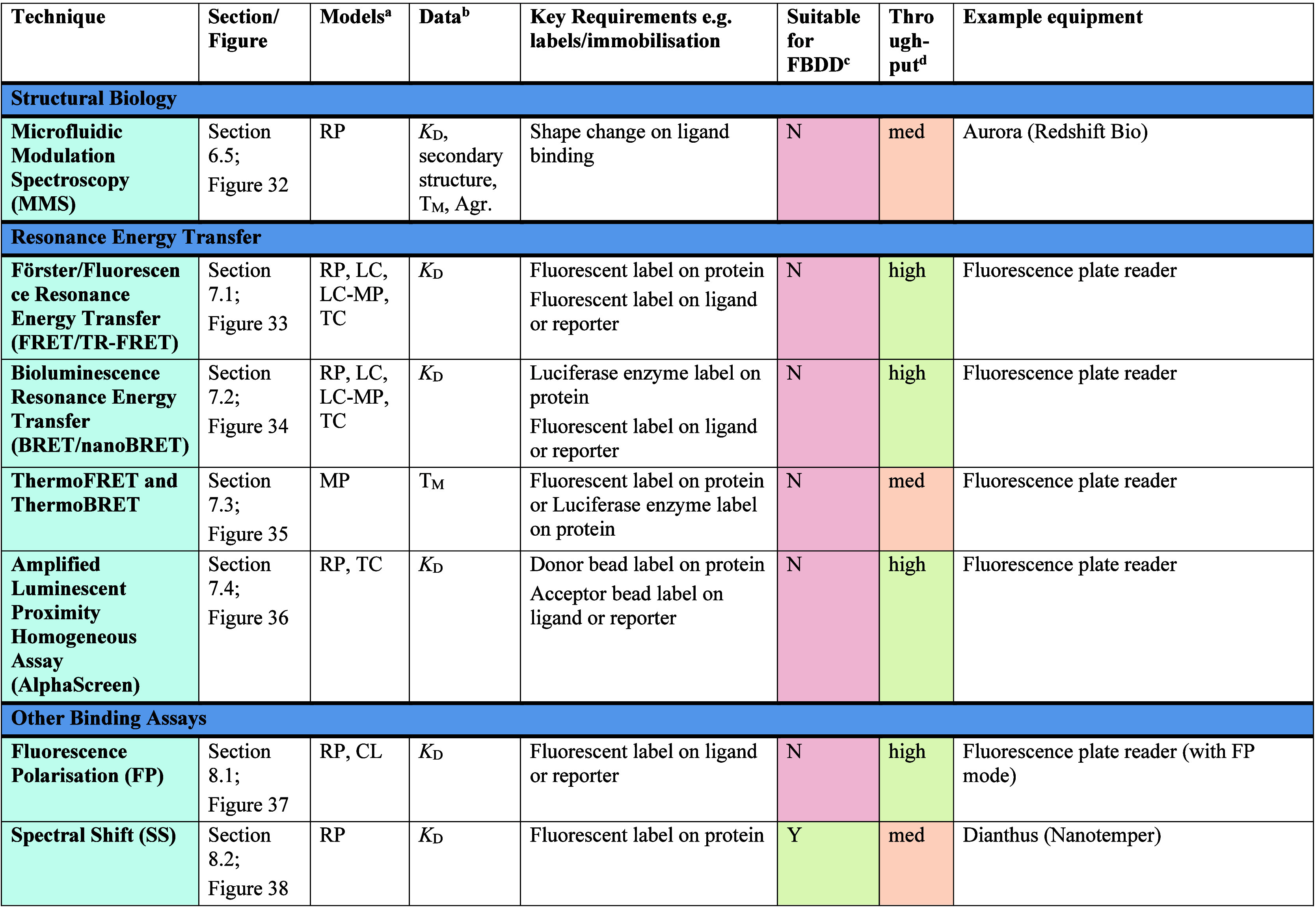

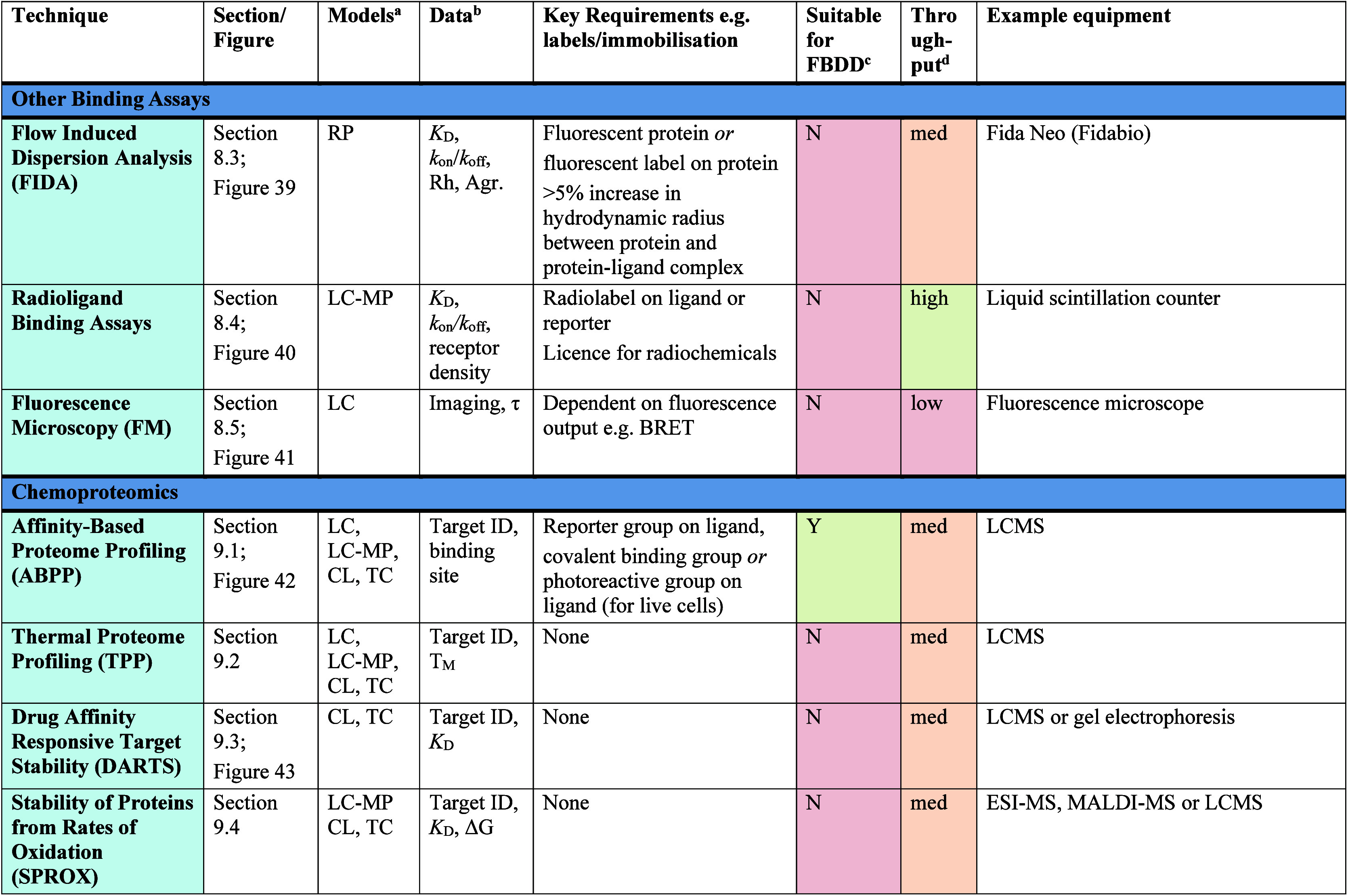
Summary of Methods to Observe Protein–Ligand Binding Covered in This Perspective

As Table 1 is a key feature of the Perspective, providing a rapid reference guide to all currently developed target engagement assays, correcting the table is highly important.

Note that the table title and caption are correct in the published manuscript and require no changes.

